# Quantal Ca^2+^ release mediated by very few IP_3_ receptors that rapidly inactivate allows graded responses to IP_3_

**DOI:** 10.1016/j.celrep.2021.109932

**Published:** 2021-11-02

**Authors:** Ana M. Rossi, Andrew M. Riley, Geneviève Dupont, Taufiq Rahman, Barry V.L. Potter, Colin W. Taylor

**Affiliations:** 1 Department of Pharmacology, University of Cambridge, Tennis Court Road, Cambridge CB2 1PD, UK; 2 Drug Discovery and Medicinal Chemistry, Department of Pharmacology, University of Oxford, Mansfield Road, Oxford OX1 3QT, UK; 3 Unité de Chronobiologie Théorique, Faculté des Sciences, CP231 Université Libre de Bruxelles (ULB), Boulevard du Triomphe, 1050 Brussels, Belgium

**Keywords:** Ca^2+^ signaling, endoplasmic reticulum, IP_3_ receptor, partial agonist, quantal Ca^2+^ release, receptor inactivation, intracellular Ca^2+^ stores, IP_3_ receptor antagonist

## Abstract

Inositol 1,4,5-trisphosphate receptors (IP_3_Rs) are intracellular Ca^2+^ channels that link extracellular stimuli to Ca^2+^ signals. Ca^2+^ release from intracellular stores is “quantal”: low IP_3_ concentrations rapidly release a fraction of the stores. Ca^2+^ release then slows or terminates without compromising responses to further IP_3_ additions. The mechanisms are unresolved. Here, we synthesize a high-affinity partial agonist of IP_3_Rs and use it to demonstrate that quantal responses do not require heterogenous Ca^2+^ stores. IP_3_Rs respond incrementally to IP_3_ and close after the initial response to low IP_3_ concentrations. Comparing functional responses with IP_3_ binding shows that only a tiny fraction of a cell’s IP_3_Rs mediate incremental Ca^2+^ release; inactivation does not therefore affect most IP_3_Rs. We conclude, and test by simulations, that Ca^2+^ signals evoked by IP_3_ pulses arise from rapid activation and then inactivation of very few IP_3_Rs. This allows IP_3_Rs to behave as increment detectors mediating graded Ca^2+^ release.

## Highlights

• IP_3_ evokes quantal Ca^2+^ release from the endoplasmic reticulum
• IP_3_ receptors rapidly activate and then inactivate
• Submaximal responses to IP_3_ require activation of very few IP_3_ receptors
• Rapid activation and inactivation of very few IP_3_Rs allow incremental responses to IP_3_

## Graphical abstract

**Figure:**
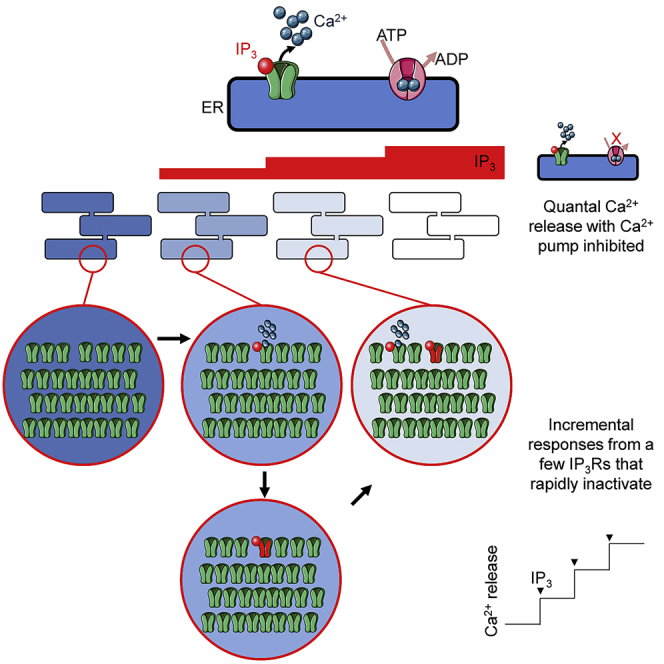

## Teaser

Rossi et al. define mechanisms for IP_3_-evoked quantal Ca^2+^ release that reconcile response termination with undiminished sensitivity to further IP_3_ additions. They show that responses require very few IP_3_ receptors that rapidly open and then inactivate, allowing graded responses despite the ability of IP_3_ receptors to propagate regenerative Ca^2+^ signals.

## Introduction

Inositol 1,4,5-trisphosphate (IP_3_) links receptors in the plasma membrane to Ca^2+^ release from the endoplasmic reticulum (ER) through IP_3_ receptors (IP_3_Rs), which are intracellular Ca^2+^ channels (Berridge, 2016). This redistribution of Ca^2+^ from the ER generates cytosolic Ca^2+^ signals, transfers Ca^2+^ to other organelles, and stimulates store-operated Ca^2+^ entry. IP_3_-evoked Ca^2+^ signals initiate at small clusters of IP_3_Rs, where IP_3_ binding primes IP_3_Rs to open in response to Ca^2+^ released by their neighbors (Smith and Parker, 2009; Thillaiappan et al., 2017, 2021). Similar Ca^2+^-induced Ca^2+^ release (CICR) occurs with ryanodine receptors (RyRs), the other major family of ER Ca^2+^ channels (Ríos, 2018). However, CICR is potentially explosive and might prevent cells from generating graded responses. Dispersed clusters of channels constrain regenerative propagation of Ca^2+^ signals between them (Ríos, 2018), and inhibition of IP_3_Rs (and RyRs) by increased cytosolic free Ca^2+^ concentration ([Ca^2+^]_c_) may contribute to terminating Ca^2+^ release, but it may not be the only mechanism (Wiltgen et al., 2014).

IP_3_-evoked Ca^2+^ release is “quantal”: submaximal IP_3_ concentrations rapidly release only a fraction of the Ca^2+^ stores before release terminates (Muallem et al., 1989; Taylor and Potter, 1990). This pattern of response occurs without compromising responses to further incremental increases in IP_3_ concentration (Meyer and Stryer, 1990), indicating that it is not mediated by a conventional form of desensitization. Quantal Ca^2+^ release by IP_3_Rs has been reported by many laboratories (reviewed in Bootman [1994] and Yamashita [2006]), and it is also a feature of RyRs (Cheek et al., 1994; Wang et al., 2004). Quantal responses have been most thoroughly examined in permeabilized cells, but they have also been reported in intact cells responding to stimuli that evoke IP_3_ formation (Bootman et al., 1992; Muallem et al., 1989). Hence, quantal Ca^2+^ release is an essential feature of both major families of ER Ca^2+^ channels.

**Figure 1:**
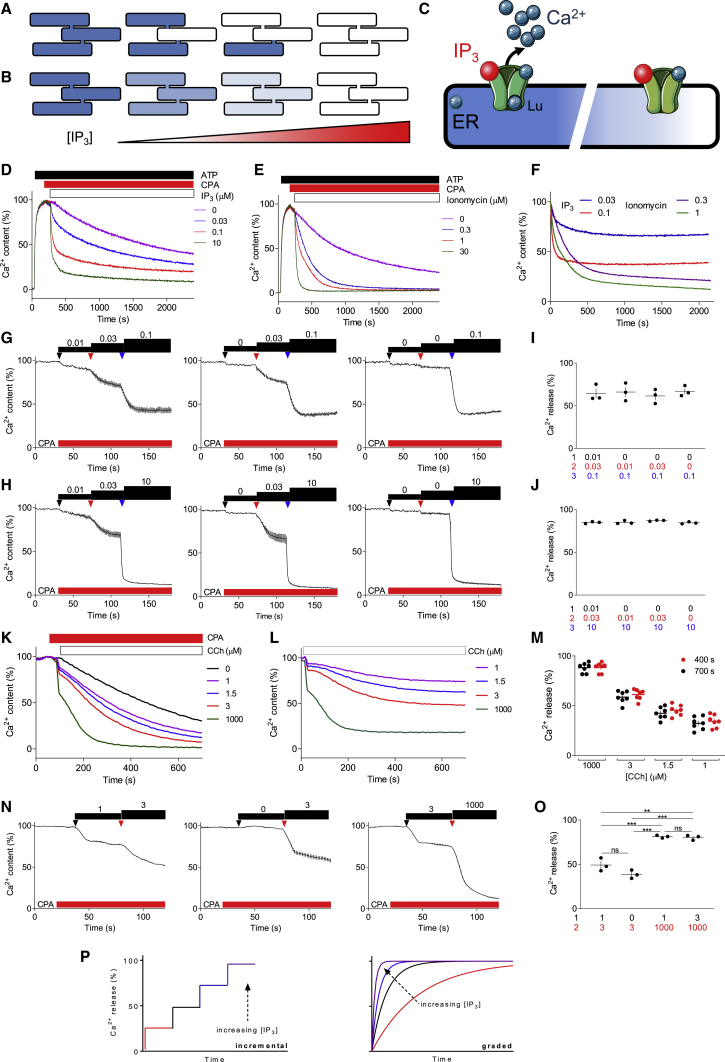
Quantal Ca^2+^ release by IP_3_Rs (A–C) Proposed mechanisms for quantal Ca^2+^ release: all-or-nothing emptying of stores with different IP_3_ sensitivities (A), or mechanisms that terminate Ca^2+^ release before stores have fully emptied (B). The latter could be mediated by luminal Ca^2+^ regulating IP_3_Rs, such that as the luminal [Ca^2+^] falls after IP_3_R activation, Ca^2+^ dissociates from the luminal site (Lu), and IP_3_Rs close trapping Ca^2+^ within the ER (C) (Irvine, 1990). (D and E) Ca^2+^ content of the ER of permeabilized Mag-fluo 4-loaded DT40-IP_3_R1 cells after addition of ATP (1.5 mM) followed by CPA (10 μM) to inhibit SERCAs, and then the indicated concentrations (μM) of IP_3_ (D) or ionomycin (E). Results (typical of three to four experiments, each with two replicates) show Ca^2+^ content (%) relative to steady-state Ca^2+^ content; (D) is reproduced from Rossi and Taylor (2020). (F) Responses to IP_3_ and ionomycin plotted after subtraction of the basal Ca^2+^ leak. (G and H) Effects of submaximal (100 nM) (G) or maximal (10 μM) (H) concentrations of IP_3_ added directly or by incremental additions to permeabilized DT40-IP_3_R1 cells. Results show mean ± SD of three replicates. (I and J) Summary results (individual values, mean ± SEM, n = 3, each with three replicates) show the Ca^2+^ released from the stores of permeabilized DT40-IP_3_R1 cells (determined at 150 s) for the three incremental additions (cumulative IP_3_ concentrations shown in μM). The final Ca^2+^ release was not significantly different between any of the incremental additions (one-way repeated ANOVA with Bonferroni’s multiple comparisons test). (K) Ca^2+^ content of the ER of intact wild-type HEK expressing G-CEPIA1*er* after addition of CPA (5 μM) to inhibit SERCAs, and then the indicated concentrations of carbachol (CCh, μM). Results (typical of seven experiments, each with three replicates) show Ca^2+^ content (%) relative to the Ca^2+^ content determined before adding CPA. (L) Responses to the indicated concentrations of carbachol in intact wild-type HEK expressing G-CEPIA1*er* after subtraction of the basal Ca^2+^ leak. (M) Summary results (mean ± SEM, n = 7, each with three replicates) show the Ca^2+^ released from the stores of intact wild-type HEK expressing G-CEPIA1*er* determined at 400 and 700 s for the indicated concentrations of carbachol. The Ca^2+^ release at 400 and 700 s was not significantly different at any of the carbachol concentrations (one-way repeated ANOVA with Bonferroni’s multiple comparisons test). (N) Incremental responses from intact wild-type HEK cells to the indicated concentrations of carbachol added after CPA (5 μM) and recorded using G-CEPIA1*er* (cumulative carbachol concentrations are shown in μM). (O) Summary results from intact wild-type HEK cells expressing G-CEPIA1*er* (individual values, mean ± SEM, n = 3, each with 8–12 replicates) show Ca^2+^ released from the stores (determined at 150 s) for the incremental additions (cumulative carbachol concentrations shown in μM). ^∗∗^p < 0.01, ^∗∗∗^p < 0.001; ns, not significant (for indicated comparisons; one-way repeated ANOVA with Bonferroni’s multiple comparisons test). (P) Incremental responses to IP_3_ compared with the graded rates of Ca^2+^ release expected if increasing concentrations of IP_3_ uniformly increased the permeability of the ER to Ca^2+^. See also Figures S1–S4.

The mechanism of quantal Ca^2+^ release is unknown, but it is not due to IP_3_ metabolism or compensatory Ca^2+^ re-uptake, nor does it require ATP (Meyer and Stryer, 1990; Taylor and Potter, 1990). Two categories of mechanism have been proposed. One suggests that submaximal concentrations of IP_3_ completely empty the stores that are most sensitive to IP_3_, and higher IP_3_ concentrations then recruit the less sensitive stores (Ferris et al., 1992; Hirose and Iino, 1994; Muallem et al., 1989; Oldershaw et al., 1991; Parker and Ivorra, 1990) (Figure 1A). This model requires both extremely cooperative responses to IP_3_ and compartmentalized Ca^2+^ stores with heterogenous sensitivities to IP_3_. The second model suggests that IP_3_R activity attenuates before Ca^2+^ stores are fully depleted (Figure 1B) (Irvine, 1990; Missiaen et al., 1992; Parys et al., 1993; Tregear et al., 1991). The mechanisms that might curtail IP_3_R-mediated Ca^2+^ release include IP_3_-mediated inactivation (Hajnóczky and Thomas, 1994), inhibition by increased [Ca^2+^]_c_, loss of the electrochemical Ca^2+^ gradient for Ca^2+^ release as Ca^2+^ leaves the ER without compensatory charge movements (Yamashita et al., 2006), and regulation of IP_3_Rs by luminal Ca^2+^ (Figure 1C). The latter mechanism proposes that opening of an IP_3_R requires binding of IP_3_ and of Ca^2+^ at both the cytosolic and luminal sides of the IP_3_R (Irvine, 1990; Marchant and Taylor, 1997). Regulation by luminal Ca^2+^ predicts that as ER [Ca^2+^] falls, the sensitivity of IP_3_Rs to cytosolic IP_3_ and Ca^2+^ declines, and Ca^2+^ release then terminates with Ca^2+^ trapped in the ER (Figure 1C). In the 30 years since quantal Ca^2+^ release was first identified (Muallem et al., 1989; Taylor and Potter, 1990), numerous studies have confirmed the phenomenon and provided evidence that supports or challenges each mechanism (Yamashita, 2006). Single-channel recordings from IP_3_Rs (Ionescu et al., 2006; Taufiq-Ur-Rahman et al., 2009) and high-resolution optical methods (Callamaras and Parker, 2000) have also failed to identify the mechanisms underlying incremental responses to IP_3_.

Here, we synthesize a high-affinity, partial agonist of IP_3_R with exceptionally low efficacy, and use it to establish that quantal responses are not mediated by all-or-nothing emptying of stores with heterogenous sensitivities to IP_3_ (Figure 1A). We show that all three subtypes of IP_3_Rs mediate incremental responses to IP_3_, and that the responses arise from a tiny fraction of a cell’s IP_3_Rs opening in response to IP_3_ and then inactivating. This mechanism resolves long-standing confusion and establishes how cells respond to incremental changes in IP_3_ concentration.

## Results and discussion

### Quantal Ca^2+^ release by all IP_3_R subtypes

To measure IP_3_-evoked Ca^2+^ release without the opposing activity of ER Ca^2+^ pumps (sarcoplasmic/ER Ca^2+^-ATPases [SERCAs]), the ER of permeabilized DT40 cells expressing only IP_3_R1 (DT40-IP_3_R1 cells) was loaded with Mag-fluo 4, a low-affinity Ca^2+^ indicator (Rossi and Taylor, 2020). ATP was added to fuel Ca^2+^ uptake before addition of cyclopiazonic acid (CPA) to inhibit SERCAs; IP_3_ was then added to stimulate Ca^2+^ release (Figure 1D). A maximally effective IP_3_ concentration released ∼70% of the ER Ca^2+^ content. Submaximal IP_3_ concentrations rapidly released a smaller fraction of the stores, after which there was no further effect of IP_3_ on the rate of Ca^2+^ release or its effect was much reduced (Figure 1D). These quantal responses to IP_3_ are clearest after correction for the basal Ca^2+^ leak evident after SERCA inhibition (Figure 1F). The responses to IP_3_ are very different to those evoked by the Ca^2+^ ionophore, ionomycin, which stimulated monophasic Ca^2+^ loss from the entire ER at rates that increased with ionomycin concentration (Figures 1E, 1F, S1A, and S1B). Similar quantal responses from type 1 IP_3_R (IP_3_R1) were observed with IP_3_ in the presence of the K^+^ ionophore, valinomycin, to dissipate any ER membrane potential; with Cs^+^ replacing K^+^ to inhibit K^+^ channels; with mitochondria inhibited; with IP_3_ added in larger volumes to avoid possible artifacts arising from bolus additions; and with inositol 2,4,5-trisphosphate ((2,4,5)IP_3_), a non-metabolized analog of IP_3_ (Hill et al., 1988) (Figures S1C–S1J).

Although responses to low IP_3_ concentrations rapidly attenuated (Figures 1D, 1F, and S1B), subsequent addition of more IP_3_ evoked further rapid Ca^2+^ release. Furthermore, the fraction of the Ca^2+^ stores released by maximal or submaximal IP_3_ concentrations was the same whether IP_3_ was delivered immediately at its final concentration or as incremental additions (Figures 1G–1J). Similar “incremental” responses (Beecroft and Taylor, 1997; Ferris et al., 1992; Meyer and Stryer, 1990) were obtained in the presence of the fast Ca^2+^ buffer, BAPTA, at a concentration (10 mM) sufficient to rapidly buffer even very local increases in [Ca^2+^]_c_ (Vais et al., 2012). This indicates that inhibition of IP_3_Rs by increases in [Ca^2+^]_c_ are not required for incremental responses (Figures S2A–S2F). That conclusion is consistent with two additional lines of evidence. First, IP_3_Rs from *Capsaspora owczarzaki* are not regulated by cytosolic Ca^2+^, but they do mediate quantal Ca^2+^ release (Alzayady et al., 2015). Second, in ER depleted of Ca^2+^, retrograde movement of Mn^2+^ through open IP_3_Rs can be used to report IP_3_R opening. Under these conditions, responses to IP_3_ have been reported to be quantal, but only after fragmentation of the ER (Hajnóczky et al., 1994; Renard-Rooney et al., 1993). Responses were also incremental in permeabilized DT40 cells expressing only IP_3_R2 or IP_3_R3 (Figures S2G–S2J), and in wild-type human embryonic kidney (HEK) cells, which express all three IP_3_R subtypes (Mataragka and Taylor, 2018) (Figure S3). In each case, the total amount of Ca^2+^ released by a submaximal concentration of IP_3_ was the same whether IP_3_ was presented as a single addition or as incremental additions. Incremental responses to IP_3_ were also observed using a low-affinity genetically encoded Ca^2+^ indicator targeted to the ER lumen (G-CEPIA1*er*) (Suzuki et al., 2014) (Figures S4A–S4H).

In single-cell analyses of permeabilized HEK cells stably expressing G-CEPIA1*er*, all cells responded to a maximal IP_3_ concentration with a rapid decrease in ER luminal [Ca^2+^]. Submaximal IP_3_ concentrations rapidly released a fraction of the IP_3_-sensitive stores, and the response then terminated without preventing subsequent responses to a maximal IP_3_ concentration (Figures S4I–S4N). Quantal responses observed in populations of permeabilized cells are not, therefore, due to all-or-nothing responses from different cells with heterogeneous IP_3_ sensitivities. In permeabilized hepatocytes too, quantal responses to IP_3_ were observed in single cells (Renard-Rooney et al., 1993). We also measured changes in G-CEPIA1*er* fluorescence within small subcellular regions and, although the spatial resolution was limited, the results suggest that different regions within a HEK cell have similar sensitivity to IP_3_ (Figures S4K–S4N).

**Figure 2:**
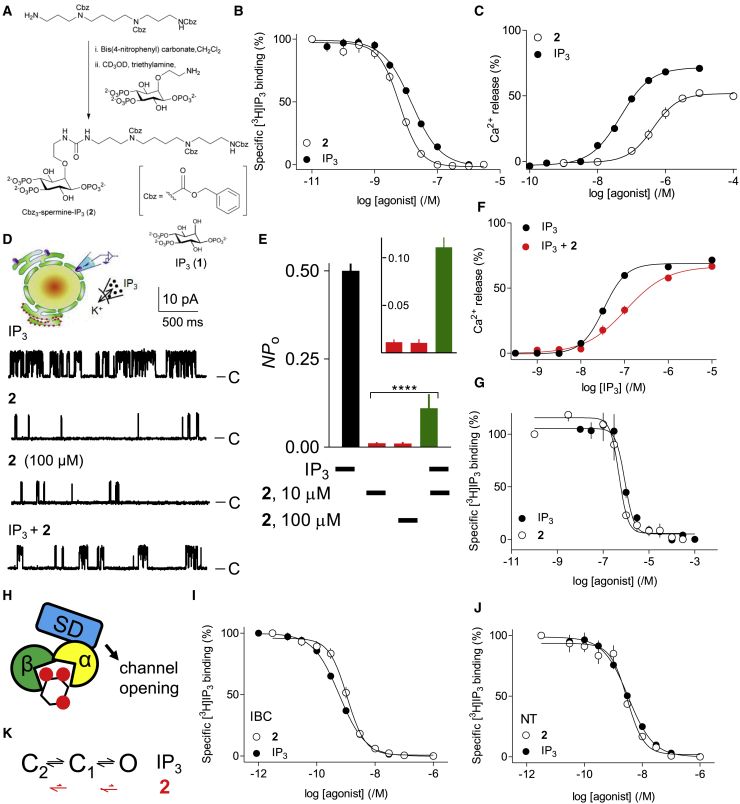
A 2-*O*-modified analog of IP_3_ (**2)** is a high-affinity, partial agonist (A) Synthetic scheme for **2** and structure of IP_3_. (B) Equilibrium-competition binding to purified full-length IP_3_R1 using [^3^H]IP_3_ (1.5 nM) and the indicated concentrations of IP_3_ or **2** in Tris-EDTA medium (TEM) at 4°C (mean ± SEM, n = 7, many error bars are smaller than the symbols). (C) Effects of IP_3_ or **2** on Ca^2+^ release (determined after 20 s) from permeabilized DT40-IP_3_R1 cells (mean ± SEM, n = 3, each with three replicates). (D) Patch-clamp recordings, typical of at least five recordings, from excised nuclear patches of DT40-IP_3_R1 cells, at a holding potential of +40 mV with K^+^ as the charge carrier, and ligands within the pipette (cytosolic surface; IP_3_, 10 μM; **2**, 10 μM, except where shown otherwise). C, closed state. (E) Summary results (mean ± SEM, n = 5–7) show channel activity (*NP*_o_) for the indicated stimuli (enlarged in the inset). ^∗∗∗∗^p < 0.0001, ANOVA with Bonferroni’s test, relative to IP_3_ alone. (F) Permeabilized DT40-IP_3_R1 cells stimulated (30 s) with **2** (1 μM, which evoked 31% ± 7% Ca^2+^ release) were then stimulated with the indicated concentrations of IP_3_. Ca^2+^ release evoked by IP_3_ (determined after a further 20 s) is shown as a percentage of the store content after the first stimulus (mean ± SEM, n ≥ 4, each with three replicates). (G) Equilibrium-competition binding to cerebellar membranes using [^3^H]IP_3_ (7.5 nM) and the indicated concentrations of IP_3_ or **2** in CLM at 20°C (mean ± SEM, n = 3–4 with duplicate determinations). pK_D_ values were 6.10 ± 0.06 (mean ± SEM, n = 4, K_D_ = 794 nM) for IP_3_ and 6.26 ± 0.01 (n = 3, K_D_ = 549 nM) for **2**. Hill coefficients (*h*) were 3.2 ± 1.3 and 2.0 ± 0.7 for IP_3_ and **2**. (H) IP_3_ binds between the α and β domains of the IBC, but the SD is required for IP_3_ to evoke channel opening. (I and J) Equilibrium-competition binding of IP_3_ and **2** to the IBC or NT using [^3^H]IP_3_ (0.3 or 0.75 nM for IBC and NT, respectively). Mean ± SEM, n = 4. (K) IP_3_R moves between an unknown numbers of closed states (C) to an open state (O) after IP_3_ binding. Rate(s) of movement through the closed to the open state occur more slowly with **2** bound to the IP_3_R. Tables S1–S3 summarize the properties of IP_3_ and **2**.

**Figure 3:**
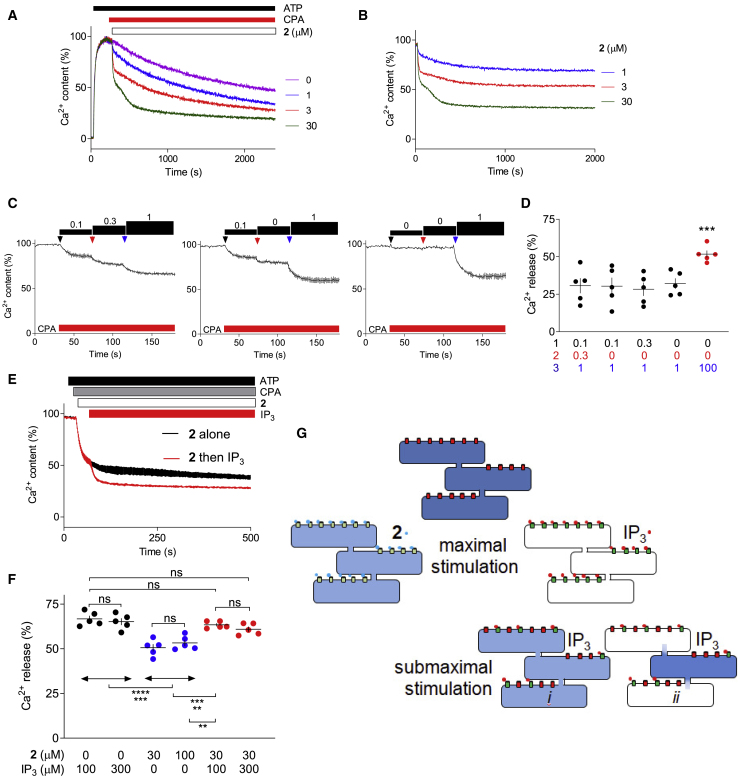
Quantal responses are not mediated by heterogenous stores (A) Ca^2+^ content of the ER of populations of permeabilized DT40-IP_3_R1 cells after addition of ATP, CPA (10 μM), and then the indicated concentrations of **2**. Results are typical of three experiments, each with duplicate determinations. (B) Responses to **2** shown after subtraction of the basal Ca^2+^ leak. (C) Effects of a submaximal concentration of **2** (1 μM) added directly or by incremental additions. Results show mean ± SD of three replicates. (D) Summary results (individual values, mean ± SEM, n = 5, each with three replicates) show the final Ca^2+^ content of the stores of permeabilized DT40-IP_3_R1 cells (determined at 150 s). A maximal concentration of **2** (30 μM) released 52% ± 3% of the Ca^2+^ stores (red symbols). ^∗∗∗^p < 0.001, one-way repeated ANOVA with Bonferroni’s test relative to all other values. (E) Ca^2+^ release from wild-type HEK cells evoked by **2** (30 μM) alone or followed by a supra-maximal concentration of IP_3_ (100 μM). Mean of duplicate determinations. (F) Summary results (individual values, mean ± SEM, n = 5) show Ca^2+^ release (determined at 500 s) evoked by the indicated concentrations of **2** followed by IP_3_ in wild-type HEK cells. ^∗∗^p < 0.01, ^∗∗∗^p < 0.001,^∗∗∗∗^p < 0.0001; ns, not significant (for indicated comparisons, ANOVA with Bonferroni’s test). (G) Maximal IP_3_ concentrations fully empty the IP_3_-sensitive Ca^2+^ stores, but it was unclear whether quantal responses to submaximal IP_3_ concentrations arise from incomplete emptying of the entire ER (*i*) or complete emptying of a fraction of the ER (*ii*). Results with a maximally effective concentration of **2**, which partially activates all IP_3_Rs leaving Ca^2+^ trapped within the ER, indicate that quantal responses are not due to all-or-nothing emptying of heterogenous stores (*ii*).

It has been reported that quantal responses to IP_3_ are observed in permeabilized hepatocytes only when permeabilization is accompanied by fragmentation of the ER (Renard-Rooney et al., 1993). Most studies do not address whether the ER remains continuous after permeabilization. In our analyses of permeabilized HEK cells, there is some fragmentation of the ER, whether reported by G-CEPIA1*er* or compartmentalized Mag-fluo 4, and attempts to avoid it by adjusting conditions failed to achieve ATP-dependent Ca^2+^ uptake without some ER fragmentation (Figure S4O). However, several lines of evidence establish that quantal responses are not an artifact arising from ER fragmentation. First, others have observed quantal responses from stimuli that evoke IP_3_ formation in intact cells (Bootman et al., 1992; Muallem et al., 1989). By measuring quantal responses directly using a Ca^2+^ indicator within the ER lumen, we confirmed that stimulation of the endogenous muscarinic acetylcholine receptors of HEK cells with carbachol to evoke IP_3_ formation caused quantal Ca^2+^ release from intracellular stores (Figures 1K–1M). Successive additions of carbachol evoked incremental responses (Figures 1N and 1O). Second, fragmentation of the ER might create the heterogenous Ca^2+^ stores required for the all-or-nothing model of quantal Ca^2+^ release (Figure 1A), but it is difficult to envisage how it might create the conditions required for a model where IP_3_Rs close before the stores have fully emptied (Figure 1B). Our subsequent experiments demonstrate that all-or-nothing emptying of discrete Ca^2+^ stores cannot explain quantal responses (see Figures 2 and 3), and we provide direct evidence that IP_3_Rs inactivate before the stores have emptied (see Figures 5, 6, and 7).

The results so far confirm that incremental responses to IP_3_ occur within single cells, they are not a consequence of cell permeabilization, and they are not mediated by increases in [Ca^2+^]_c_, by IP_3_ metabolism, or by ineffective movement of counter-ions, nor do mitochondria contribute. Incremental responses to IP_3_ (Figure 1P) are a feature of all IP_3_R subtypes, and genetically encoded IP_3_R heterogeneity is not required for quantal Ca^2+^ release. The present results do not, however, distinguish between the proposed mechanisms (Figures 1A–1C).

### Characterization of a high-affinity, weak partial agonist of IP_3_Rs

Because partial agonists activate receptors less effectively than do full agonists (Rossi et al., 2009), we predicted that if a weak partial agonist occupied all IP_3_Rs and evoked quantal Ca^2+^ release, the quantal mechanism could not be due to heterogenous IP_3_R sensitivity. We previously developed 2-*O*-modified IP_3_ analogs that are partial agonists (Rossi et al., 2009), but none has sufficiently low efficacy for our present needs. Reasoning that enlarging the 2-*O*-substituent might further reduce efficacy, we synthesized additional analogs and found Cbz_3_-spermine-IP_3_ (compound **2**) to be an exceptionally weak partial agonist (Figure 2A). Compound **2** bound to IP_3_R1 with about 2-fold greater affinity than IP_3_ (Figure 2B), but it was significantly less potent than IP_3_ in stimulating Ca^2+^ release from permeabilized DT40-IP_3_R1 cells (Figures 2C; Table S1) or wild-type HEK cells (Table S2). The difference between binding and functional analyses is captured by comparing the ligand concentrations required to release 50% of the IP_3_-sensitive Ca^2+^ stores (EC^I^_50_) and to occupy 50% of IP_3_-binding sites (K_D_, equilibrium dissociation constant); the ratio (EC^I^_50_/K_D_) is 41-fold greater for **2** than for IP_3_ (Table S1). The weakest known partial agonist with an affinity comparable to IP_3_ (compound 4 in Rossi et al., 2009) has an EC^I^_50_/K_D_ ratio only 5.7-fold greater than that of IP_3_.

In patch-clamp recordings from the outer nuclear envelope, which is continuous with the ER, the increase in channel open probability (*NP*_o_) of IP_3_R1 was the same for 10 and 100 μM of **2**, confirming that both concentrations were maximally effective (Figures 2D and 2E). However, the maximal *NP*_o_ for **2** was 40-fold less than for IP_3_; the single-channel conductance (γ_K_) was the same for both agonists (Figures 2D and 2E; Table S1). Because partial agonists bind to the same site as full agonists, but less effectively activate the receptor, they behave as competitive antagonists of full agonists. This behavior is evident in patch-clamp and Ca^2+^-release assays, where **2** reduced the response to IP_3_ (Figures 2D–2F). These observations, which are expected for a partial agonist, are important for subsequent experiments because they demonstrate that IP_3_ and **2** compete for occupancy of the same IP_3_Rs, but **2** causes less effective activation.

The K_D_ of **2** determined from dose-ratio analysis (K_D_ = 426 nM) (Table S3) was similar to the half-maximally effective concentration of **2** in Ca^2+^-release assays (EC_50_ = 479 nM, Table S1), as expected for a weak partial agonist. The K_D_ for **2** derived from functional assays (∼450 nM) is much greater than that determined by radioligand binding (5.2 nM) because they use different assay conditions (Ding et al., 2010). However, in binding analyses performed under conditions that mimic functional assays, the K_D_ for **2** was 549 nM, which is comparable to its EC_50_ for Ca^2+^ release, and 1.5-fold lower than the K_D_ for IP_3_ determined under these conditions (794 nM) (Figure 2G).

We reported previously that the reduced open probability (*P*_o_) of IP_3_Rs activated by partial agonists was due to an increase in mean channel closed time (τ_c_), with no effect on mean open time (τ_o_) (Rossi et al., 2009). Similar behavior appears to underlie the reduced efficacy of **2** because the modest (∼2-fold) decrease in τ_o_ (relative to IP_3_) is insufficient to explain the 40-fold decrease in *NP*_o_ (Table S1). With such low *P*_o_, it is impracticable to determine τ_c_ directly for **2**.

IP_3_ binds to the IP_3_-binding core of the IP_3_R (IBC, residues 224–604) (Bosanac et al., 2002), but communication with the channel requires the suppressor domain (SD, residues 1–223) (Rossi et al., 2009; Yoshikawa et al., 1999) (Figure 2H). The difference in ligand affinities (ΔΔ*G* = Δ*G*^IBC^ − Δ*G*^NT^, where Δ*G* = −RT*ln*K_D_) for the IBC and N-terminal (NT, residues 1–604) reports the binding energy diverted into changing the NT conformation (Rossi et al., 2009). Analyses of IP_3_ and **2** binding to the IBC and NT confirm that IP_3_ diverts more energy into changing the NT conformation than does **2** (Figures 2I and 2J; Table S1).

We conclude that **2** is a high-affinity, partial agonist of IP_3_R. It is the weakest known partial agonist with high affinity for IP_3_R. The basis of its low efficacy is similar to that of related partial agonists (Rossi et al., 2009): it perturbs communication between the IBC and SD, causing the channel to dwell longer in a closed state and so open infrequently (Figure 2K). Our results suggest strategies to develop high-affinity antagonists of IP_3_R and they provide the tool needed to assess whether all-or-nothing emptying of Ca^2+^ stores with heterogenous IP_3_R sensitivities underlies quantal responses (Figure 1A).

### Quantal responses are not mediated by heterogenous stores

Compound **2** stimulated quantal Ca^2+^ release, and the responses to sequential additions were incremental (Figures 3A–3D). However, the quantal response evoked by a maximally effective concentration of **2** (Figures 3A and 3D) was smaller than the maximal response to IP_3_ (Figure 2C). Hence, even when all IP_3_Rs are occupied by **2**, the IP_3_-sensitive Ca^2+^ stores are not fully depleted. However, the Ca^2+^ stores remain sensitive to a subsequent IP_3_ addition, albeit at a high concentration of IP_3_ because it must now surmount the competitive antagonism of **2** (Figures 3E and 3F). Since **2** evokes quantal Ca^2+^ release when it occupies all IP_3_Rs, the quantal phenomenon cannot be due to all-or-nothing emptying of stores with heterogenous sensitivities. The response to a maximal concentration of **2** in Figure 3A is biphasic, and we also observed some biphasic responses to IP_3_ (see Figures S5L and S5M), but such biphasic responses were not consistently observed and were not further analyzed.

When cells are maximally stimulated with **2**, IP_3_ can bind to IP_3_R only after **2** dissociates. Rapid IP_3_-evoked Ca^2+^ release after maximal stimulation with **2** (Figures 3E and 3F) therefore demonstrates that quantal Ca^2+^ release is accompanied by rapid dissociation and re-association of agonists: IP_3_ is not “locked” onto activated IP_3_Rs. We conclude that during quantal responses, there is incomplete emptying of the entire ER, and IP_3_ continues to associate with and dissociate from IP_3_Rs (Figures 1B and 3G).

### Quantal Ca^2+^ release is not a property of the ER

**Figure 4:**
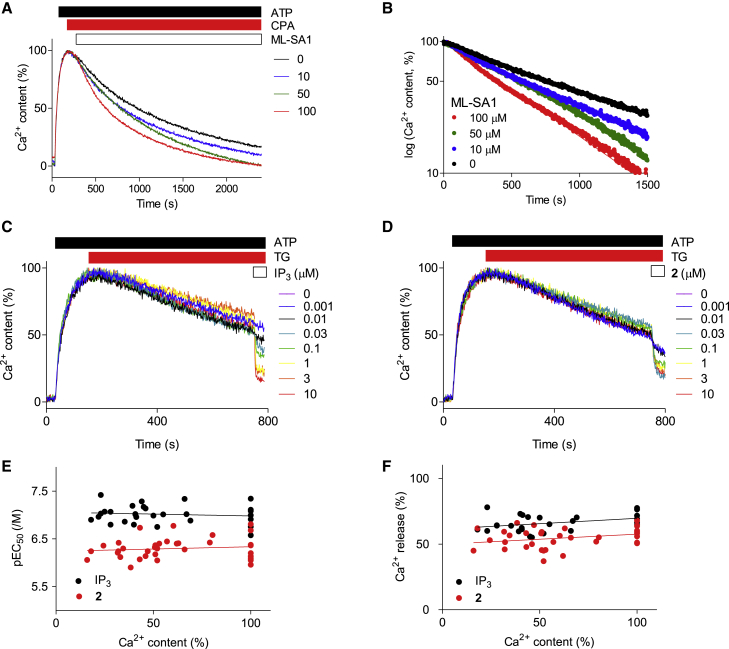
Neither ER reorganization nor luminal Ca^2+^ mediates quantal Ca^2+^ release (A) Effects of the indicated concentrations of ML-SA1 on the ER Ca^2+^ content of permeabilized HEK cells expressing CFP-TRPML1. (B) Semi-logarithmic plots show mono-exponential loss of ER Ca^2+^ after treatment with ML-SA1. Results (A and B) are typical of four experiments, each with duplicate determinations. Summary results are in Figure S5B. (C and D) Permeabilized DT40-IP_3_R1 cells were loaded to steady state with Ca^2+^ before addition of thapsigargin (TG, 1 μM) to inhibit SERCAs. At intervals thereafter (0–35 min), IP_3_ (C) or **2** (D) was added. Results are typical of 29–36 experiments. (E and F) Summary results (each point from a single concentration-effect relationship) show the relationships between ER Ca^2+^ content at the time of addition of IP_3_ or **2** (%) and pEC_50_ value (−logEC_50_) (E) and maximal Ca^2+^ release (F). Lines (least-squares linear regression) have slopes that are not significantly different from 0. See also Figure S5.

Since quantal Ca^2+^ release occurs with IP_3_Rs (Figure 1) (Yamashita, 2006) and RyRs (Cheek et al., 1994; Wang et al., 2004), it might reflect properties of the ER rather than its channels. Increases in [Ca^2+^]_c_ can, for example, restructure the ER (Subramanian and Meyer, 1997), suggesting that loss of Ca^2+^ from the ER might cause its further fragmentation and deny active Ca^2+^ channels access to the remaining Ca^2+^. Transient receptor potential mucolipin 1 (TRPML1) channels are usually expressed in the membranes of lysosomes (Shen et al., 2012), but when overexpressed TRPML1 channels also release Ca^2+^ from the ER and some TRPML1 populates the reticular ER in which IP_3_Rs are expressed (Figures 4A and S5A–S5F). In permeabilized HEK cells expressing CFP-TRPML1, but not in mock-transfected cells, ML-SA1 (a selective TRPML1 agonist) (Shen et al., 2012) stimulated a concentration-dependent Ca^2+^ release from the ER (Figures 4A and S5A). In contrast to the quantal responses evoked by IP_3_ and **2**, ML-SA1 caused a concentration-dependent increase in the rate of Ca^2+^ release from the entire ML-SA1-sensitive ER (Figures 4B and S5B). These results establish that quantal Ca^2+^ release is not a property of the ER or an inexorable consequence of cell permeabilization with the associated ER fragmentation (Figure S4O), but it is instead a feature of IP_3_Rs and RyRs. That conclusion is consistent with evidence that purified IP_3_Rs mediate quantal Ca^2+^ release (Ferris et al., 1992).

### Neither luminal Ca^2+^ nor erlin 2 determines quantal responses

An appealing hypothesis is that luminal Ca^2+^ is required for IP_3_R gating, such that as the ER loses Ca^2+^ the IP_3_R channel closes trapping Ca^2+^ within the ER (Irvine, 1990) (Figure 1C). Previous analyses of the effects of luminal Ca^2+^ provide conflicting results. There is evidence that a very substantial loss of ER Ca^2+^ reduces IP_3_R sensitivity (Barrero et al., 1997; Marshall and Taylor, 1994) and that overloading stores with Ca^2+^ promotes Ca^2+^ release (Missiaen et al., 1992), while other reports suggest that luminal Ca^2+^ reduces IP_3_R sensitivity (Vais et al., 2020) or has no effect (Beecroft and Taylor, 1997; Shuttleworth, 1992).

We anticipated that if three ligands are required to open IP_3_Rs (IP_3_, cytosolic Ca^2+^, and luminal Ca^2+^, Figure 1C), channel opening by IP_3_ might require less luminal Ca^2+^ than the weak partial agonist **2**. We used thapsigargin to irreversibly inhibit SERCA and slowly drain the ER of Ca^2+^, and then evaluated responses to IP_3_ and **2** at different ER Ca^2+^ contents (Figures 4C and 4D). The fraction of the remaining Ca^2+^ stores released by IP_3_ or **2** and the EC_50_ for each ligand were unaffected as stores lost their Ca^2+^. We conclude that there is no significant effect of ER Ca^2+^ content on the sensitivity or maximal response to IP_3_ or **2** (Figures 4E and 4F), even when the Ca^2+^ content is reduced to levels well below those observed after quantal responses to IP_3_ or **2**. Luminal Ca^2+^ does not, therefore, affect the number of open IP_3_Rs. We conclude, and consistent with some previous reports (Beecroft and Taylor, 1997; Combettes et al., 1992; Hajnóczky and Thomas, 1994; Oldershaw et al., 1991; Shuttleworth, 1992), that regulation of IP_3_Rs by luminal Ca^2+^ does not underlie quantal responses.

Activation of IP_3_Rs initiates a sequence that can lead to their proteasomal degradation. An early step in this sequence is recognition of active IP_3_Rs by an ER-membrane protein, erlin 2 (ER lipid raft-associated protein 2) (Wojcikiewicz, 2018). We considered whether erlin 2 might associate with active IP_3_Rs, terminate their activity, and thereby contribute to quantal Ca^2+^ release. However, substantial depletion of erlin 2 using small interfering RNA (siRNA) had no effect on quantal responses to IP_3_ (Figures S5G–S5M). We conclude that neither erlin 2 nor changes in ER luminal [Ca^2+^] contribute to incremental Ca^2+^ release by IP_3_.

### IP_3_ evokes Ca^2+^ release and then IP_3_R inactivation

Previous analyses of IP_3_R inactivation provide conflicting results, perhaps indicating a need for unidentified labile accessory factors (Bezprozvanny and Ehrlich, 1994; Hajnóczky and Thomas, 1994; Mak and Foskett, 1997; Marchant and Taylor, 1998; Oldershaw et al., 1992; Taufiq-Ur-Rahman et al., 2009; Stehno-Bittel et al., 1995). To examine any possible contribution of IP_3_R inactivation to quantal Ca^2+^ release, we therefore examined IP_3_R inactivation under conditions that exactly replicate those we used to show incremental responses to IP_3_.

**Figure 5:**
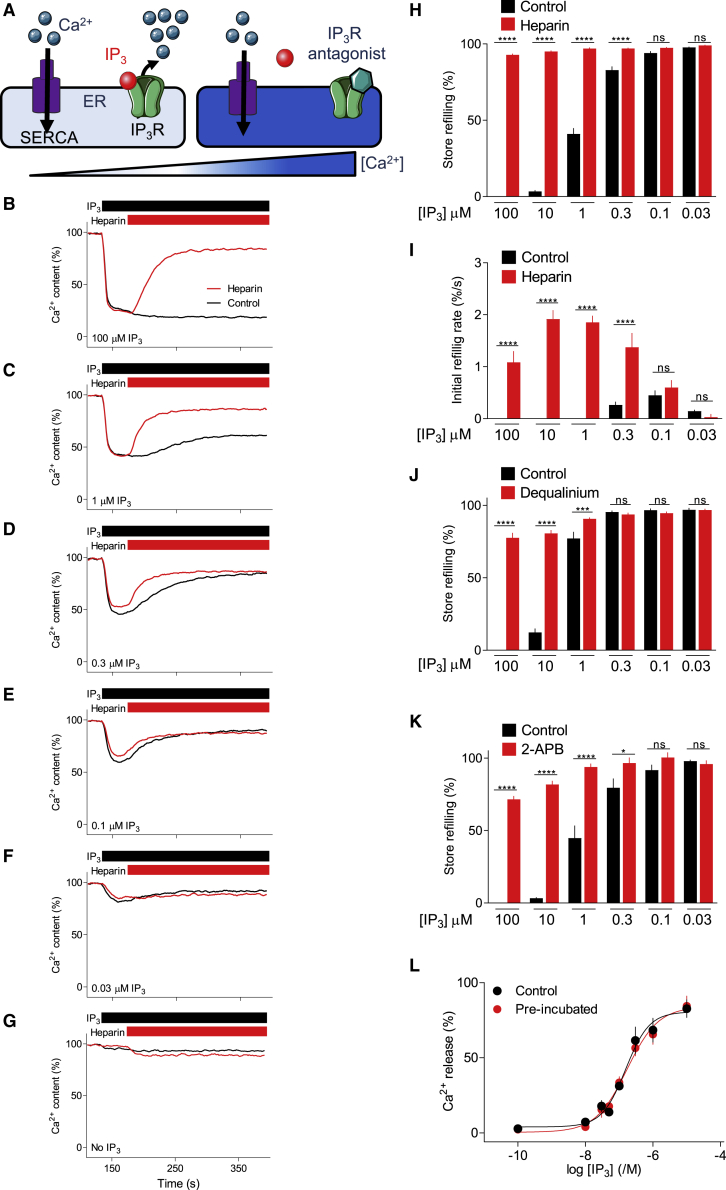
IP_3_Rs close after rapid Ca^2+^ release evoked by low IP_3_ concentrations (A) If IP_3_Rs remain active after an incremental Ca^2+^ release; stores should re-accumulate Ca^2+^ after addition of an IP_3_R antagonist. (B–G) The ER of permeabilized HEK-IP_3_R1 cells was loaded with Ca^2+^ by addition of ATP (t = 0); IP_3_ (0–30 μM) and heparin (10 mg/mL) were then added as indicated. Each trace (mean of duplicate determinations) shows results with a different IP_3_ concentration. The code in (B) applies to all panels. (H and I) Summary results (mean ± SEM, n = 6, each with two determinations) show the extent to which stores have refilled (relative to cells without IP_3_) 250 s after IP_3_ addition (H), as well as the initial rate of refilling from the slope of the curve immediately after heparin addition (I). In (H), 0% refilling is defined as the store content in control conditions measured 250 s after addition of a maximal IP_3_ concentration. In these experiments, the EC_50_ for IP_3_-evoked Ca^2+^ release was 166 nM (pEC_50_ = 6.78 ± 0.08) and maximal Ca^2+^ release was 86% ± 2% (mean ± SEM, n = 6). (J and K) Summary results (mean ± SEM, n = 5–7, each with duplicate determinations) from similar analyses using 50 μM dequalinium (J; Figures S7A–S7F) or 125 μM 2-APB (K; Figures S7G–S7L) to block IP_3_Rs. ^∗^p < 0.05, ^∗∗∗^p < 0.001, ^∗∗∗∗^p < 0.0001, one-way repeated ANOVA with Bonferroni’s test for the indicated comparisons (H–K). (L) IP_3_ was incubated with permeabilized HEK-IP_3_R1 cells (pre-incubated) or without cells (control) for 260 s under conditions identical to those used to show IP_3_R inactivation. The supernatant was then bio-assayed. Summary results (mean ± SEM, n = 4, each with three replicates) show no significant difference between the potency of IP_3_ after incubation under control conditions or with permeabilized cells. The results establish that refilling of stores during sustained incubation with low concentrations of IP_3_ is not due to its metabolism. Table S4 provides a summary and detailed methods. See also Figures S6 and S7 and Table S4.

To assess the activity of IP_3_Rs during the sustained phase of incremental responses, we examined IP_3_-evoked Ca^2+^ release without inhibiting SERCAs and then added an IP_3_R antagonist during the response. The rationale is that if IP_3_Rs remain active, an antagonist should reverse any ongoing activity and allow stores to refill (Figure 5A). We used three unrelated antagonists since each has limitations (Figure S6). Heparin is a competitive antagonist (Richardson and Taylor, 1993) and 2-aminoethoxydiphenyl borate (2-APB) is a non-competitive inhibitor with some selectivity for IP_3_R1 (Saleem et al., 2014). We show that dequalinium, which blocks K^+^ channels, is also an IP_3_R antagonist (Figures S6A–S6G). At the concentrations used, the antagonists completely (heparin and dequalinium) or partially (2-APB) blocked IP_3_-evoked Ca^2+^ release from permeabilized HEK-IP_3_R1 cells (Figures S6H–S6J).

**Figure 6:**
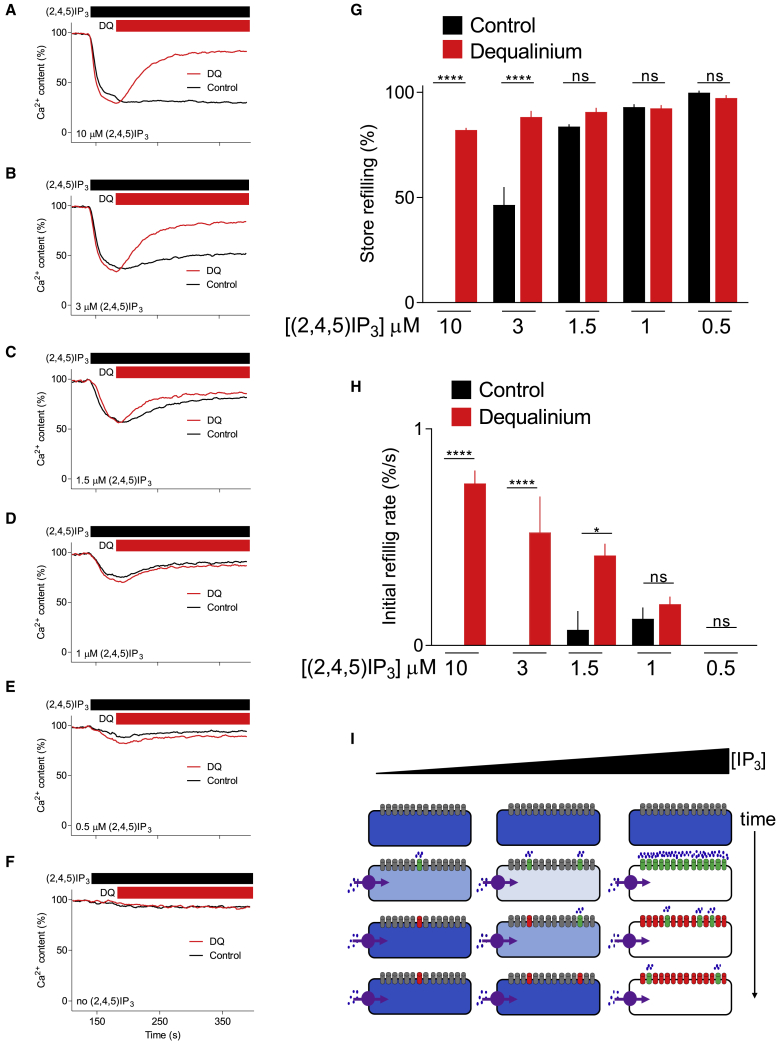
IP_3_Rs of HEK-IP_3_R1 cells close after incremental responses to low concentrations of a stable analog of IP_3_ (A–F) The experiments are similar to those shown in Figure 5, but using HEK-IP_3_R1 cells, (2,4,5)IP_3_ at the indicated concentrations, and dequalinium (DQ, 50 μM). Permeabilized cells were loaded with Ca^2+^ by addition of ATP before adding the indicated concentrations of (2,4,5)IP_3_ (without inhibiting SERCAs) and then the IP_3_R antagonist, dequalinium. Traces show means of two replicates. (G and H) Summary results (mean ± SEM, n = 4, each with two determinations) show the extent to which stores refill (relative to cells without (2,4,5)IP_3_) 250 s after (2,4,5)IP_3_ addition (G), and the initial rate of refilling from the slope of the curve immediately after DQ addition (H). ^∗^p < 0.05, ^∗∗∗∗^p < 0.0001, one-way ANOVA with Bonferroni’s test. (I) Stores rapidly refill after stimulation with the lowest IP_3_ concentrations, as the very few active IP_3_Rs inactivate. Higher concentrations of IP_3_ activate many more IP_3_Rs than needed to empty the stores, hence even substantial inactivation leaves enough active IP_3_Rs to keep the stores empty. See also Figures S6 and S7.

During sustained stimulation of permeabilized HEK-IP_3_R1 cells with a maximal concentration of IP_3_, the stores remained empty, but they refilled after adding heparin (Figure 5B). The incomplete refilling is probably due to partial inhibition of SERCAs by heparin (Figures S6K–S6M). The results demonstrate that during maximal stimulation, enough IP_3_Rs remain open to counteract opposing SERCA activity. Since an IP_3_R can conduct ∼500,000 Ca^2+^/s (Vais et al., 2010) while a SERCA transports fewer than 40 Ca^2+^/s (Lytton et al., 1992), it may require very little residual IP_3_R activity to overwhelm SERCAs and keep the ER depleted of Ca^2+^. After stimulation with the lowest IP_3_ concentrations (30–100 nM), the stores rapidly refilled and the rate was unaffected by heparin (Figures 5E–5I). This indicates that after the initial Ca^2+^ release evoked by low IP_3_ concentrations, there were no detectable open IP_3_Rs. The pattern of response to intermediate concentrations of IP_3_ fell between these extremes: there was residual sustained IP_3_R activity with the higher IP_3_ concentrations, and IP_3_R inactivation occurred with the lower concentrations, but more slowly than for the lowest IP_3_ concentration (Figures 5C, 5D, 5H, and 5I). Similar results were obtained with different antagonists (Figures 5J, 5K, and S7), with heparin in wild-type HEK cells (Figures S7M–S7T), and with a metabolically stable analog of IP_3_, (2,4,5)IP_3_ (Figures 6A-6H). We also used a bioassay of IP_3_ to confirm that refilling of Ca^2+^ stores during prolonged incubation with low concentrations of IP_3_ was not due to IP_3_ degradation (Figure 5L; Table S4).

Previous studies provide conflicting evidence for IP_3_-evoked IP_3_R inactivation. Single-channel recordings of IP_3_Rs support (Mak and Foskett, 1997; Stehno-Bittel et al., 1995) or challenge inactivation (Bezprozvanny and Ehrlich, 1994; Taufiq-Ur-Rahman et al., 2009), and while some analyses of permeabilized cells show IP_3_-evoked inactivation (Hajnóczky and Thomas, 1994; Marchant and Taylor, 1998), others suggest that responses are sustained (Oldershaw et al., 1992). Our results, from analyses where incremental responses and inactivation were assessed under identical conditions, show that incremental responses to low IP_3_ concentrations are accompanied by rapid inactivation of IP_3_Rs (Figure 6I).

### Incremental responses involve activation of very few IP_3_Rs

If quantal responses to low IP_3_ concentrations are terminated by closure of IP_3_Rs, how can stores retain undiminished responsiveness to further IP_3_ additions? Comparison of the relationship between the occupancy of IP_3_Rs by IP_3_ with functional responses provides a possible answer to this conundrum.

**Figure 7:**
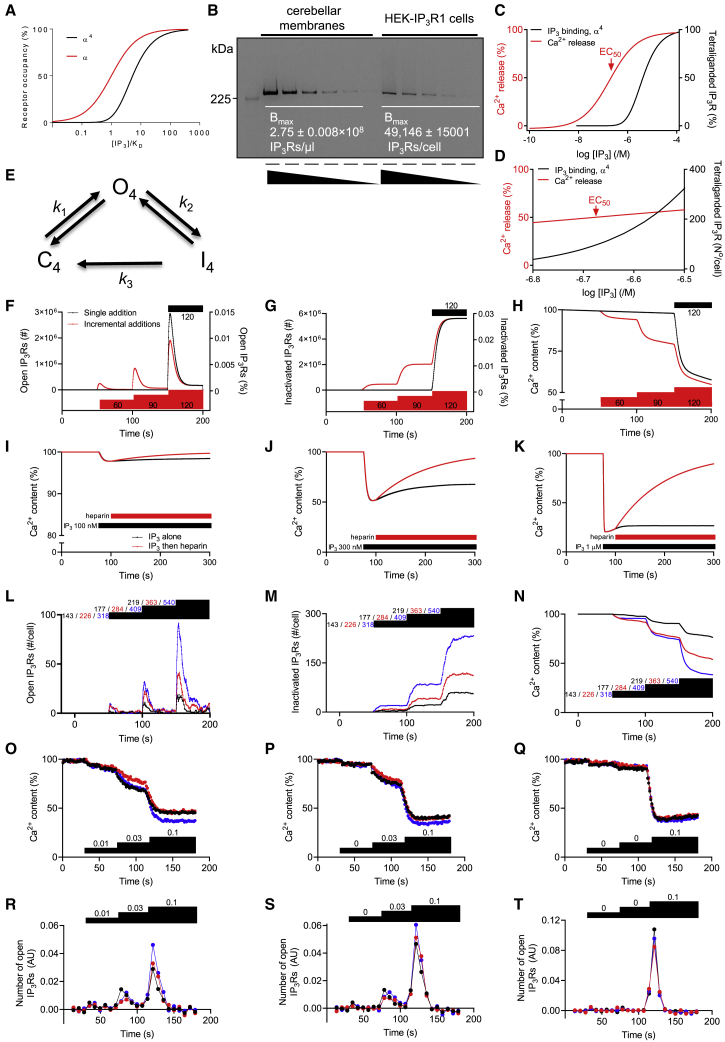
Incremental responses are mediated by very few IP_3_Rs that rapidly inactivate (A) IP_3_ must bind to all four IP_3_-binding sites of a tetrameric IP_3_R for the channel to open (Alzayady et al., 2016). Since IP_3_ binds independently to each site and with the same K_D_, we compute the fractional occupancy of all IP_3_-binding sites (α) at any ligand concentration ([L]) from: $\alpha =\left(\left[\text{L}\right]/{\text{K}}_{\text{D}}/1+\left[\text{L}\right]/{\text{K}}_{\text{D}}\right)$. The fraction of tetrameric IP_3_R in which all four sites are occupied is then α^4^. The plot shows the relationship between normalized ligand concentration ([L]/K_D_) and the fraction of IP_3_Rs in which all four IP_3_-bindings sites are occupied (α^4^) compared to the fractional occupancy at a single site (α). (B) Equilibrium competition binding with [^3^H]IP_3_ in TEM was used to define the K_D_ and from that the maximal number of binding sites (B_max_) in cerebellar membranes, which were then used to calibrate western blots to estimate the number of IP_3_Rs in a HEK-IP_3_R1 cell. Loadings are for serial 2-fold dilutions from 3.2 to 0.1 μL/lane (cerebellum) and from 12.8 to 0.8 μL/lane (HEK-IP_3_R1 cells). The HEK-IP_3_R1 cell lysate contained 10,000 cells/μL. B_max_ values are mean ± SEM, n = 6. (C) Predicted relationship for HEK-IP_3_R1 cells between IP_3_ concentration, number of IP_3_Rs (% of total) with all four IP_3_-binding sites occupied (calculated from the K_D_ for IP_3_ determined under conditions used for functional assays, 794 nM), and IP_3_-evoked Ca^2+^ release (% of IP_3_-sensitive stores; EC_50_ = 186 nM, pEC_50_ = 6.73 ± 0.04; n = 17). (D) Relationships between IP_3_ concentrations around the EC_50_ value, Ca^2+^ release, and the number of tetra-liganded IP_3_Rs/cell. (E) We assume that IP_3_Rs in which four subunits have bound IP_3_ move between closed (C_4_), open (O_4_), and inactivated (I_4_) states with forward rate constants *k*_1_ > *k*_2_ >> *k*_3_. (F–H) Simulations show numbers of open (F) and inactivated IP_3_Rs/well (G), and the ER Ca^2+^ content (H) for HEK-IP_3_R1 cells after sequential additions to give the indicated final IP_3_ concentrations (nM) or a single addition of the final IP_3_ concentration. Each addition of IP_3_ evokes a rapid, transient surge in the number of open IP_3_Rs, which then rapidly inactivate, leading to transient Ca^2+^ release. Note the tiny fraction of open and inactivated IP_3_Rs (right axes in F and G). (I–K) Simulations for HEK-IP_3_R1 cells with SERCAs active show ER Ca^2+^ content after addition of the indicated IP_3_ concentrations and then heparin (similar to analyses in Figures 5 and 6). Addition of heparin was simulated by making *k*_*rel*_ = 0. (L–N) In the simulations of cell populations (F–H), stepwise increases in IP_3_ concentration caused the predicted number of tetra-liganded IP_3_Rs (IP_3_R^4^) to increase by 4.41-fold (for 60 to 90 nM) and then by 2.76-fold (90 to 120 nM). Here, we simulate responses from a single wild-type HEK cell expressing 9,101 IP_3_Rs, with the first IP_3_ addition triggering formation of 5 IP_3_R^4^s/cell (143 nM), 10 IP_3_R^4^s/cell (177 nM), or 20 IP_3_R^4^s/cell (219 nM). We then deliver two further IP_3_ additions calculated to cause the numbers of IP_3_R^4^s to increase by 4.41-fold and then by 2.76-fold to mimic the stimuli used in simulations of larger numbers of cells (F–G). Simulations show the effects of adding the indicated concentrations of IP_3_ on the number of open (L) and inactivated IP_3_Rs (M), and the Ca^2+^ content of the ER (N). Colored code indicates concentrations of IP_3_ (nM) added at each step. (O–T) We predict the number of open IP_3_Rs during incremental responses of permeabilized DT40-IP_3_R1 cells by subtracting the ER Ca^2+^ leak after inhibition of SERCA (Figure 1D, purple trace) from the results obtained with incremental additions of IP_3_ (Figures 1G–1H), and then estimate the time-dependent loss of ER Ca^2+^ due to opening of IP_3_Rs (see *Analysis* in STAR Methods). For each sequence of IP_3_ additions, results are shown for each of three independent analyses (blue, red, and black). The results show that after each IP_3_ addition, IP_3_Rs abruptly open and then close (inactivate), and the time course of the number of open IP_3_Rs matches predictions of the simple activation-inactivation scheme (F–H), confirming that our scheme captures essential features of IP_3_Rs responding incrementally to IP_3_.

Analyses of [^3^H]IP_3_ binding establish the fraction of IP_3_-binding sites to which each concentration of IP_3_ has bound (α) without revealing how the binding is distributed between IP_3_Rs or their different states. However, Hill coefficients for IP_3_ binding to IP_3_R1 are close to one (0.95 ± 0.08 in Figure 2B) (Hannaert-Merah et al., 1994; Iwai et al., 2005; Rossi et al., 2009; Supattapone et al., 1988), indicating that each IP_3_R subunit binds IP_3_ independently (Hannaert-Merah et al., 1994) and that differences in the affinities of different IP_3_R states are either small or masked by the predominance of a single state. We therefore assume that IP_3_ binding is independently distributed across all available IP_3_-binding sites. Since IP_3_R opening requires binding of IP_3_ to all four IP_3_R subunits (Alzayady et al., 2016), we need to predict the relationship between IP_3_ concentration and the fraction of IP_3_R with all four sites occupied (α^4^, Figure 7A). We begin with analyses of HEK-IP_3_R1 cells.

Our measurements of IP_3_-evoked Ca^2+^ release lack the temporal resolution needed to determine the initial rates of Ca^2+^ release that would directly report the number of open IP_3_Rs (Marchant and Taylor, 1998). However, the assays do establish the IP_3_ sensitivity of quantal responses, because (with SERCAs inhibited) the Ca^2+^ release determined after 20 s for each IP_3_ concentration reports the cumulative response to IP_3_ throughout that interval. When addressing mechanisms of quantal Ca^2+^ release, it is, therefore, appropriate to compare the sensitivity to IP_3_ of quantal Ca^2+^ release (the fraction of Ca^2+^ stores released) to estimates of how many IP_3_Rs are likely to be tetra-liganded by that concentration of IP_3_. We used IP_3_R1 from cerebellum (2.75 × 10^8^ IP_3_Rs/μL of sample, determined from [^3^H]IP_3_ binding) to calibrate western blots, and so determine the number of IP_3_Rs in HEK-IP_3_R1 cells (49,146 ± 15,001 IP_3_Rs/cell) (Figure 7B). Comparing the affinity of IP_3_R1 for IP_3_ under conditions that replicate functional assays (Figure 2G) with the number of IP_3_Rs in HEK-IP_3_R1 cells, we predict that the Ca^2+^ release evoked by a half-maximally effective concentration of IP_3_ is associated with opening of only 0.13% of a cell’s IP_3_Rs, or about 60 IP_3_Rs/cell (Figures 7C and 7D). This situation, where only a tiny fraction of available receptors elicits a response, is analogous to the “spare receptors” found in many signaling pathways initiated by plasma membrane receptors. Here, all receptors are competent to respond, but there are many more receptors than required to elicit a maximal response (Stephenson, 1956). Our argument does not require an additional level of IP_3_R regulation, although that may also occur (Thillaiappan et al., 2017; Thillaiappan et al., 2021); it requires only that cells express more IP_3_Rs than are needed to deplete the ER of Ca^2+^. In analogy with spare receptors elsewhere, we expect this feature to increase the sensitivity of Ca^2+^ release to IP_3_.

An open IP_3_R is predicted to mediate movement of about 500,000 Ca^2+^/s out of the ER (Vais et al., 2010). We estimate the ER Ca^2+^ content of a single permeabilized HEK cell to be about 9 × 10^−16^ mol (see *Simulations* in STAR Methods). This suggests that sustained opening of a single IP_3_R for the 20 s taken for incremental responses to plateau would be sufficient to cause loss of almost 2% of the ER Ca^2+^ content of a cell. The key point is that very few of the many IP_3_Rs in a cell are active during incremental responses to IP_3_ and they are sufficient to account for the observed responses (Figure 7D).

### A mechanism for incremental responses to IP_3_

We propose that after IP_3_ addition, very few of the many IP_3_Rs in a cell rapidly bind IP_3_ to each of their four subunits; many more IP_3_Rs will be partially occupied (Figures 7A, 7C, and 7D). The fully occupied IP_3_Rs open and rapidly release Ca^2+^; they then inactivate and more slowly recover (Figure 7E). After the initial Ca^2+^ release, when activation of IP_3_Rs is effectively synchronized by IP_3_ addition, additional IP_3_Rs will open as they bind four IP_3_ molecules and then proceed to inactivate, but their openings will be asynchronous. Hence, addition of IP_3_ drives a few IP_3_Rs through a near-synchronous sequence of opening and inactivation, after which very small numbers of IP_3_Rs proceed through the sequence asynchronously. The next step in IP_3_ concentration then drives another pulse of near-synchronous IP_3_R openings.

Two features of this scheme contribute to understanding incremental responses. First, incremental responses are mediated by only a tiny fraction of the IP_3_Rs in a cell (<1%, Figures 7C and 7D). Inactivation therefore negligibly impacts the number of IP_3_Rs available for subsequent activation, ensuring that despite some inactivation of IP_3_Rs, responses to subsequent IP_3_ additions are unperturbed. Second, because IP_3_Rs move inexorably from an open to an inactivated state, there will always be more open IP_3_Rs immediately after an IP_3_ addition (when openings are synchronized) than later (when IP_3_Rs progress to inactivated states) (Figure 7E). A surge of Ca^2+^ release after each IP_3_ addition is followed by a period of much reduced activity, thereby explaining the quantal pattern of Ca^2+^ release.

There is evidence, albeit with conflicting observations, that rapid activation of IP_3_Rs by IP_3_ is followed by slower inactivation (half-time [t_½_] of about 15–30 s) (Hajnóczky and Thomas, 1994; Mak et al., 2000, 2001; Stehno-Bittel et al., 1995) and even slower recovery (t_½_ of 30 s to several minutes) (Hajnóczky and Thomas, 1994; Ionescu et al., 2006). This mechanism would allow incremental responses only if inactivation requires most (probably all four) IP_3_-binding sites to be occupied; otherwise, IP_3_Rs would inactivate before they opened. An alternative mechanism is “feed-through” inhibition of IP_3_Rs by Ca^2+^ as it passes through an open channel (Dawson et al., 2003), but even these very local Ca^2+^ signals would probably be intercepted by 10 mM BAPTA (Vais et al., 2012), which did not prevent quantal responses (Figures S2A–S2F). We suggest that IP_3_R inactivation is directly linked to activation rather than via feedback effects from increased [Ca^2+^]_c_.

### A simple activation-inactivation scheme predicts incremental responses to IP_3_

We developed an empirical scheme with stochastic simulations to assess whether it captured key features of the experimental observations. A simplified scheme is needed to reduce computation times for the stochastic simulations of the many IP_3_Rs required to address the consequences of a few IP_3_Rs opening together and then losing synchrony as they inactivate. The scheme envisages that an IP_3_R opens rapidly (t_½_ of ∼2.5 s) after it has bound four molecules of IP_3_, before slowly inactivating (t_½_ of ∼5 s) and then very slowly recovering from the inactivated to the closed state (t_½_ of ∼250 s) (Figure 7E). The detailed mechanisms of IP_3_-evoked IP_3_R inactivation are unresolved and may require interaction with additional unidentified molecules. Hence, our empirical scheme based on the activation-inactivation sequence shown in Figure 7E is not a closed cycle (see *Simulations* in STAR Methods).

Mathematical simulations using our simplified scheme (Figure 7E) and with the numbers of IP_3_Rs determined in functional analyses of HEK-IP_3_R1 cells in 96-well plates (4 × 10^5^ cells/well, ∼2 × 10^10^ IP_3_Rs/well) (Figure 7B) capture the key experimental observations. Each addition of a submaximal IP_3_ concentration evokes a surge of IP_3_R openings followed by stable inactivation of a small fraction of the IP_3_R population. The ER Ca^2+^ content responds incrementally to successive IP_3_ additions. Finally, with SERCAs active, stores rapidly refill in the presence of low IP_3_ concentrations, but remain depleted with higher concentrations (Figures 7F–7K).

Most of our analyses used populations of cells in 96-well plates, but incremental responses to IP_3_ are also evident in single wild-type HEK cells (Figures S4J–S4N). We therefore measured the number of IP_3_Rs in wild-type HEK cells (9,101 ± 934 IP_3_Rs/cell, mean ± SD, n = 3) and applied our scheme to a single cell. The simulations confirm that here too, the scheme predicts behavior consistent with experimental observations. Incremental additions of IP_3_ evoke graded loss of ER Ca^2+^, which is associated with transient opening of very small numbers of IP_3_Rs and sustained inactivation of a few IP_3_Rs (Figures 7L–7N).

We also compared the activation-inactivation scheme with experimental data from DT40-IP_3_R1 cells responding incrementally to IP_3_ (Figures 1G–1J). Using the experimental data, we identified the component of ER Ca^2+^ release attributable to open IP_3_Rs by subtracting the basal IP_3_-independent Ca^2+^ leak (see *Analysis* in STAR Methods). By dividing this IP_3_R-dependent Ca^2+^ flux by the time-matched ER Ca^2+^ content, we estimated the time course of the number of open IP_3_Rs (in arbitrary numbers) during sequential additions of IP_3_ (Figures 7O–7T). The analysis establishes that IP_3_Rs must close within about 10 s of each IP_3_ addition. Furthermore, the close correspondence of the time course of IP_3_R openings in the experimental analysis with the predictions of our simple, albeit incomplete, activation-inactivation scheme (Figure 7E) confirms that the scheme captures all essential features of the experimental observations.

### Conclusions

Quantal Ca^2+^ release is an unusual feature of IP_3_Rs, first described in 1989 (Muallem et al., 1989), but never adequately explained (Yamashita, 2006). We confirmed that in both intact and permeabilized cells and under conditions where SERCAs cannot counteract IP_3_R activity, submaximal concentrations of IP_3_ rapidly release only a fraction of the ER Ca^2+^ stores without compromising responses to further incremental IP_3_ additions. These features are properties of all three IP_3_R subtypes; they are evident in single cells; they do not arise from ineffective charge compensation during electrogenic Ca^2+^ efflux, IP_3_ metabolism, increases in [Ca^2+^]_c_, or mitochondrial activity; and they are a property of IP_3_Rs rather than the ER.

We developed a synthetic high-affinity agonist of IP_3_Rs with very low efficacy, **2**, to distinguish between the mechanisms proposed to explain quantal Ca^2+^ release (Figures 1A and 1B); **2** has the lowest efficacy reported for any high-affinity agonist of IP_3_R. We used **2** to show that quantal responses do not arise from all-or-nothing emptying of heterogenous Ca^2+^ stores. We do not exclude the possibility that IP_3_Rs within a cell may differ in their IP_3_ sensitivity, but it is clear that any such heterogeneity is not required for quantal responses (Figure 3). Instead, after the initial rapid Ca^2+^ release evoked by low concentrations of IP_3_, IP_3_Rs close with Ca^2+^ still trapped in the ER. Neither erlin 2, an increase in [Ca^2+^]_c_, nor a decrease in ER luminal [Ca^2+^] contributes to this IP_3_R inactivation. Instead, inactivation is probably an inexorable consequence of IP_3_R activation (Hajnóczky and Thomas, 1994), although the detailed mechanisms are unresolved.

Comparison of receptor occupancy and functional responses reveals that only a tiny fraction of a cell’s IP_3_Rs contribute to incremental responses. These analyses, supported by simulations (Figures 7E–7T), reconcile incremental responses with IP_3_R inactivation. A surge of coordinated opening of IP_3_Rs occurs with each step in IP_3_ concentration. This is followed by IP_3_R inactivation. Thereafter, there will never be the same balance in favor of the open state until the next step in IP_3_ concentration triggers another coordinated sequence of IP_3_R opening and then inactivation (Figures 7F and 7L). Pulsatile delivery of IP_3_ to intact cells may be provided by changes in extracellular stimulus intensity or by cycles of IP_3_ production and degradation driven by feedback regulation of phospholipase C in the sustained presence of an extracellular stimulus (Bartlett et al., 2015; Dupont et al., 2011; Meyer and Stryer, 1988; Nash et al., 2001).

Our results suggest a mechanism for incremental responses to IP_3_, a phenomenon that has remained unresolved for more than 30 years. Unrestrained IP_3_Rs might evoke explosive responses through CICR. The mechanism we propose allows cells to evoke rapid Ca^2+^ signals through IP_3_Rs that are graded with stimulus intensity (Figure 1P).

### Limitations of the study

Two essential features underpin our explanation for incremental responses to IP_3_: rapid inactivation of IP_3_Rs after their opening, and physiological responses mediated by a tiny fraction of available IP_3_Rs. We do not yet understand the molecular mechanisms underlying IP_3_R inactivation, and notably whether accessory proteins are required. Understanding these mechanisms would allow us to refine our simplified scheme, which presently includes an irreversible step that forbids transitions from the closed (C_4_) to inactivated (I_4_) state of IP_3_Rs. Hence, our simplified scheme is not a closed cycle, suggesting that it is coupled to an additional mechanism, perhaps reflecting the need for additional proteins to mediate inactivation. High-resolution optical microscopy reveals that low concentrations of IP_3_ evoke small, highly localized Ca^2+^ signals (“Ca^2+^ puffs”) in intact cells neighbors (Smith and Parker, 2009; Thillaiappan et al., 2021). It is not yet clear how incremental responses to IP_3_ relate to Ca^2+^ puffs.

## STAR★Methods

### Key resources table

**Table:** 

REAGENT or RESOURCE	SOURCE	IDENTIFIER
**Antibodies**		

Anti-IP_3_R Ab (AbC, recognizes all vertebrate IP_3_R subtypes, rabbit, monoclonal) (1:1000)	Cell Signaling Technology (Danvers, MA, USA)	Cat#8568S
		Clone # D53A5
		RRID: AB_10890699
Anti-β-actin Ab (mouse monoclonal) (1:30000)	Cell Signaling Technology	Cat#3700S
		Clone # 8H10D10
		RRID: AB_2242334
Anti-erlin 2 Ab (rabbit, monoclonal) (1:1000)	Abcam (Cambridge, UK)	Cat#ab129207
		Clone # EPR8088
		RRID:AB_11143745
Anti-mouse secondary Ab (mouse IgG κ-binding protein conjugated to horseradish peroxidise, HRP) (1:5000)	Santa Cruz (Dallas, TX, USA)	Cat#sc-516102
		RRID: AB_2687626
Anti-rabbit secondary Ab (goat monoclonal, conjugated to HRP) (1:5000)	SeraCare (Milford, MA, USA)	Cat#5220-0336
		RRID: AB_2857917
**Chemicals, peptides, and recombinant proteins**		

2-aminoethoxy diphenyl borate (2-APB)	BioVision (Milpitas, CA, USA)	Cat#1798-100
ATP	Sigma-Aldrich (St. Louis, MO, USA)	Cat#A6419
Bafilomycin A_1_	Fluorochem (Hadfield, UK).	Cat#M01404
BAPTA	Phion (Dorset, UK)	Cat#61061782
Bis(4-nitrophenyl) carbonate	Acros Organics via ThermoFisher (Waltham, MA, USA)	Cat#171600250
Cbz_3_-spermine-IP_3_ (**2**)	Synthesis and properties described in this paper	Figure 2A
Chelex-100 resin Na^+^ form	Sigma-Aldrich	Cat#C7901
Cyclopiazonic acid (CPA)	Tocris (Bristol, UK)	Cat#1235
Dequalinium chloride hydrate	Sigma-Aldrich	Cat#D3768
d-*myo*-inositol 1,4,5-trisphosphate (IP_3_)	Enzo (Exeter, UK)	Cat#ALX-307-007-M005
d-*myo*-[^3^H]inositol 1,4,5-trisphosphate ([^3^H]IP_3_) (19.3-21 Ci/mmol)	PerkinElmer (Beaconsfield, UK)	Cat#NET911005UC
d-*myo-*inositol 2,4,5-trisphosphate ((2,4,5)IP_3_)	Cayman Chemical (Ann Arbor, MI, USA)	Cat#10007779
Dulbecco’s modified Eagle’s medium/nutrient mixture F-12 with GlutaMAX (DMEM/F-12 GlutaMAX)	ThermoFisher	***Cat#10565018***
Foetal bovine serum (FBS)	Sigma	Cat#F7524
G418	Formedium (Norfolk, UK)	Cat#G4181
Heparin, sodium salt	Calbiochem via Merck (Watford, UK)	Cat#375095
Ionomycin, Ca^2+^ salt	Apollo Scientific (Stockport, UK)	Cat#BII0123
LiChroprep RP-18 (25-40 μm)	Merck	Cat#109303
Mag-fluo 4 AM	ThermoFisher	Cat#M14206
ML-SA1	Merck	Cat#648493
Poly-l-lysine (1% solution)	Sigma	Cat#P8920
Q-Sepharose Fast Flow resin	GE Healthcare (Chicago, IL, USA)	Cat#GE17-0510-01
Saponin	Sigma-Aldrich	Cat#S4521
Thapsigargin	Bio-Techne (Middlesex, UK)	Cat#1138/1
Triethylamine	Sigma-Aldrich	Cat#90340

**Critical commercial assays**		

8-well glass-bottom μ-slides	Ibidi (Munich, Germany)	Cat#80827
35-mm glass bottom dishes (#1 glass)	Cellvis (Mountain View, CA 94039)	Cat#D35-14-1-N
ECL-Prime/Select western blotting reagents	Amersham via GE Healthcare	Cat#RPN2236/RPN2235
Half-area 96-well black-walled plates	Greiner, Bio-One (Stonehouse, UK)	Cat#675090
Neon transfection system kit	ThermoFisher	Cat#MPK10096
iBlot gel-transfer stacks PVDF	ThermoFisher	Cat#IB401001
Protease inhibitor cocktail (Roche)	Merck	Cat#11836153001
NuPAGE, SDS-PAGE gels (3-8% Tris-acetate or 4-12% Bis-Tris)	ThermoFisher	Cat#EA0375BOX or NP0321BOX
TransIT-LT1 transfection reagent	Geneflow (Lichfield, UK)	Cat#MIR 2305

**Experimental models: Cell lines**		

*Gallus gallus* DT40-IP_3_R1 (DT40-IP_3_R2; DT40-IP_3_R3) cells	Tovey et al., 2006	N/A
*Homo sapiens* HEK-3KO cells	Kerafast (Boston, MA, USA) (Alzayady et al., 2016)	Cat#EUR030
*Homo sapiens* HEK-G-CEPIA*er* cells	This paper	N/A
*Homo sapiens* wild-type HEK293 cells	Dr D Yule, University of Rochester, NY, USA	N/A

**Oligonucleotides**		

siRNA targeting sequence of Erlin 2 (siRNA 1) ATCTACTTTGACAGAATTGAA	QIAGEN (Manchester, UK)	Cat#SI04952689
siRNA targeting sequence of Erlin 2 (siRNA 2) AAGATAGAAGAGGGACATATT	QIAGEN	Cat#SI04952696
Non-silencing (NS) siRNA	QIAGEN	Cat#1027280

**Recombinant DNA**		

CFP-TRPML1 plasmid	Addgene (Venkatachalam et al., 2006)	Cat#18827
G-CEPIA1*er* plasmid	Addgene (Suzuki et al., 2016)	Cat#58215
IP_3_R1 in pcDNA3.1(-) /Myc-His B plasmid	Dohle et al., 2019	N/A
EGFP-IP_3_R1 in pcDNA3.2/DEST	Pantazaka and Taylor, 2011	N/A
Residues 1-604 of rat IP_3_R1 (NT) in pTrcHisA plasmid	Rossi et al., 2009	N/A
Residues 224-604 of rat IP_3_R1 (IBC) in pTrcHisA plasmid	Rossi et al., 2009	N/A

**Software and algorithms**		

Fiji		Schindelin et al., 2012
MetaMorph. Version 7.10.1.161	Molecular Devices (San Jose, CA, USA)	N/A
PRISM. Version 8.4.2	GraphPad Software (La Jolla, CA, USA)	N/A
QuB	Qin, 2004	N/A
SoftMax Pro. Version 5.4	Molecular Devices	N/A
Codes for simulations	This paper	Available from: https://github.com/genedupont/Quantal

### Resource availability

#### Lead contact

Requests for resources and reagents should be directed to the Lead Contact, Colin W. Taylor (cwt1000@cam.ac.uk).

#### Materials availability

All plasmids used are commercially available, except EGFP-IP_3_R1 which is available from the Lead Contact. The cell lines generated in this study are available from the Lead Contact. Compound **2** is available from Barry V. L. Potter (barry.potter@pharm.ox.ac.uk).

### Experimental model and subject details

#### Cells

DT40 cells are an avian B cell line, wherein high rates of DNA recombination have been widely exploited for analyses of cell signaling and B cell function (Winding and Berchtold, 2001). DT40 cells were the first cells in which all three IP_3_R subtypes were genetically disrupted to provide a null-background for IP_3_R expression (Sugawara et al., 1997). We used DT40 cells without native IP_3_Rs to generate cell lines stably expressing single subtypes of mammalian IP_3_R (DT40-IP_3_R1-3) (Tovey et al., 2006).

Human embryonic kidney 293 (HEK293 cells) are hypotriploid cells derived from embryonic kidney and immortalized by transformation with adenovirus (Shaw et al., 2002). We used wild-type HEK cells (which express all three IP_3_R subtypes) (Mataragka and Taylor, 2018), and a stable cell line in which CRISPR/Cas9 was used to prevent expression of all native IP_3_Rs (HEK-3KO) (Alzayady et al., 2016). HEK-3KO cells were transfected with mammalian IP_3_R1 to generate a monoclonal HEK-IP_3_R1 cell line, and wild-type HEK cells were used to generate HEK-G-CEPIA1*er* cells stably expressing the ER-targeted genetically-encoded Ca^2+^ indicator, G-CEPIA1*er*.

The identity of the parental HEK293 cell line used to generate HEK-3KO cells was confirmed by genotyping (Alzayady et al., 2016), and we confirmed the absence of all IP_3_Rs by western blotting (Mataragka and Taylor, 2018). None of the other cell lines was authenticated. All cells were periodically (every 2 months) confirmed to be free of mycoplasma.

### Method details

#### Synthesis of 2

We originally synthesized **2** (2-*O*-(1,5,10-tris(benzyloxycarbonyl)-15-oxo-1,5,10,14,16-pentaazaoctadecan-18-yl)-1D-*myo*-inositol 1,4,5-trisphosphate, Figure 2A) as an intermediate in the synthesis of a novel polyamine-IP_3_ conjugate, but **2** proved to be most useful for our present purposes. Bis(4-nitrophenyl) carbonate was recrystallized from CH_2_Cl_2_/hexane. d-2-*O*-(2-aminoethyl)-IP_3_ was synthesized as previously reported (Riley et al., 2004) and used as the triethylammonium salt. *N*^1^,*N*^5^,*N*^10^-tri(benzyloxycarbonyl)spermine was synthesized according to (Blagbrough and Geall, 1998). Triethylammonium bicarbonate (TEAB) buffer was made by bubbling CO_2_ through a chilled aqueous solution of triethylamine. Anion-exchange chromatography was carried out on Q-Sepharose Fast Flow resin (GE Healthcare) using a Pharmacia Biotech Gradifrac system and P-1 pump, eluting at 5 mL/min with a linear gradient (0 to 100%) of 2.0 M aqueous TEAB buffer, collecting 10-mL fractions. Phosphate-containing fractions were identified using a modification of the Briggs phosphate test (Lampe et al., 1994). Reverse-phase ion-pair chromatography was carried out on LiChroprep RP-18 (25-40 *μ*m, Merck) using a BioLogic LP system (BioRad), eluting at 5 mL/min with a linear gradient (0 to 70%) of CH_3_CN in 0.05 M TEAB buffer, collecting 7-mL fractions. Compound **2** was accurately quantified using the Ames phosphate assay (Ames and Dubin, 1960). All water used was MilliQ quality.

To a solution of bis(4-nitrophenyl) carbonate (200 mg, 0.66 mmol) in dry CH_2_Cl_2_ (5 mL) under N_2_ was added a solution of *N*^1^,*N*^5^,*N*^10^-tri(benzyloxycarbonyl)spermine (400 mg, 0.66 mmol) in dry CH_2_Cl_2_, dropwise over 10 min. The solution was stirred for 1 hr, after which TLC (CH_2_Cl_2_/MeOH 10:1 v/v) showed that all the amine (streak, *R*_f_ 0.06) had been consumed, with appearance of a product at *R*_f_ 0.72 (yellow on heating and stains with phosphomolybdic acid), together with 4-nitrophenol (*R*_f_ 0.5) and a trace of unreacted bis(4-nitrophenyl) carbonate (*R*_f_ 0.86). CH_2_Cl_2_ (20 mL) was added, and the solution was washed with saturated aqueous NaHCO_3_ (3 × 20 mL). The pale yellow organic layer was dried (MgSO_4_) and concentrated to give a yellow oil, which was purified by flash chromatography (CH_2_Cl_2_, then CH_2_Cl_2_:MeOH, 50:1 v/v) to give 4-nitrophenyl *N*-alkylcarbamate (436 mg) as a colorless oil; TLC *R*_f_ 0.24 (CH_2_Cl_2_/EtOAc, 5:1). A solution of this alkylcarbamate (46 mg, 60 *μ*mol) in CD_3_OD (0.75 mL) was added to solid 2-*O*-(2-aminoethyl)-IP_3_ (triethylammonium salt, 21 mg, 28 *μ*mol) under N_2_ in a 5-mL round-bottom flask, followed by dry triethylamine (40 *μ*L). A deep yellow color (4-nitrophenol) appeared within seconds. The clear yellow solution was stirred at room temperature for 2 hr, then transferred to an NMR tube. The ^31^P NMR spectrum of this solution showed three major peaks (1.01, 3.17 and 4.18 ppm) corresponding to **2**, together with other, smaller peaks. The solution was left for 16 hr in the NMR tube, after which a second ^31^P NMR spectrum showed only the three product peaks. The reaction was therefore judged to be complete. The contents of the NMR tube were transferred to a 100-mL round-bottom flask and the NMR tube was rinsed with MeOH (1 mL). To the combined washings was added triethylammonium bicarbonate (TEAB) buffer (0.05 M, pH 7.6, 75 mL), and the resulting cloudy yellow solution was left for 16 hr at room temperature to hydrolyse any unreacted *N*-alkylcarbamate. The solution was then loaded onto a column of Q-Sepharose Fast Flow resin (120 mm × 16 mm, bicarbonate form). The column was washed well with water (200 mL) and then eluted with a gradient of TEAB (2.0 M, 0 to 100%). At approx. 40% 2.0 M TEAB, the column eluent took on an intense yellow color (4-nitrophenol) and this buffer concentration was held constant until the eluent became colorless. When the concentration of TEAB was then allowed to increase, the target compound eluted above 90% 2.0 M TEAB. Fractions containing the target compound were identified using the Briggs phosphate assay, combined and concentrated under reduced pressure to give a colorless glassy residue (40 mg). ^1^H NMR spectroscopy of the product at this stage showed that it contained significant amounts of alkylammonium contaminants, resulting from the high concentration of TEAB buffer needed to elute it from the column. It was therefore purified further using reverse-phase ion-pair chromatography: the 40 mg of material was taken up in 0.05 M TEAB (5 mL) and loaded onto a column of LiChroprep RP-18 resin (100 mm × 16 mm). The column was washed well with 0.05 M TEAB, and then eluted with a gradient of CH_3_CN in 0.05 M TEAB (0 to 70% CH_3_CN). The target eluted at 33%–40% CH_3_CN, as detected by UV absorption at 254 nm. Fractions containing the target compound were combined and concentrated under reduced pressure. MeOH was repeatedly added and evaporated until a colorless glass remained (∼35 mg). Finally, this material was dissolved in water (5 mL) and passed through a column of Chelex-100 resin (Na^+^ form, 70 mm deep in a Pasteur pipette) to remove triethylammonium ions. The column was washed with water (5 mL) and the combined eluents were lyophilised to give **2** as a white, fluffy solid (28 mg, 19.7 *μ*mol, 70% as determined by total phosphate assay); ^1^H NMR (Na^+^ salt in D_2_O, 400 MHz) δ 1.16–1.48 (4H, broad m, 2 × spermine CH_2_), 1.49–1.80 (4H, broad m, 2 × spermine CH_2_), 2.90–3.32 (12H, broad m, 6 × spermine CH_2_), 3.39 (2H, broad s, NC*H*_2_CH_2_O), 3.80 (1H, broad d, *J* 9.9 Hz, inositol H-3), 3.87–4.13 (5H, m, inositol H-1, H-5. H-6 and NCH_2_C*H*_2_O), 4.15 (1H, broad s, inositol H-2), 4.33 (1H, q, *J* 9.1 Hz, inositol H-4), 4.92–5.14 (6H, m, 3 × OC*H*_2_Ph), 7.14–7.45 (15H, m, Ph); ^13^C NMR (Na^+^ salt in D_2_O, 126 MHz) δ 24.42, 29.96, 27.35 and 28.02 (spermine CH_2_), 37.40 and 38.01 (spermine CH_2_), 40.20 (N*C*H_2_CH_2_O), 44.04, 45.01 and 46.40 (spermine CH_2_), 66.51 and 67.20 (3 × O*C*H_2_Ph), 71.38 (inositol C-3), 71.91 (inositol C-6), 72.72 (NCH_2_*C*H_2_O), 74.59 (inositol C-1), 76.46 (inositol C-4), 78.31 (inositol C-5), 79.69 (inositol C-2), 127.53–128.58 (CH of Ph), 136.27 and 136.60 (*ipso*-C of Ph), 157.21, 157.37 and 157.82 (3 × urethane C = O), 160.56 (urea C = O); ^31^P NMR (triethylammonium salt in CD_3_OD, 162 MHz, ^1^H-decoupled) δ 0.81 (1 P), 1.55 (1 P) and 2.50 (1 P); HRMS (*m*/*z*) [M–H]^–^ calcd for C_43_H_62_N_5_O_22_P_3_, 1092.3027; found 1092.3017.

#### Cell culture and transfections

Methods used to generate and culture DT40 cells without native IP_3_Rs and then transfected to stably express a single subtype of mammalian IP_3_R (DT40-IP_3_R1-3) were described previously (Tovey et al., 2006). HEK293 cells were cultured in DMEM/F-12 GlutaMAX medium with 10% FBS at 37°C in 95% air and 5% CO_2_. Cells were passaged or used for experiments when they reached confluence. Plasmids were transfected into HEK cells using TransIT-LT1 reagent. The transfection efficiency of HEK cells transiently expressing CFP-TRPML1 or EGFP-IP_3_R1 was ∼60%, assessed by CFP or EGFP fluorescence. For double transfections (CFP-TRPML1 and EGFP-IP_3_R1), the transfection efficiency was ∼15%. To generate HEK cells expressing only IP_3_R1 (HEK-IP_3_R1 cells), HEK-3KO cells were transfected with the gene encoding rat IP_3_R1 (lacking the S1 splice site) (Rossi et al., 2009) cloned into pcDNA3.1(-)/Myc-His B plasmid (Dohle et al., 2019). To produce HEK cells stably expressing G-CEPIA1*er* (HEK-G-CEPIA1*er* cells), wild-type HEK cells were transfected with G-CEPIA1*er* plasmid (Suzuki et al., 2014). To generate stable cell lines (HEK-IP_3_R1 and HEK-G-CEPIA1*er* cells), cells were passaged 48 hr after transfection in medium with G418 (1 mg/mL). Selection was maintained for 2 weeks with medium changes every 3 days. Monoclonal HEK-IP_3_R1 cell lines were selected by plating cells (∼1 cell/well) into 96-well plates in medium containing G418 (1 mg/mL). After 4 days, wells with only one cell were identified, cells were then grown to confluence, and cell lines were expanded and their expression of IP_3_R1 was confirmed by western blot using an antibody specific for IP_3_R1 (Rossi et al., 2009). To obtain polyclonal HEK-G-CEPIA1*er* cells, cells with the highest G-CEPIA1*er* expression (∼1%) were isolated using fluorescence-activated cell sorting (FACS). siRNA transfections of wild-type HEK cells used a Neon transfection system with a final siRNA concentration of 150 nM. Cells were incubated for 48 hr before use. The siRNAs used are listed in the Key Resources table.

#### Ca^2+^ release by IP_3_Rs

Incubation of cells with the acetoxymethylester (AM) of Mag-fluo 4 traps low-affinity forms of the indicator (${\text{K}}_{\text{d}}^{\text{Ca}}$ = 1.15 mM measured *in situ*) within the ER. Fluorescence, which is approximately linearly related to ER free [Ca^2+^] (since [Ca^2+^]_ER_ < ${\text{K}}_{\text{d}}^{\text{Ca}}$), can then be used, without further calibration, to reliably report free [Ca^2+^] within the ER lumen (Rossi and Taylor, 2020). The ER of DT40 or HEK293 cells was loaded with indicator by incubating cells with Mag-fluo 4 AM (20 μM, 60 min, 22°C) in HEPES-buffered saline (HBS: 135 mM NaCl, 5.9 mM KCl, 11.6 mM HEPES, 1.5 mM CaCl_2_, 11.5 mM glucose, 1.2 mM MgCl_2_, pH 7.3) as described (Rossi et al., 2009). After washing and permeabilization with saponin (10 μg/mL, 37°C, 2-3 min) in Ca^2+^-free cytosol-like medium (Ca^2+^-free CLM), cells were centrifuged (650 × *g*, 2 min) and resuspended in Mg^2+^-free CLM supplemented with CaCl_2_ to give a final free [Ca^2+^] of 220 nM after addition of 1.5 mM MgATP. Ca^2+^-free CLM comprised: 20 mM NaCl, 140 mM KCl, 1 mM EGTA, 20 mM PIPES, 2 mM MgCl_2_, pH 7.0. Cells (4 × 10^5^ cells/well) were attached to poly-L-lysine-coated 96-well black-walled plates (Greiner Bio-One, Stonehouse, UK), and fluorescence (excitation and emission at 485 nm and 520 nm, respectively) was recorded at intervals of 1.44 s using a FlexStation III plate-reader (Molecular Devices, Sunnyvale, CA, USA). MgATP (1.5 mM) was added to initiate Ca^2+^ uptake, and when the ER had loaded to steady state with Ca^2+^ (∼150 s), ligands were added. Responses to the ligands show ER Ca^2+^ contents as either time courses or, in summary results, as the Ca^2+^ content recorded 20 s after addition of the stimulus. The distribution of samples across 96-well plates was varied between experiments to avoid any systematic ‘position-related’ artifacts. HEK-G-CEPIA1*er* cells were permeabilized and assayed as described for Mag-fluo 4-loaded cells.

#### Fluorescence microscopy

Fluorescence microscopy imaging was performed using an inverted Olympus IX83 microscope equipped with either a 60 × oil-immersion objective (numerical aperture, NA 1.45) or a 100 × oil-immersion total internal reflection fluorescence *(*TIRF) objective (NA 1.49), a multi-line laser bank (excitation light 425 nm and 488 nm) and an iLas2 targeted laser illumination system (Cairn, Faversham, Kent, UK). Excitation light was transmitted through either a quad dichroic beam splitter (TRF89902-QUAD) or a dichroic mirror (for 425 nm; ZT442rdc-UF2) (Chroma). Emitted light was passed through appropriate filters (Cairn Optospin; peak/bandwidth: 480/40 and 525/50) and detected using an iXon Ultra 897 electron-multiplied charge-coupled device (EMCCD) camera (512 × 512 pixels, Andor). Spinning-disc confocal microscopy used a spinning disc with a 70-μm pinhole (X-Light, Crest Optics). TIRFM used the iLas2 illumination system and the penetration depth was 90-140 nm. For analyses of the distribution of CFP-TRPML1 and EGFP-IP_3_R1 (Figure S5F), we confirmed that there was no bleed-through between channels. Image capture and processing used MetaMorph (Molecular Devices) and Fiji (Schindelin et al., 2012). All images were corrected for background (MetaMorph) by subtracting fluorescence detected from a region without cells.

#### Single-cell analyses of Ca^2+^ release

HEK-G-CEPIA1*er* cells were permeabilized with saponin, and resuspended in Mg^2+^-free CLM supplemented with CaCl_2_ to give a final free [Ca^2+^] of 220 nM after addition of 1.5 mM MgATP. Cells were attached (4 × 10^5^ cells/well) to a poly-L-lysine-coated 8-well glass bottomed μ-slide. MgATP (1.5 mM) was added to initiate Ca^2+^ uptake, and when the ER had loaded to steady state with Ca^2+^ (∼700 s), ligands were added.

Cells were imaged (2 s/frame) using spinning-disk confocal microscopy with a 60 × oil-immersion objective. Images were collected using MetaMorph, corrected for background fluorescence and analyzed using the Time Series Analyzer plugin (Fiji). Fluorescence intensity values from regions of interest (ROI) that delineated individual cells or ROI within individual cells are reported in arbitrary fluorescence units.

#### [^3^H]IP_3_ binding

IP_3_R1, which accounts for ∼99% of IP_3_Rs in cerebellum (Wojcikiewicz, 1995), was purified from cerebella of adult Wistar rats using heparin-affinity chromatography (Rossi et al., 2009). The purified protein (10 μg/mL) was stored at −80°C in 500 mM NaCl, 50 mM Tris, 10% glycerol, 1 mM 2-mercaptoethanol, 1 mM benzamidine, 1 mM EGTA, 50 mM Tris, 1% CHAPS, pH 8.0. N-terminal fragments of IP_3_R1 (IBC, residues 224-604; NT, residues 1-604) were expressed as N-terminally tagged His_6_-fusion proteins in *E. coli* and cleaved from the tag with thrombin (Rossi et al., 2009). Most equilibrium-competition binding assays (4°C, 5 min) were performed in Tris-EDTA medium (TEM, 500 μL) comprising 50 mM Tris, 1 mM EDTA, pH 8.3 with [^3^H]IP_3_ (21 Ci/mmol, 0.3-1.5 nM), bacterial lysate (7 μg protein) or purified IP_3_R1 (2 μg protein), and competing ligands (Rossi et al., 2009). Bound and free ligand were separated by centrifugation (4°C, 5 min, 14,000 *× g*). Non-specific binding, determined by addition of 10 μM IP_3_, was less than 10% of total binding.

For analyses of [^3^H]IP_3_ binding under conditions that mimicked those used for functional assays (Figure 2G), equilibrium-competition binding assays (22°C, 5-10 min) were performed in Mg^2+^-free CLM supplemented with CaCl_2_ to give a final free [Ca^2+^] of 220 nM, [^3^H]IP_3_ (19.3 Ci/mmol, 7.5 nM), membranes from cerebella of adult Wistar rats (∼100 μg protein) and competing ligands. Bound and free ligand were separated by centrifugation (22°C, 5 min, 14,000 *× g*). Non-specific binding, determined by addition of 100 μM IP_3_, was less than 10% of total binding.

#### Nuclear patch-clamp recording from IP_3_Rs

The methods used for patch-clamp recording from patches excised from the outer nuclear envelope of DT40-IP_3_R1 cells, with K^+^ as charge-carrier and using QuB (Qin, 2004) for analysis of currents, were as described previously (Taufiq-Ur-Rahman et al., 2009; Rossi et al., 2009). It typically took 30-45 s to establish a recording, and *P*_o_ was then stable throughout recordings that typically lasted up to 8-10 mins.

#### Western blotting

Cells were harvested in radio-immunoprecipitation assay (RIPA) buffer (150 mM NaCl, 1.0% NP-40 or Triton X-100, 0.5% sodium deoxycholate, 0.1% SDS, 50 mM Tris, pH 8.0) with protease inhibitor cocktail. The cell suspension was passed through a 25-gauge needle, incubated for 30 min at 4°C with rotation, sonicated (3 × 10 s pulses on ice), and then cleared by centrifugation (14,000 *× g*, 60 min, 4°C). Proteins were separated using 3%–8% or 4%–12% SDS-PAGE gels (NuPAGE), and transferred to PVDF membranes using an iBlot system. Antibodies are listed in the Key Resources table. Proteins were visualized using ECL Prime/Select western blotting detection reagents and the GeneGnome imaging system (SynGene, Cambridge, UK) and chemiluminescence was quantified using Fiji (Schindelin et al., 2012). For all quantitative analyses, we used bands for which we confirmed, by serial dilution of samples, a linear relationship between protein loading and band intensity.

### Quantification and statistical analyses

#### Simulations

Simulations first consider the number of open IP_3_Rs after sequential additions of IP_3_. The scheme assumes that IP_3_Rs to which four IP_3_ molecules have bound move between three stable states, closed (C_4_), open (O_4_) and inactivated (I_4_), each with similar affinity for IP_3_ (Figure 7E). Transitions between receptors states are described by a triangular kinetic scheme (Tveito and Lines, 2010), with *k*_1_ = 0.4 s^-1^, *k*_2_ = 0.2 s^-1^ and *k*_3_ = 0.004 s^-1^, but the scheme includes an irreversible step in a way that is reminiscent of a published model for adaptation in cell signaling (Ferrell, 2016). The transitions between these states (rate constants, *k*_*i*_, 0.004 - 0.4 s^-1^, and 100-times slower for *k*_*-i*_) are much slower than the rates of IP_3_ association (∼3.5 × 10^7^ M^-1^.s^-1^; k_on_ ∼3.5 s^-1^ with 100 nM IP_3_) (Hannaert-Merah et al., 1994) or dissociation (∼28 s^-1^, estimated from the K_D_ in Figure 2G). Our scheme therefore assumes that for each IP_3_ concentration, there is a fixed number of IP_3_Rs with all four IP_3_-binding sites occupied, determined by the ${\text{K}}_{\text{D }}^{{\text{IP}}_{3}}$ (794 nM) and the number of IP_3_Rs/cell (49,146 in HEK-IP_3_R1 cells) (Figure 7B). IP_3_ addition is simulated by an immediate increase in C_4._ Simulations are performed stochastically, with each IP_3_R simulated independently. The probability of transition i is equal to *k*_i_. Δt. At each time step (12.5 ms), the algorithm generates a number X drawn uniformly at random on the [0,1] interval. If X < *k*_i_. Δt, the IP_3_R changes state; otherwise it remains in the same state. The behavior of the IP_3_R population is obtained by summing the number of IP_3_Rs in each state at every time step. The output provides the temporal evolution of the number of open IP_3_Rs. The same scheme and parameters were used to simulate the behavior of IP_3_Rs within a single wild-type HEK cell, for which we determined the expression level to be 9101 ± 934 IP_3_Rs/cell.

To simulate the change in ER free [Ca^2+^] (*C*_ER_) over time, we consider the following equation:

(1) $$\frac{dC_{ER}}{dt}=-\left(k_{leak}+k_{rel}.O_{4}\right).\left(C_{ER}-C\right)$$

where *O*_*4*_ is the number of open IP_3_Rs determined from the stochastic simulations, *k*_*leak*_ is the rate constant for the IP_3_-independent Ca^2+^ leak from the ER (1.8 × 10^−4^ s^-1^, see *Analysis* section), *k*_rel_ is the rate constant for Ca^2+^ flux through an open IP_3_R (0.0008 s^-1^). We estimated *k*_*rel*_ by assuming that the free [Ca^2+^] within the ER was 500 μM, the buffer capacity was 5 (hence, total ER [Ca^2+^] = 2.5 mM; see *Analysis* section), and an open IP_3_R conducts 5 × 10^5^ Ca^2+^ s^-1^ (Vais et al., 2010). *C*, the cytosolic free [Ca^2+^], is fixed at 220 nM by the EGTA included in CLM. At the beginning of each simulation, *C*_*ER*_ is set to 500 μM (see Rossi and Taylor, 2020) and then computed at each time step for the corresponding *O*_*4*_. The fraction of releasable ER Ca^2+^ was set to 80%.

To simulate experiments where the ER can refill through SERCA, we used:

(2) $$\;\frac{dC_{ER}}{dt}=-\left(k_{leak}+k_{rel}.O_{4}\right).\left(C_{ER}-C\right)+\left(500-C_{ER}\right)/\tau_{SERCA}$$

with $\tau_{SERCA}$ = 100 s.

Codes for the simulations are available from https://github.com/genedupont/Quantal.

#### Analysis

Rates of Ca^2+^ release evoked by IP_3_, ionomycin, carbachol or **2**, corrected for the basal Ca^2+^ leak (*J*_leak_), were calculated by fitting a mono-exponential function to the ER Ca^2+^ content determined from the time of adding CPA alone. The derivative of this relationship at each time provides *J*_leak_. From the relationship between *J*_leak_ and ER Ca^2+^ content (fitted to a second-degree polynomial or exponential function), *J*_leak_ was estimated at each time and subtracted from the observed Ca^2+^ release to identify the Ca^2+^ release evoked by IP_3_, ionomycin, carbachol or **2** (Figures 1F, 1L, 3B, S3D, and S4B).

For each IP_3_ or ionomycin concentration, the time evolution of ER free [Ca^2+^] ([Ca^2+^]_ER_) was then numerically reconstructed by integration over time of the IP_3_- or ionomycin-evoked Ca^2+^ flux. *J*_*leak*_ values from [Ca^2+^]_ER_ of 500 μM were also used to estimate the rate constant of Ca^2+^ leak from the ER (*k*_*leak*_ = 1.8x10^−4^ s^-1^) used in the simulations. To obtain the evolution of open receptors (Figures 7R–7T), the leak-independent Ca^2+^ flux was subtracted from the rate of Ca^2+^ release before division of each instantaneous rate by the corresponding Ca^2+^ gradient. Following Equation (1), this gives the time-evolution of the number of open receptors in arbitrary units.

To estimate the total ER Ca^2+^ content of a single HEK cell, we assume a luminal free [Ca^2+^] of ∼500 μM (Rossi and Taylor, 2020), a buffer ratio of ∼5 (Prins and Michalak, 2011) (suggesting a total luminal [Ca^2+^] of ∼2.5 mM); a cell volume of 1.1 × 10^12^ L (http://bionumbers.hms.harvard.edu/search.aspx) of which ∼10% is occupied by the nucleus and ∼35% of the remaining cytoplasm is occupied by ER (∼3.64 × 10^−13^ L) (Valm et al., 2017). Hence, we estimate the total ER Ca^2+^ content within a single HEK cell to be ∼9 × 10^−16^ mol.

For equilibrium binding and concentration-effect relationships, results from each experiment were fitted to Hill equations (PRISM) from which pIC_50_ (-log of half-maximal inhibitory concentration) or pEC_50_ (-log of half-maximally effective concentration) values were determined. ${\text{K}}_{\text{D}}^{\text{ligand}}$ was calculated from: ${\text{K}}_{\text{D}}^{\text{ligand}}$ = ${\text{IC}}_{50}^{\text{ligand}}$ - [^3^H-IP_3_] $/{\text{K}}_{\text{D}}^{\text{IP}3}$. In functional assays, dose ratios (DR) were used to calculate antagonist affinities (Arunlakshana and Schild, 1959): ${\text{K}}_{\text{D}}^{\text{antagonist}}$ = [antagonist]/(DR-1). Statistical analyses of IP_3_ sensitivity used pEC_50_ and pK_D_ values.

##### Statistical analyses

Experiments were performed without prior power calculations, blinding or systematic randomization. The distribution of treatments across wells in multi-well plates was varied between experiments to avoid any systematic bias arising from place-related effects. Statistical significance was assessed using Student’s t test or one-way repeated ANOVA followed by Bonferroni’s multiple comparisons test, with *^∗^p <* 0.05 considered significant (PRISM). The numbers of independent experiments and replicates for each biological experiment are indicated in figure legends. Details of all statistical tests and exact *p*values are provided in Supplemental materials and methods.

## Data Availability

No large datasets were generated by this study. The computer code used for the simulations is available at: https://github.com/genedupont/Quantal. Any additional information required to reanalyze the data reported in this paper is available from the lead contact upon request.

## Supplemental information

Document S1. Tables S1–S4, Figures S1–S7, and Methods S1

Document S2. Article plus supplemental information
