# Supplementary material for: Quantal Ca2+ release mediated by very few IP3 receptors that rapidly inactivate allows graded responses to IP3
Source: Cell Rep. 2021 Nov 2;37(5):109932. doi: 10.1016/j.celrep.2021.109932 (PMC8578705; doi:10.1016/j.celrep.2021.109932)
Supplement: Document S1. Tables S1–S4, Figures S1–S7, and Methods S1 [file mmc1.pdf]

Cell Reports, Volume *37*

Supplemental information

Quantal Ca^{2+} release mediated by very few IP_3 receptors that rapidly inactivate allows graded responses to IP_3

Ana M. Rossi, Andrew M. Riley, Geneviève Dupont, Taufiq Rahman, Barry V.L. Potter, and Colin W. Taylor

SUPPLEMENTAL MATERIALS

Table S1. Properties of IP_3 and 2
Related to Figure 2.

	IP_3	**2**
**Binding**		
IP_3R1, pK_D (K_D)	7.90 ± 0.06 (12.4 nM), n = 7	8.28 ± 0.05*** (5.2 nM), n = 8
NT, pK_D (K_D)	8.60 ± 0.05 (2.48 nM), n = 4	8.63 ± 0.03 (2.32 nM), n = 4
IBC, pK_D (K_D)	9.62 ± 0.09 (0.24 nM), n = 4	9.36 ± 0.06** (0.44 nM), n = 4
$^{a}\Delta\Delta G$	-5.2 kJ/mol	-3.8 kJ/mol
**Ca^{2+} release**		
Release	71.3 ± 0.9%, n = 3	51.9 ± 0.7%***, n = 3
pEC_{50} (EC_{50})	7.38 ± 0.12 (41.6 nM), n = 3	6.32 ± 0.22* (479 nM), n = 3
$^{b}EC^{I}_{50}$	41.6 nM	832 nM
pK_D-pEC^{I}_{50} (EC^{I}_{50}/K_D)	0.53 ± 0.11 (3.4), n = 10	2.15 ± 0.15* (141), n = 10
**Electrophysiology**		
NP_o	0.48 ± 0.02, n = 6	0.012 ± 0.003****, n = 6
γ_K (pS)	219 ± 3, n = 6	217 ± 4, n = 6
τ_o (ms)	9.4 ± 0.17, n = 6	4.4 ± 0.20****, n = 6
τ_c (ms)	10.6 ± 1.04, n = 6	^{c}ND

Functional responses were recorded from DT40-IP_3R1 cells. IP_3 binding assays were performed in TEM using purified cerebellar IP_3R (IP_3R1) or bacterially-expressed fragments of IP_3R1 (NT and IBC). Electrophysiology results compare the effects of 10 µM IP_3 and **2**. Results show mean ± SEM from n independent experiments. $^{*}P < 0.05$, $^{**}P < 0.01$, $^{***}P < 0.001$, $^{****}P < 0.0001$, Student's t-test, relative to IP_3. $^{a}\Delta\Delta G$ (ΔG^{IBC} - ΔG^{NT}) reports the difference in ΔG for IP_3 binding to the IBC and NT. $^{b}EC^{I}_{50}$ denotes the concentration of each ligand required to cause Ca^{2+} release equivalent to that evoked by a half-maximally effective concentration of IP_3 (the EC_{50} for IP_3). ^{c}ND, not determined because the very low NP_o with **2** does not allow single- and multi-channel patches to be unambiguously distinguished. It is, therefore, impossible to determine whether intervals between openings are gaps between openings of the same channel (τ_c) or between openings of different channels. τ_o, τ_c mean channel open and closed times. γ_K, single-channel K^+ conductance derived from current-voltage relationships.

Table S2. Properties of IP_3 and 2 in wild-type HEK cells
Related to Figure 2.

	IP_3	**2**
pEC_{50}	6.71 ± 0.10	6.09 ± 0.22*
Ca^{2+} release (%)	69 ± 1	51 ± 7*
h	1.0 ± 0.1	1.1 ± 0.3

Effects of IP_3 or **2** on Ca^{2+} release from permeabilized wild-type HEK cells (mean ± SEM, *n* = 3-4, each with duplicate determinations). ER Ca^{2+} content was measured 20 s after addition of IP_3 or **2**. $^{*}P < 0.05$, Student's *t*-test, relative to IP_3.

Table S3. Affinity of 2 for IP_3R determined from functional analyses
Related to Figure 2.

		IP_3-induced Ca^{2+} release		
**2** (μM)	Ca^{2+} release evoked by **2** (%)	pEC_{50} EC_{50} (nM)	Ca^{2+} release (%)	*n*
0	0	7.40 ± 0.03 39.4	68.0 ± 3.5	8
1	31 ± 7	6.88 ± 0.09*** 131.8	74.3 ± 4.6	5

From the experiments shown in **Figure 2F**, we determined the Ca^{2+} release evoked by **2 (**1 μM), and then the effects of IP_3, added 30 s after **2**, on the residual Ca^{2+} stores. Results show mean ± SEM from *n* independent experiments, each with 3 replicates (mean only for EC_{50}). $^{***}P < 0.001$, Student's *t*-test. From the ratio of the two EC_{50} values for IP_3-evoked Ca^{2+} release (dose ratio, DR), the K_D for **2** was calculated: $K_D = [\mathbf{2}]/(DR-1) = 426$ nM.

Table S4. Bioassays of IP_3 after prolonged incubation with permeabilized cells Related to Figure 5L.

	Pre-incubated	Control
pEC_{50}	6.76 ± 0.09	6.76 ± 0.09
Ca^{2+} release (%)	85 ± 8	86 ± 5
h	0.95 ± 0.1	1.6 ± 0.5

IP_3 is not metabolized after prolonged incubation with permeabilized cells. IP_3 was incubated in 96-well plates with permeabilized HEK-IP_3R1 cells (pre-incubated) or without cells (control) for 260 s under conditions identical to those used in **Figure 5G**. The supernatants from these plates (650 ×*g*, 1 min) was then added to fresh permeabilized cells loaded to steady state with Ca^{2+} to determine the Ca^{2+}-releasing activity of the extracts (**Figure 5L**). The table (mean ± SEM, $n = 4$) shows no significant differences between the pEC_{50}, maximal release (%) or Hill coefficient (*h*) values between cells stimulated with IP_3 that had been pre-incubated with cells or under control conditions

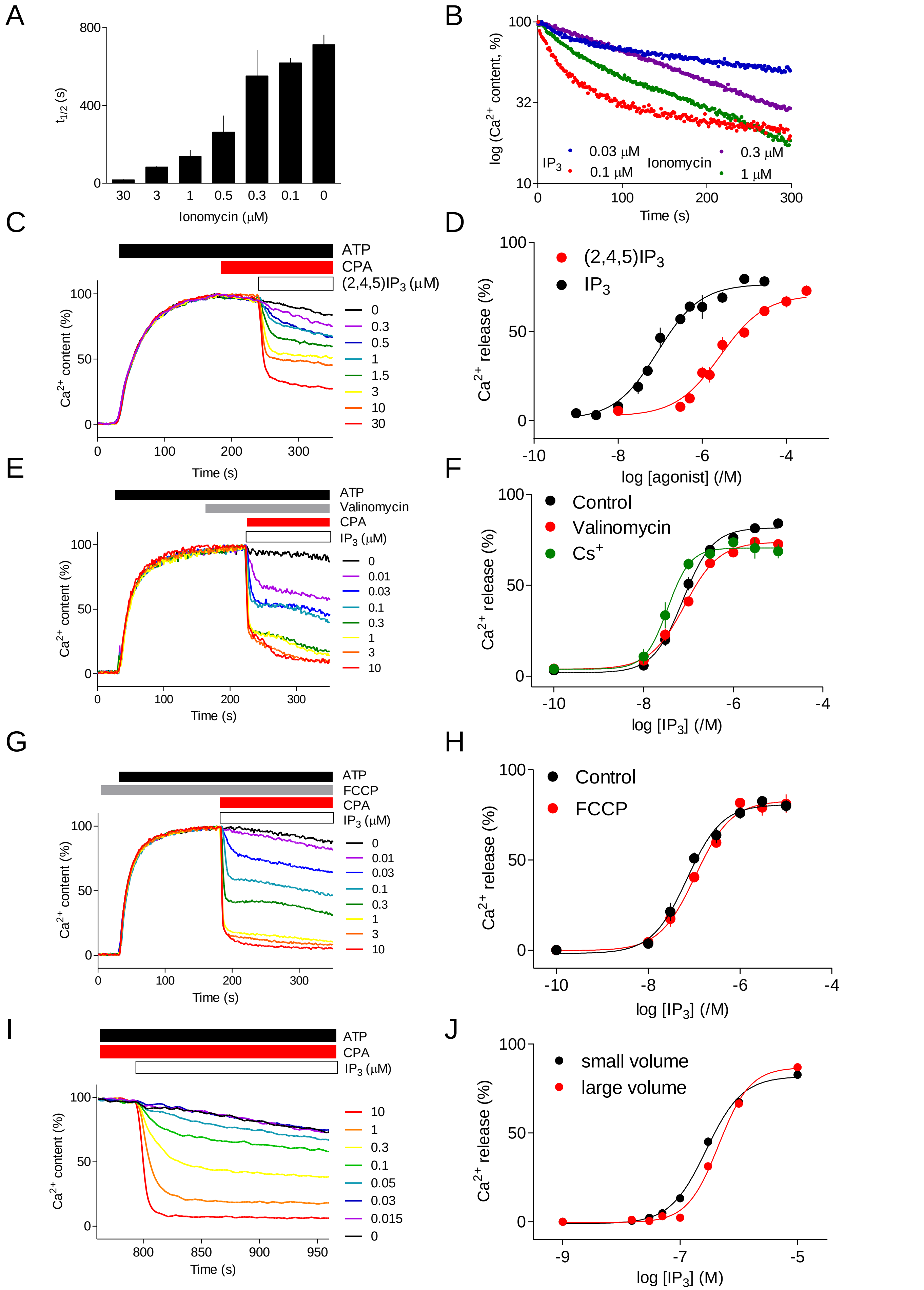

Figure S1. Quantal responses in permeabilized cells are not due to IP_3 metabolism, ineffective movement of counter-ions, contributions from mitochondria or bolus dddition of IP_3

Legend on next page

Figure S1. Quantal responses in permeabilized cells are not due to IP_3 metabolism, ineffective movement of counter-ions, contributions from mitochondria or bolus dddition of IP_3
Related to Figure 1.

Figure on preceding page

(**A**) Half-times ($t_{1/2}$) for Ca^{2+} release evoked by the indicated concentrations of ionomycin (mean ± SEM, *n* = 4) from permeabilized DT40-IP_3R1 cells (from experiments similar to those shown in **Figure 1E**).
(**B**) Semi-logarithmic plots show mono-exponential loss of ER Ca^{2+} from permeabilized DT40-IP_3R1 cells after treatment with ionomycin, and quantal release evoked by IP_3 (from data shown in **Figures 1D** and **1E**).
(**C**) Quantal Ca^{2+} release from permeabilized HEK-IP_3R1 cells evoked by (2,4,5)$IP_{3,}$ a non-metabolized analogue of IP_3 (Hill et al., 1988). Results, typical of 4 experiments, show means for 4 replicates.
(**D**) Summary results show concentration-dependent effects of IP_3 and (2,4,5)IP_3 (mean ± SEM, *n* = 4, each with 3 replicates).
(**E**) Quantal responses to IP_3 in permeabilized DT40-IP_3R1 cells in the presence of valinomycin (10 µM, 1 min) to allow exchange of K^+ across ER membranes. Mean of duplicate determinations.
(**F**) Summary results (mean ± SEM, *n* = 3-4, each with duplicate determinations) show concentration-dependent quantal Ca^{2+} release by IP_3 in normal CLM, CLM with Cs^+ replacing all K^+ to inhibit K^+ channels, or CLM with valinomycin (10 µM).
(**G**) Quantal responses of permeabilized DT40-IP_3R1 cells to IP_3 in the presence of FCCP (10 µM, 10 min) to inhibit mitochondria. Results, typical of 3 or 4 experiments, show means for 2 replicates.
(**H**) Summary results show concentration-dependent effects of IP_3 alone or with FCCP (mean ± SEM, *n* = 3-4, each with duplicate determinations).
(**I**) Permeabilized HEK-G-CEPIA1*er* cells in 50 µL of CLM were loaded to steady state with Ca^{2+}; CPA (10 µM) and IP_3 (at final concentrations shown) were then added. IP_3 was added in 50 µL of CLM (at 2-times its final concentration; rather than the usual 5 µL at 10-times the final concentration). The aim was to avoid artefacts that might arise from a bolus addition of IP_3 at ten-times its final concentration exposing cells near the injection site to transiently high IP_3 concentrations. Traces from one experiment with 2 replicates are typical of 5 experiments.
(**J**) Summary results (mean ± SEM, *n* = 5, each with 2 replicates; most error bars are smaller than the symbols) show Ca^{2+} release evoked by IP_3 for assays in which the stimulus was added as a 50-µL (large volume) or 5-µL addition (small volume). ER Ca^{2+} content was measured 20 s after addition of (2,4,5)IP_3 (**D**) or IP_3 (**G, H, J**).

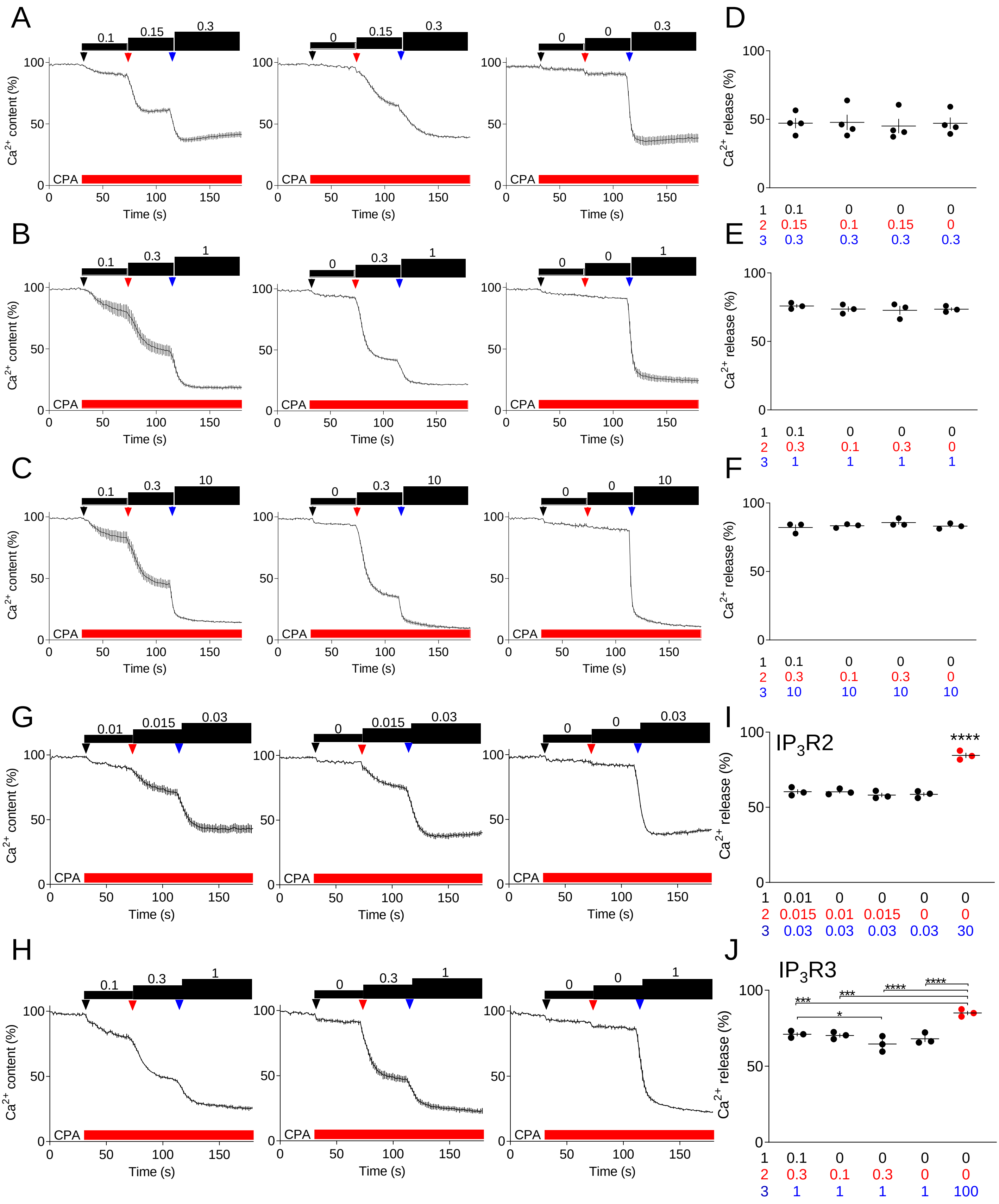

Figure S2. Incremental responses do not require an increase in $[Ca^{2+}]_c$ and occur with all IP_3R subtypes

Legend on next page

Figure S2. Incremental responses do not require an increase in $[Ca^{2+}]_c$ and occur with all IP_3R subtypes
Related to Figure 1.

Figure on preceding page

(**A-C**) In experiments similar to those shown in **Figure 1D**, permeabilized DT40-IP_3R1 cells were used to examine the effects of incremental additions of IP_3 in CLM containing 10 mM BAPTA (cumulative IP_3 concentrations shown in µM). Mean ± SD of 3 replicates.
(**D-F**) Summary results show Ca^{2+} release recorded 20 s after the final IP_3 addition for each sequence of incremental additions (individual values, mean ± SEM, n = 3-4, each with 3 replicates). Since BAPTA is a low-affinity competitive IP_3R antagonist (Richardson and Taylor, 1993), the sensitivity to IP_3 is reduced in the presence of 10 mM BAPTA. There were no significant differences between any of the incremental additions (**D-F**), one-way repeated ANOVA with Bonferroni's multiple comparisons test.
(**G, H**) Experiments similar to those shown in **Figures 1G-1J** show incremental responses to additions of IP_3 (cumulative IP_3 concentrations shown in µM) for permeabilized DT40 cells expressing IP_3R2 (G) or IP_3R3 (H) (mean ± SD, 3-6 replicates).
(**I, J**) Summary results (individual values, mean ± SEM, n = 3, each with 3-6 replicates). The responses to maximal concentrations of IP_3 (red symbols) confirm that all incremental additions evoked submaximal responses. $^{***}P < 0.001$, $^{****}P < 0.0001$, one-way repeated ANOVA with Bonferroni's test; there were no significant differences between any of the incremental additions, except where shown ($^{*}P < 0.05$). Ca^{2+} release was recorded 20 s after the final IP_3 addition for each sequence of incremental additions.

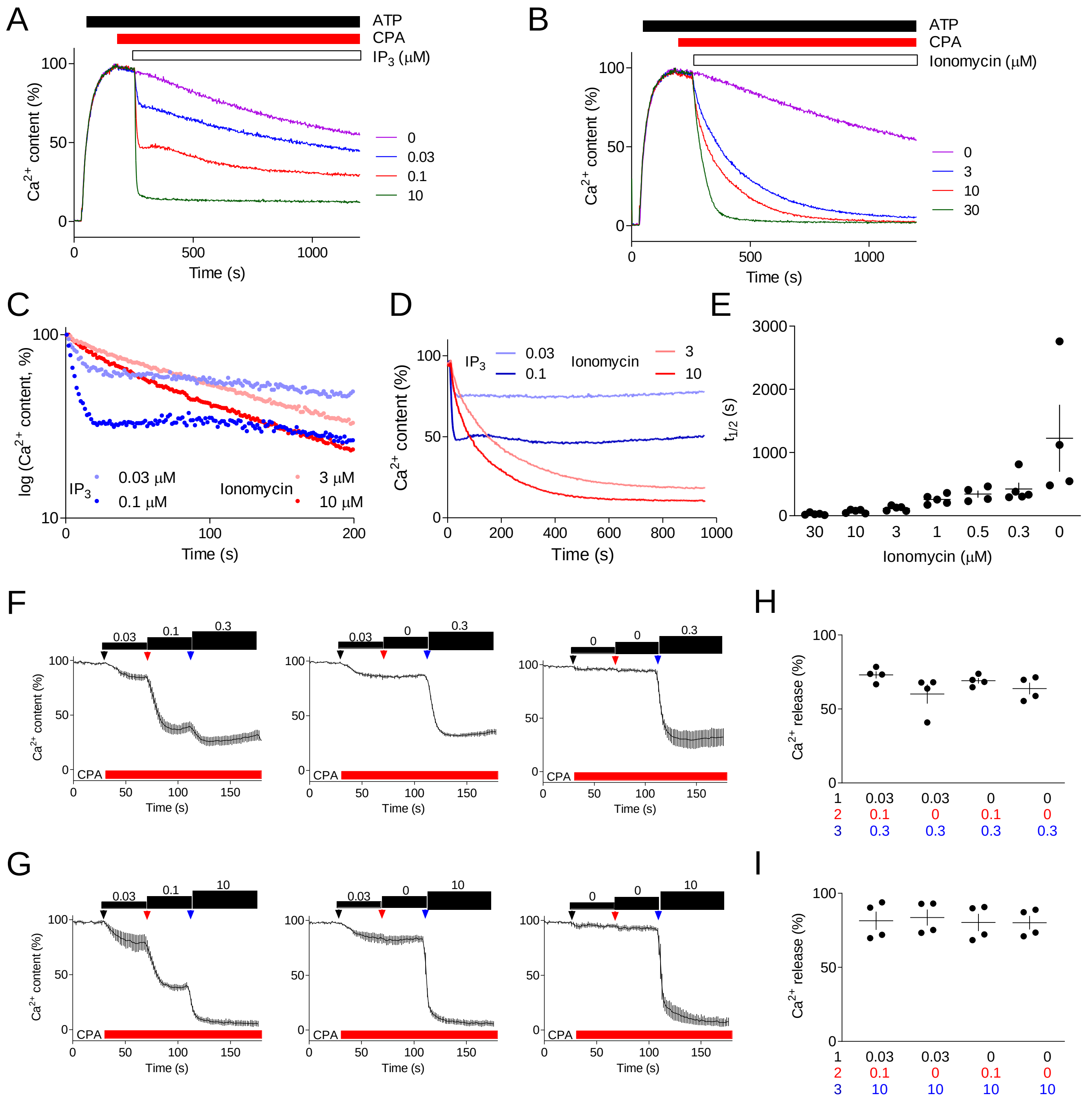

Figure S3. Incremental responses to IP_3 in permeabilized wild-type HEK cells Related to Figure 1.

(A, B) Effects of the indicated concentrations of IP_3 (**A**) or ionomycin (**B**) added to permeabilized wild-type HEK cells in which the intracellular stores had been loaded to steady state with Ca^{2+}, and with Mag-fluo 4 used to record ER luminal [Ca^{2+}]. CPA (10 µM) was added to inhibit SERCA. Results, typical of 5 independent experiments, show the mean for 2 replicates.
(**C**) Semi-logarithmic plots show the monophasic Ca^{2+} release evoked by ionomycin, and the quantal response to IP_3.
(**D**) Responses to IP_3 or ionomycin after subtraction of the basal Ca^{2+} leak.
(**E**) Summary results show half-times ($t_{1/2}$) for the loss of ER Ca^{2+} evoked by the indicated concentrations of ionomycin (individual values, mean ± SEM, n = 4-5, each with 2 replicates).
(**F, G**) Effects of submaximal (0.3 µM, **F**) or maximal (10 µM, **G**) concentrations of IP_3 added directly or by incremental additions (cumulative IP_3 concentrations shown in µM). Mean ± SD of 3 replicates.
(**H, I**) Summary results (individual values, mean ± SEM, n = 4, each with 3 replicates) show the final Ca^{2+} content of the stores (determined at 150 s) for the incremental additions (1-3, cumulative concentrations, µM). No significant differences between the incremental additions (one-way repeated ANOVA).

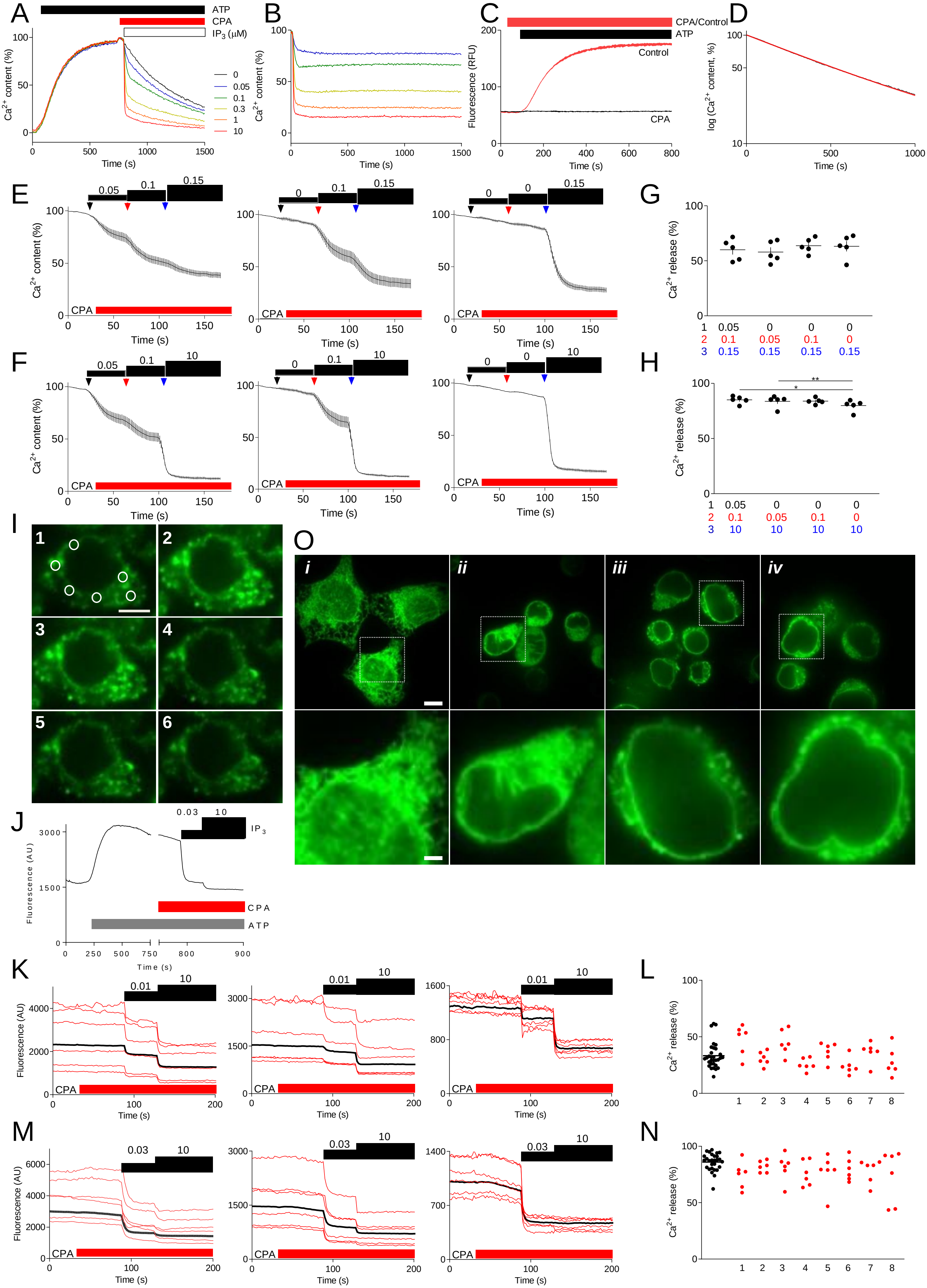

Figure S4. Quantal responses to IP_3 recorded using G-CEPIA1*er*
Legend on next page

Figure S4. Quantal responses to IP_3 recorded using G-CEPIA1*er*
Related to Figure 1.

Figure on preceding page

(**A**) Populations of HEK cells stably expressing the genetically encoded Ca^{2+} indicator, G-CEPIA1*er* (Suzuki et al., 2014) (K_D = 1.55 mM determined *in situ*) (Rossi and Taylor, 2020), were permeabilized in CLM before addition of ATP (1.5 mM), CPA (10 µM) and then IP_3 (concentrations in µM). Results show means of 3 replicates, typical of 5 experiments.
(**B**) Responses to IP_3 (from panel **A**) shown after subtraction of the basal Ca^{2+} leak.
(**C**) Addition of ATP (1.5 mM) to a population of permeabilized cells stimulates Ca^{2+} uptake into the ER, reported by the increase in G-CEPIA1*er* fluorescence. The response is abolished by CPA (10 µM) added 60 s before ATP. Results show traces (mean ± SD, each with 4 replicates) typical of 4 independent experiments. RFU, relative fluorescence units.
(**D**) Stores were loaded with Ca^{2+} before addition of CPA. The subsequent fluorescence changes recorded from the cell population are plotted semi-logarithmically. The half-time ($t_{1/2}$) is 445 ± 41 s and the intercept at time 0 (CPA addition) is 100.9 ± 0.4 % (mean ± SEM, n = 7, each with 12-16 replicates). The latter confirms the linear relationship between ER free [Ca^{2+}] and fluorescence across the entire range of ER Ca^{2+} contents examined, suggesting that in replete stores the free [Ca^{2+}] is less than the K_D of the indicator (1.55 mM). Similar estimates for the rate of Ca^{2+} leak determined using Mag-fluo 4 ($t_{1/2}$ = 356 ± 52 s) (Rossi and Taylor, 2020) and G-CEPIA1*er* ($t_{1/2}$ = 445 ± 41 s) confirm the reliability of measurements with Mag-fluo 4 and G-CEPIA1*er*.
(**E, F**) Incremental responses from populations of permeabilized cells to the indicated concentrations of IP_3 added after CPA (10 µM), recorded using G-CEPIA1*er* (cumulative IP_3 concentrations shown in µM).
(**G, H**) Summary results (individual values, mean ± SEM, n = 5, each with 3 replicates) show final Ca^{2+} content of the stores (determined at 150 s) for the incremental additions (cumulative IP_3 concentrations shown in µM). No significant differences, except where indicated ($^{*}P < 0.05$, $^{**}P < 0.01$, one-way repeated ANOVA with Bonferroni's test).
The results (**E-H**) demonstrate that incremental responses to IP_3 are not an artefact arising from use of luminal Ca^{2+} indicators failing to reliably report luminal [Ca^{2+}] (see also Rossi and Taylor, 2020).
(**I**) HEK-G-CEPIA1*er* cells were permeabilized before addition of ATP, then CPA (10 µM) to inhibit SERCA, and IP_3. Confocal sections show a single permeabilized cell before (1) and 700 s after addition of ATP (2), immediately before (3) and 20 s after adding 30 nM IP_3 (4), and before (5) and 20 s after (6) addition of 10 µM IP_3. Scale bar = 5 µm. Circles show typical subcellular regions of interest (ROI, 1.5-µm diameter) selected for the analyses shown in panels **K-N**.
(**J**) Time-course of the fluorescence changes recorded from an entire cell.
(**K**) Three examples of whole-cell responses (black lines) and ROI within each cell (red lines) to addition of CPA and then sequential stimulation with 10 nM and then 10 µM IP_3. AU, arbitrary units.
(**L**) Summary results (individual values, mean ± SD, n = 31 cells) show responses to 10 nM IP_3 measured 20 s after its addition (% of IP_3-sensitive Ca^{2+} stores) in whole cells (black) and 6 randomly selected ROIs (red) from within 8 cells (1-8).
(**M**) Examples of responses from entire cells (black lines) and ROI within each cell (red lines) to addition of CPA and then sequential stimulation with 30 nM and then 10 µM IP_3. In most cells (28/33), 30 nM IP_3 released only a fraction of the IP_3-sensitive stores (first two examples), but in a few cells (5/33) it evoked a maximal response (last example).
(**N**) Summary results (individual values, mean ± SD, n = 28 cells) show responses to 30 nM IP_3 measured 20 s after its addition in whole cells (black) and 6 randomly selected ROIs (red) within 8 cells (1-8) in which 30 nM IP_3 evoked a submaximal response (% of IP_3-sensitive Ca^{2+} stores).
These results (**K-N**) establish that quantal responses to IP_3 occur within single cells and they show that there is no evident heterogeneity in IP_3 sensitivity between ROIs within single cells.
(**O**) Confocal images of intact HEK-G-CEPIA*er* cells grown on poly-L-lysine-coated dishes ***(i)***, and HEK-G-CEPIA*er* cells after trypsinization and attachment to poly-L-lysine-coated dishes ***(ii);*** HEK-G-CEPIA*er* cells ***(iii)*** and HEK cells loaded with Mag-fluo 4 (***iv***) after permeabilization and attachment to poly-L-lysine-coated dishes. Scale bar = 10 µm (2.5 µm in enlargements). Results are typical of 3 independent experiments (~150 cells in each). The results show that there is partial fragmentation of the ER after permeabilization, which is similar for both Ca^{2+} indicators.

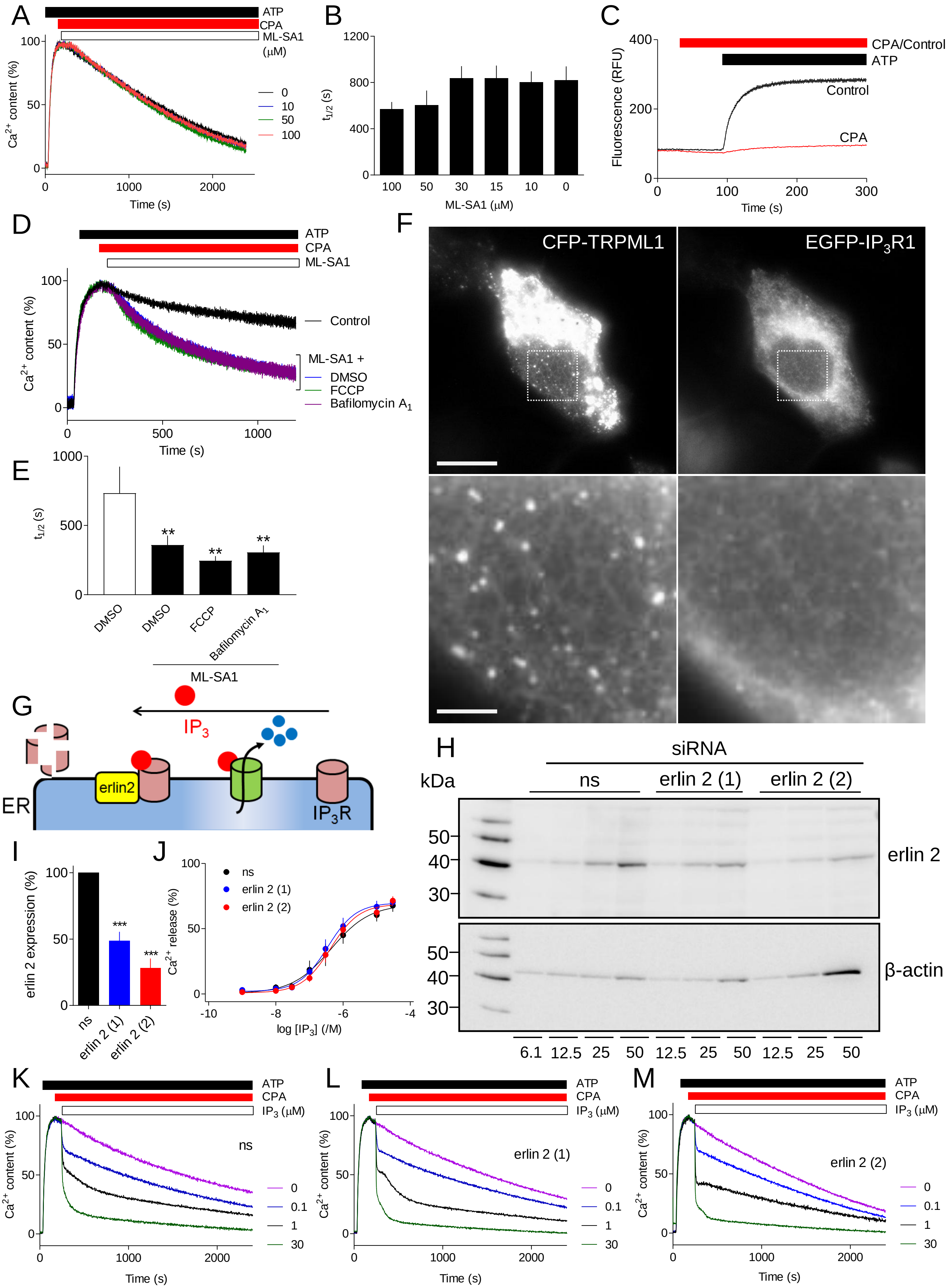

Figure S5. Evidence that over-expressed TRPML1 releases Ca^{2+} from ER, and that erlin 2 does not mediate quantal Ca^{2+} release

Legend on next page

Figure S5. Evidence that over-expressed TRPML1 releases Ca^{2+} from ER, and that erlin 2 does not mediate quantal Ca^{2+} release
Related to Figure 4.

Figure on preceding page

(**A**) Experiments conducted in parallel with those shown in **Figure 4A** demonstrate that mock-transfected HEK cells do not respond to ML-SA1. Ca^{2+} uptake into the ER of permeabilized HEK cells was initiated by addition of ATP (1.5 mM), before adding CPA (10 µM) and then the indicated concentration of ML-SA1 (0-100 µM). Results show mean ± SD of 3 replicates, typical of 4 independent experiments.
(**B**) Summary results from experiments similar to those shown in **Figure 4A** show half-times ($t_{1/2}$) for loss of ER Ca^{2+} evoked by the indicated concentrations of ML-SA1 (mean ± SEM, n = 4, each with 2 replicates).
(**C**) Addition of ATP (1.5 mM) to permeabilized HEK cells stimulates Ca^{2+} uptake into the ER, reported by the increase in Mag-fluo 4 fluorescence. The response is abolished by CPA (10 µM) added 60 s before ATP. Mean ± SEM, n = 4, each with 2 replicates. RFU, relative fluorescence units. These results demonstrate that Mag-fluo 4 selectively reports ER luminal [Ca^{2+}].
(**D**) Permeabilized HEK cells expressing CFP-TRPML1 were treated (60 min) with vehicle (DMSO), bafilomycin A_1 (1 µM to inhibit the lysosomal H^+ pump) or FCCP (10 µM to dissipate H^+ gradients) before addition of ATP (1.5 mM) to initiate Ca^{2+} uptake. After addition of CPA (10 µM), ML-SA1 (30 µM) was added where indicated. Mean ± SD of 3 replicates.
(**E**) Summary results (mean ± SEM, n = 4-5, each with 3-6 replicates) show half-times ($t_{1/2}$) for the mono-exponential rates of Ca^{2+} release after each treatment. $^{**}P < 0.01$, one-way ANOVA with Bonferroni's test, relative to DMSO with no ML-SA1. These results demonstrate that TRPML1, which is natively expressed in lysosomes, releases Ca^{2+} from the ER when it is over-expressed. We note that our assay further ensures that we measure only ER Ca^{2+} release because Mag-fluo 4 selectively reports Ca^{2+} uptake into the ER (Rossi and Taylor, 2020).
(**F**) TIRFM images show expression of CFP-TRPML1 and EGFP-IP_3R1 in HEK cells expressing both proteins, typical of 3 independent experiments (~10 cells in each). Scale bar = 20 µm (4 µm for enlargement). As expected, CFP-TRPML1 is expressed most in lysosomes, but there is an underlying reticular distribution that is similar to that of EGFP-IP_3R1. It was impracticable, when most CFP-TRPML1 is lysosomal, to quantify any colocalization with IP_3Rs. We note that our use of Ca^{2+} indicators that exclusively report ER luminal [Ca^{2+}] ensure that in functional assays, we detect only the behaviour of TRPML1 expressed in the ER.
(**G**) IP_3 binding promotes both opening of the IP_3R channel and association of IP_3R with erlin 2, which targets the IP_3R for ubiquitination and degradation (Wojcikiewicz, 2018). We considered whether rapid association of active IP_3Rs with erlin 2 might inactivate them before their degradation.
(**H**) Western blot using an antibody against erlin 2 or β-actin (loading control) of lysates prepared from HEK cells treated with a non-silencing siRNA (ns) or either of two different siRNAs against erlin 2. Protein loadings (µg) are shown beneath each lane, and molecular weight markers are shown on the left. Since the molecular weight of erlin 2 is similar to that of β-actin (and most other loading controls), two identical gels were run, one blotted with erlin 2 and the other with β-actin.
(**I**) Summary results (mean ± SEM, n = 4) show erlin 2 expression relative to cells treated with ns siRNA. Knock-down efficiency of siRNA erlin 2 (1) and (2) was 51 ± 7% and 72 ± 7%, respectively. $^{***}P < 0.001$, one-way ANOVA with Bonferroni's test.
(**J**) Methods similar to those in **Figure 1D** were used to determine the effects of IP_3 on Ca^{2+} release from the intracellular stores of permeabilized HEK cells after treatment with the indicated siRNAs. ER Ca^{2+} content was measured 20 s after addition of IP_3. Mean ± SEM, n = 4, each with 3 replicates.
(**K-M**) Ca^{2+} content of the ER of permeabilized HEK cells is shown after addition of ATP (1.5 mM), followed by CPA (10 µM) and then the indicated concentrations of IP_3 (µM) in cells treated with ns siRNA (**K**) or siRNAs directed to erlin 2 (**L, M**). Results (mean of 3 replicates) show Ca^{2+} contents relative to steady-state Ca^{2+} content (%). Similar results were obtained in 4 independent experiments.

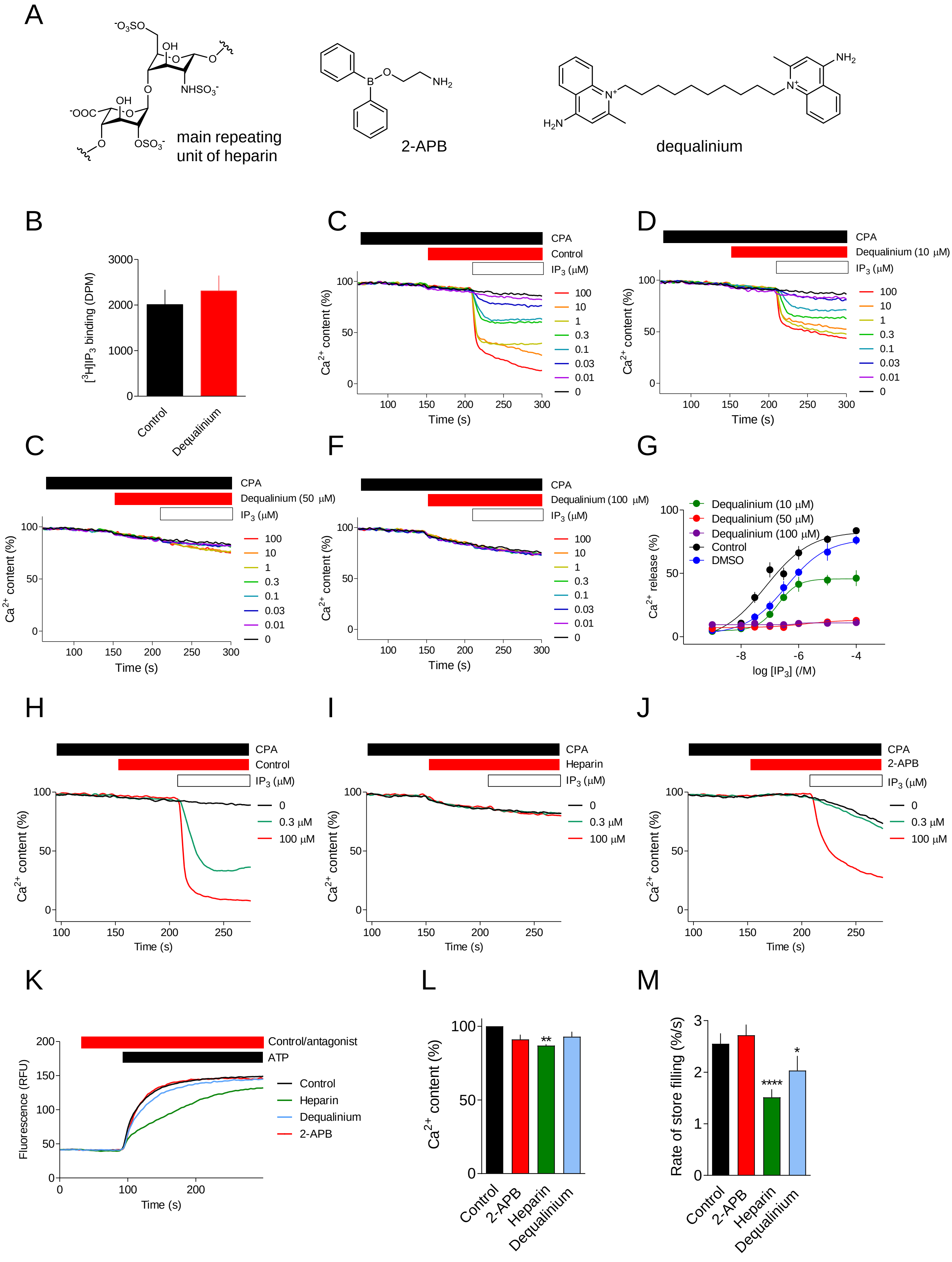

Figure S6. Effects of heparin, 2-APB and dequalinium on IP_3Rs and Ca^{2+} uptake by intracellular stores

Legend on next page

Figure S6. Effects of heparin, 2-APB and dequalinium on IP_3Rs and Ca^{2+} uptake by intracellular stores
Related to Figures 5 and 6.

Figure on preceding page

(**A**) Structures of the antagonists used.
(**B**) Effects of dequalinium (50 μM) on specific $[^3H]IP_3$ (7.5 nM) binding to cerebellar membranes in CLM at 20°C. Mean ± SEM, *n* = 3. Heparin is a competitive antagonist of IP_3, while 2-APB (like dequalinium) does not affect IP_3 binding (Saleem et al., 2014). No significant difference, Student's *t*-test.
(**C-F**) Permeabilized HEK-IP_3R1 cells were loaded with Ca^{2+}, and SERCA was then inhibited with CPA (10 μM) before addition of DMSO (1% v/v; **C**) or dequalinium (10-100 μM, **D-F**), and then the indicated concentrations of IP_3. Mean of 2 replicates, typical of 5 experiments.
(**G**) Summary results (mean ± SEM, *n* = 5, each with duplicate determinations) show Ca^{2+} released by IP_3 in the presence of the indicated concentrations of dequalinium. ER Ca^{2+} content was measured 20 s after IP_3 addition.
(**H-J**) Similar analyses of the effects of heparin (10 mg/mL, **I**) or 2-APB (125 μM, **J**). Means of 2 replicates, typical of 3-5 experiments.
(**K**) Effects of dequalinium (50 μM), heparin (10 mg/mL) or 2-APB (125 μM) on Ca^{2+} uptake by the intracellular stores of permeabilized HEK-IP_3R1 cells. Mean ± SD of 4-6 replicates, typical of 4 experiments.
(**L, M**) Summary results show the effects of antagonists on the steady-state Ca^{2+} content of the stores (**L**) and the rate of Ca^{2+} uptake (**M**). ER Ca^{2+} content was measured 180 s after addition of ATP (**L**). Mean ± SEM, *n* = 4, each with 4-6 determinations. $^{****}P < 0.0001$, $^{***}P < 0.001$, $^{*}P < 0.05$ one-way ANOVA with Bonferroni's test relative to control.

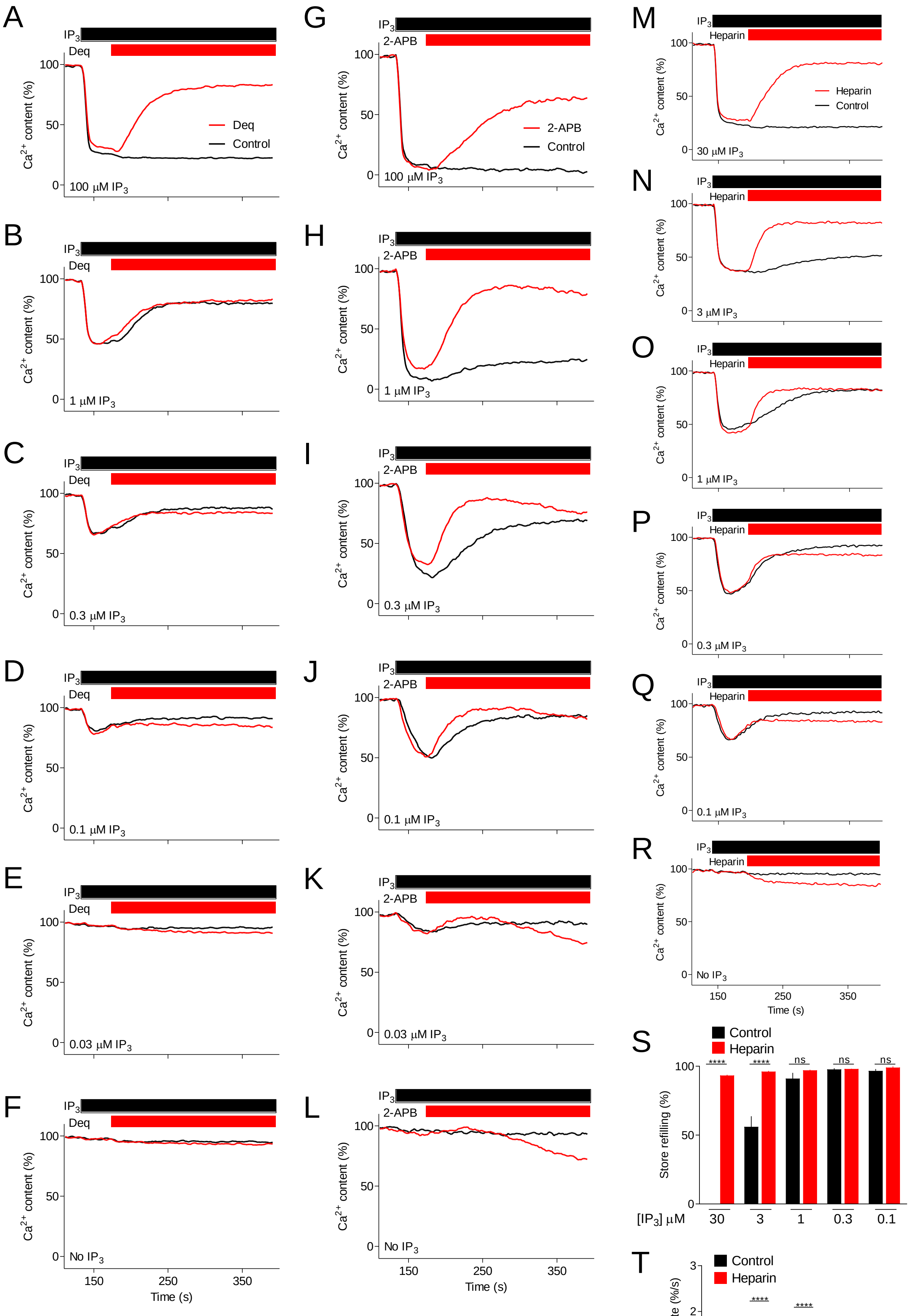

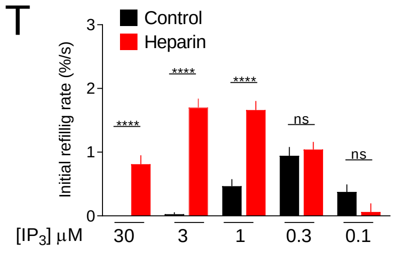

Figure S7. Blocking IP_3Rs with antagonists reveals that IP_3Rs close after incremental responses to low IP_3 concentrations

Legend on next page

Figure S7. Blocking IP_3Rs with antagonists reveals that IP_3Rs close after incremental responses to low IP_3 concentrations
Related to Figures 5 and 6.

Figure on preceding page

(**A-L**) Experiments similar to those shown in **Figure 5** were used to assess whether IP_3Rs remain open during sustained stimulation with the indicated concentrations of IP_3 using dequalinium (50 μM, **A-F**) or 2-APB (125 μM, **G-L**) as the IP_3R antagonist. Traces show means of 2 replicates. Summary results are shown in **Figures 5J** and **5K**.
(**M-R**) Experiments similar to those in **Figure 5** were repeated using wild-type HEK cells. Permeabilized cells were loaded with Ca^{2+} by addition of ATP before addition of the indicated concentrations of IP_3 (without inhibiting SERCA) and then the IP_3R antagonist, heparin (10 mg/mL). Traces show means of 2 replicates.
(**S, T**) Summary (mean ± SEM, $n = 3$, each with 2 determinations) show the extent to which stores refill (relative to cells without IP_3) 250 s after IP_3 addition (**S,** 0% refilling is defined as the content measured 250 s after addition of 30 μM IP_3 alone), and the initial rate of refilling from the slope of the curve immediately after heparin addition (**T**). $^{****}P < 0.0001$, one-way ANOVA with Bonferroni's test.

Methods S 1. Summary of Statistical Analyses. Related to STAR Methods.
The table summarises the results of all statistical analyses reported. $^{*}P < 0.05$, $^{**}P < 0.01$, $^{***}P < 0.001$, $^{****}P < 0.0001$.

**Figure**	**Test**	***P* values and summary**			
**Figure 1I**	Repeated measures one-way ANOVA.	P = 0.2594 (ns), ANOVA provides no justification for Bonferroni's test.			
**Figure 1J**	Repeated measures one-way ANOVA	P = 0.2021 (ns), ANOVA provides no justification for Bonferroni's test.			
**Figure 1M**	Ordinary one-way ANOVA with Bonferroni's multiple comparisons test	$P < 0.0001$			
		Bonferroni's test for comparisons of Ca^{2+} release recorded at 400 s versus 700 s for each of the four concentrations of CCh.			
			Adjusted P	$P < 0.05$	Summary
		1000µM CCh, 400 s vs 700 s	>0.9999	No	ns
		3µM CCh, 400 s vs 700 s	>0.9999	No	ns
		1.5µM CCh, 400 s vs 700 s	>0.9999	No	ns
		1µM CCh, 400 s vs 700 s	>0.9999	No	ns
**Figure 1O**	Repeated measures one-way ANOVA with Bonferroni's multiple comparisons test	$P < 0.0001$			
		Bonferroni's test for all comparisons			
			Adjusted P	$P < 0.05$	Summary
		1µM /3µM vs 0µM /3µM	0.1925	No	ns
		1µM /3µM vs 1µM /1000µM	0.0009	Yes	***
		1µM /3µM vs 3µM /1000µM	0.0011	Yes	**
		0µM /3µM vs 1µM /1000µM	0.0002	Yes	***
		0µM /3µM vs 3µM /1000µM	0.0002	Yes	***
		1µM /1000µM vs 3µM /1000µM	>0.9999	No	ns
**Figure 2E**	Ordinary one-way ANOVA with Bonferroni's multiple comparisons test	P = 0.0305			
		Bonferroni's test for all comparisons			
			Adjusted P	$P < 0.05$	Summary
		10µM IP$_3$ vs 10µM **2**	<0.0001	Yes	****
		10µM IP$_3$ vs 100µM **2**	<0.0001	Yes	****
		10µM IP$_3$ vs 10µM IP$_3$ with10µM **2**	<0.0001	Yes	****
		10µM **2** vs 100µM **2**	>0.9999	No	ns
		10µM **2** vs 10µM IP$_3$ with10µM **2**	0.0170	Yes	*
		100µM **2** vs 10µM IP$_3$ with10µM **2**	0.0111	Yes	*

**Figure 3D**	Repeated measures one-way ANOVA with Bonferroni's multiple comparisons test	$P < 0.0001$<br>Bonferroni's test for all comparisons			
			Adjusted *P*	$P < 0.05$	Summary
		0.1/0.3/1 vs 0/0.1/1	0.2683	No	ns
		0.1/0.3/1 vs 0/0.3/1	2.353	No	ns
		0.1/0.3/1 vs 0/0/1	-1.392	No	ns
		0.1/0.3/1 vs 0/0/100	-21.07	Yes	***
		0/0.1/1 vs 0/0.3/1	2.084	No	ns
		0/0.1/1 vs 0/0/1	-1.660	No	ns
		0/0.1/1 vs 0/0/100	-21.34	Yes	***
		0/0.3/1 vs 0/0/1	-3.745	No	ns
		0/0.3/1 vs 0/0/100	-23.42	Yes	***
		0/0/1 vs 0/0/100	-19.68	Yes	***

**Figure 3F**	Repeated measures one-way ANOVA with Bonferroni's multiple comparisons test	*P* < 0.0001<br>Bonferroni's test for all comparisons<br>Adjusted *P* / *P* < 0.05 / Summary<br>0µM **2**/ 100µM IP_3 vs 0µM **2**/ 300µM IP_3 — >0.9999 — No — ns<br>0µM **2**/ 100µM IP_3 vs 30µM **2**/ 0µM IP_3 — <0.0001 — Yes — ****<br>0µM **2**/ 100µM IP_3 vs 100µM **2** — 0.0002 — Yes — ***<br>0µM **2**/ 100µM IP_3 vs 30µM **2**/ 100µM IP_3 — >0.9999 — No — ns<br>0µM **2**/ 100µM IP_3 vs 30µM **2**/ 300µM IP_3 — 0.3147 — No — ns<br>0µM **2**/ 300µM IP_3 vs 30µM **2**/ 0µM IP_3 — <0.0001 — Yes — ****<br>0µM **2**/ 300µM IP_3 vs 100µM **2** — 0.0007 — Yes — ***<br>0µM **2**/ 300µM IP_3 vs 30µM **2**/ 100µM IP_3 — >0.9999 — No — ns<br>0µM **2**/ 300µM IP_3 vs 30µM **2**/ 300µM IP_3 — >0.9999 — No — ns<br>30µM **2**/ 0µM IP_3 vs 100µM **2** — >0.9999 — No — ns<br>30µM **2**/ 0µM IP_3 vs 30µM **2**/ 100µM IP_3 — 0.0003 — Yes — ***<br>30µM **2** / 0µM IP_3 vs 30µM **2**/ 300µM IP_3 — 0.0034 — Yes — **<br>100µM **2** vs 30µM **2**/ 100µM IP_3 — 0.0044 — Yes — **<br>100µM **2** vs 30µM **2**/ 300µM IP_3 — 0.0513 — No — ns<br>30µM **2**/ 100µM IP_3 vs 30µM **2**/ 300µM IP_3 — >0.9999 — No — ns
**Figure 5H**	Repeated measures one-way ANOVA with Bonferroni's multiple comparisons test	*P* < 0.0001<br>Bonferroni's test for only the indicated comparisons<br>*Adjusted P* / *P* < 0.05 / Summary<br>100 control vs 100 with heparin — <0.0001 — Yes — ****<br>10 control vs 10 with heparin — <0.0001 — Yes — ****<br>1 control vs 1with heparin — <0.0001 — Yes — ****<br>0.3 control vs 0.3 with heparin — <0.0001 — Yes — ****<br>0.1 control vs 0.1 with heparin — 0.4821 — No — ns<br>0.03 control vs 0.03 with heparin — >0.9999 — No — ns
**Figure 5I**	Repeated measures one-way ANOVA with Bonferroni's multiple comparisons test	*P* < 0.0001<br>Bonferroni's test for only the indicated comparisons<br>Adjusted *P* / *P* < 0.05 / Summary<br>100 control vs 100 with heparin — <0.0001 — Yes — ****<br>10 control vs 10 with heparin — <0.0001 — Yes — ****<br>1 control vs 1with heparin — <0.0001 — Yes — ****<br>0.3 control vs 0.3 with heparin — <0.0001 — Yes — ****<br>0.1 control vs 0.1 with heparin — >0.9999 — No — ns<br>0.03 control vs 0.03 with heparin — >0.9999 — No — ns

**Figure 5J**	Repeated measures one-way ANOVA with Bonferroni's multiple comparisons test	$P < 0.0001$<br>Bonferroni's test for only the indicated comparisons			
			Adjusted *P*	*P* < 0.05	Summary
		100 Control vs 100 with dequalinium	<0.0001	Yes	****
		10 Control vs 10 with dequalinium	<0.0001	Yes	****
		1 Control vs 1 with dequalinium	0.0003	Yes	***
		0.3 Control vs 0.3 with dequalinium	0.9947	No	ns
		0.1 Control vs 0.1 with dequalinium	0.9827	No	ns
		0.03 Control vs 0.03 with dequalinium	>0.9999	No	ns
**Figure 5K**	Repeated measures one-way ANOVA with Bonferroni's multiple comparisons test	$P < 0.0001$<br>Bonferroni's test for only the indicated comparisons			
			Adjusted *P*	*P* < 0.05	Summary
		100 Control vs 100 with 2-APB	<0.0001	Yes	****
		10 Control vs 10 with 2-APB	<0.0001	Yes	****
		1 Control vs 1 with 2-APB	<0.0001	Yes	****
		0.3 Control vs 0.3 with 2-APB	0.0151	Yes	*
		0.1 Control vs 0.1 with 2-APB	0.5176	No	ns
		0.03 Control vs 0.03 with 2-APB	0.9995	No	ns
**Figure 6G**	One-way ANOVA with Bonferroni's multiple comparisons test	$P < 0.0001$<br>Bonferroni's test for comparisons between control and dequalinium (DQ) for each concentration (µM) of (2,4,5)IP_3			
			Adjusted *P*	P < 0.05	Summary
		10 Control vs 10 with DQ	<0.0001	Yes	****
		3Control vs 3 with DQ	<0.0001	Yes	****
		1.5 Control vs 1.5 with DQ	0.4673	No	ns
		1 Control vs 1 with DQ	>0.9999	No	ns
		0.5 Control vs 0.5 with DQ	0.9815	No	ns
**Figure 6H**	One-way ANOVA with Bonferroni's multiple comparisons test	$P < 0.0001$<br>Bonferroni's test for comparisons between control and dequalinium (DQ) for each concentration (µM) of (2,4,5)IP_3			
			Adjusted *P*	P < 0.05	Summary
		10 Control vs 10 with DQ	<0.0001	Yes	****
		3Control vs 3 with DQ	<0.0001	Yes	****
		1.5 Control vs 1.5 with DQ	0.0146	Yes	*
		1 Control vs 1 with DQ	0.9766	No	ns
		0.5 Control vs 0.5 with DQ	>0.9999	No	ns

**Figure S2D**	Repeated measures one-way ANOVA.	*P* = 0.7935 (ns), ANOVA provides no justification for Bonferroni’s test.			
**Figure S2E**	Repeated measures one-way ANOVA.	*P* = 0.5315 (ns), ANOVA provides no justification for Bonferroni’s test.			
**Figure S2F**	Repeated measures one-way ANOVA.	*P* = 0.2898 (ns), ANOVA provides no justification for Bonferroni’s test.			
**Figure S2I**	Repeated measures one-way ANOVA with Bonferroni's multiple comparisons test	*P* < 0.0001<br>Bonferroni’s test for all comparisons			
			Adjusted *P*	*P* < 0.05	Summary
		0.01/0.015/0.03 vs 0/0.01/0.03	>0.9999	No	ns
		0.01/0.015/0.03 vs 0/0.015/0.03	>0.9999	No	ns
		0.01/0.015/0.03 vs 0/0/0.03	>0.9999	No	ns
		0.01/0.015/0.03 vs 0/0/30	<0.0001	Yes	****
		0/0.01/0.03 vs 0/0.015/0.03	>0.9999	No	ns
		0/0.01/0.03 vs 0/0/0.03	>0.9999	No	ns
		0/0.01/0.03 vs 0/0/30	<0.0001	Yes	****
		0/0.015/0.03 vs 0/0/0.03	>0.9999	No	ns
		0/0.015/0.03 vs 0/0/30	<0.0001	Yes	****
		0/0/0.03 vs 0/0/30	<0.0001	Yes	****
**Figure S2J**	Repeated measures one-way ANOVA with Bonferroni's multiple comparisons test	*P* < 0.0001<br>Bonferroni’s test for all comparisons			
			Adjusted *P*	*P* < 0.05	Summary
		0.1/0.3/1 vs 0/0.1/1	>0.9999	No	ns
		0.1/0.3/1 vs 0/0.3/1	0.0374	Yes	*
		0.1/0.3/1 vs 0/0/1	0.9790	No	ns
		0.1/0.3/1 vs 0/0/100	0.0002	Yes	***
		0/0.1/1 vs 0/0.3/1	0.0764	No	ns
		0/0.1/1 vs 0/0/1	>0.9999	No	ns
		0/0.1/1 vs 0/0/100	0.0001	Yes	***
		0/0.3/1 vs 0/0/1	0.6215	No	ns
		0/0.3/1 vs 0/0/100	<0.0001	Yes	****
		0/0/1 vs 0/0/100	<0.0001	Yes	****

**Figure S3H**	Repeated measures one-way ANOVA with Bonferroni's multiple comparisons test	*P* = 0.0416<br>Bonferroni's test for all comparisons<br>Adjusted *P* / *P* < 0.05 / Summary<br>0.03/0.1/0.3 vs 0.03/0/0.3: 0.0578, No, ns<br>0.03/0.1/0.3 vs 0/0.1/0.3: >0.9999, No, ns<br>0.03/0.1/0.3 vs 0/0/0.3: 0.2652, No, ns<br>0.03/0/0.3 vs 0/0.1/0.3: 0.2946, No, ns<br>0.03/0/0.3 vs 0/0/0.3: >0.9999, No, ns<br>0/0.1/0.3 vs 0/0/0.3: >0.9999, No, ns
**Figure S3I**	Repeated measures one-way ANOVA with Bonferroni's multiple comparisons test	*P* = 0.0468<br>Bonferroni's test for all comparisons<br>Adjusted *P* / *P* < 0.05 / Summary<br>0.03/0.1/10 vs 0.03/0/10: 0.5500, No, ns<br>0.03/0.1/10 vs 0/0.1/10: >0.9999, No, ns<br>0.03/0.1/10 vs 0/0/10: >0.9999, No, ns<br>0.03/0/10 vs 0/0.1/10: 0.1050, No, ns<br>0.03/0/10 vs 0/0/10: 0.0809, No, ns<br>0/0.1/10 vs 0/0/10: >0.9999, No, ns
**Figure S4G**	Repeated measures one-way ANOVA	*P* = 0.4676 (ns), ANOVA provides no justification for Bonferroni's test.
**Figure S4H**	One-way repeated ANOVA with Bonferroni's multiple comparisons test	*P* = 0.0305<br>Bonferroni's test for all comparisons<br>Adjusted *P* / *P* < 0.05 / Summary<br>0.05/0.1/10 vs 0/0.05/10: >0.9999, No, ns<br>0.05/0.1/10 vs 0/0.1/10: 0.7900, No, ns<br>0.05/0.1/10 vs 0/0/10: 0.0450, Yes, *<br>0/0.05/10 vs 0/0.1/10: >0.9999, No, ns<br>0/0.05/10 vs 0/0/10: 0.0051, Yes, **<br>0/0.1/10 vs 0/0/10: 0.2752, No, ns
**Figure S5E**	One-way ANOVA with Bonferroni's multiple comparisons test	*P* = 0.0027<br>Bonferroni's test for comparisons with DMSO control<br>Adjusted *P* / *P* < 0.05 / Summary<br>Control vs ML-SA1 with FCCP: 0.0016, Yes, **<br>Contro vs ML-SA1 with Bafilomycin A_1: 0.0041, Yes, **<br>Control vs ML-SA1 with DMSO: 0.0099, Yes, **

**Figure S5I**	Repeated measures one-way ANOVA with Bonferroni's multiple comparisons test	*P* < 0.0001<br>Bonferroni's test for all comparisons	Adjusted *P*	*P* < 0.05	Summary
		ns vs erlin 2 (1)	0.0007	Yes	***
		ns vs erlin 2 (2)	<0.0001	Yes	***
		erlin 2 (1) vs erlin 2 (2)	0.0594	No	ns
**Figure S6B**	Paired, two-tailed Student's *t*-test	*P* = 0.1411 (ns)			
**Figure S6L**	Repeated measures one-way ANOVA with Bonferroni's multiple comparisons test	*P* = 0.0132<br>Bonferroni's test for comparisons relative to control	Adjusted *P*	*P* < 0.05	Summary
		Control vs 2-APB	0.0561	No	ns
		Control vs heparin	0.0056	Yes	**
		Control vs dequalinium	0.1510	No	ns
**Figure S6M**	Repeated measures one-way ANOVA with Bonferroni's multiple comparisons test	*P* < 0.0001<br>Bonferroni's test for comparisons relative to control	Adjusted *P*	*P* < 0.05	Summary
		Control vs 2-APB	0.9086	No	ns
		Control vs heparin	<0.0001	Yes	****
		Control vs dequalinium	<0.0163	Yes	*
**Figure S7S**	Repeated measures one-way ANOVA with Bonferroni's multiple comparisons test	*P* < 0.0001<br>Bonferroni's test for only the indicated comparisons	Adjusted *P*	*P* < 0.05	Summary
		30 Control vs 30 with heparin	<0.0001	Yes	****
		3 Control vs 3 with heparin	<0.0001	Yes	****
		1 Control vs 1 with heparin	0.4804	No	ns
		0.3 Control vs 0.3 with heparin	>0.9999	No	ns
		0.1 Control vs 0.1 with heparin	0.9753	No	ns
**Figure S7T**	Repeated measures one-way ANOVA with Bonferroni's multiple comparisons test	*P* < 0.0001<br>Bonferroni's test for only the indicated comparisons	Adjusted *P*	P < 0.05	Summary
		30 Control vs 30 with heparin	<0.0001	Yes	****
		3 Control vs 3 with heparin	<0.0001	Yes	****
		1 Control vs 1 with heparin	<0.0001	Yes	****
		0.3 Control vs 0.3 with heparin	0.9733	No	ns
		0.1 Control vs 0.1 with heparin	0.1990	No	ns

**Table S1**	Unpaired, 2-tailed Student's *t*-test.	All comparisons are for IP_3 vs **2**<br>Binding (pK_D)<br>IP_3R1: $P = 0.0002$ (***)<br>NT: $P = 0.2873$ (ns)<br>IBC: $P = 0.0023$ (**)<br>Ca^{2+} release<br>Maximal release: $P < 0.001$ (***)<br>pEC_{50}: $P = 0.0120$ (*)<br>Electrophysiology<br>NP_o: $P < 0.0001$ (****)<br>γ_K: $P = 0.7485$ (ns)<br>τ_o: $P < 0.0001$ (****)
**Table S2**	Unpaired, two-tailed Student's *t*-test	pEC_{50}: $P = 0.0354$ (*)<br>Ca^{2+} release: $P = 0.0279$ (*)<br>h: $P = 0.4855$ (ns)
**Table S3**	Unpaired, 2-tailed Student's *t*-test.	Comparisons of Ca^{2+} release evoked by IP_3 alone or after incubation with **2**<br>pEC_{50}: $P < 0.001$ (***)<br>Ca^{2+} release: $P = 0.1681$ (ns)
**Table S4**	Paired two-tailed Student's *t*-test	pEC_{50}: $P = 0.9565$ (ns)<br>Ca^{2+} release: $P = 0.8442$ (ns)<br>h: $P = 0.3110$ (ns)
