# Supplementary material for: Quantal Ca2+ release mediated by very few IP3 receptors that rapidly inactivate allows graded responses to IP3
Source: Cell Rep. 2021 Nov 2;37(5):109932. doi: 10.1016/j.celrep.2021.109932 (PMC8578705; doi:10.1016/j.celrep.2021.109932)
Supplement: Document S2. Article plus supplemental information [file mmc2.pdf]

Cell Reports

Article

Quantal Ca^{2+} release mediated by very few IP_3 receptors that rapidly inactivate allows graded responses to IP_3

Graphical abstract

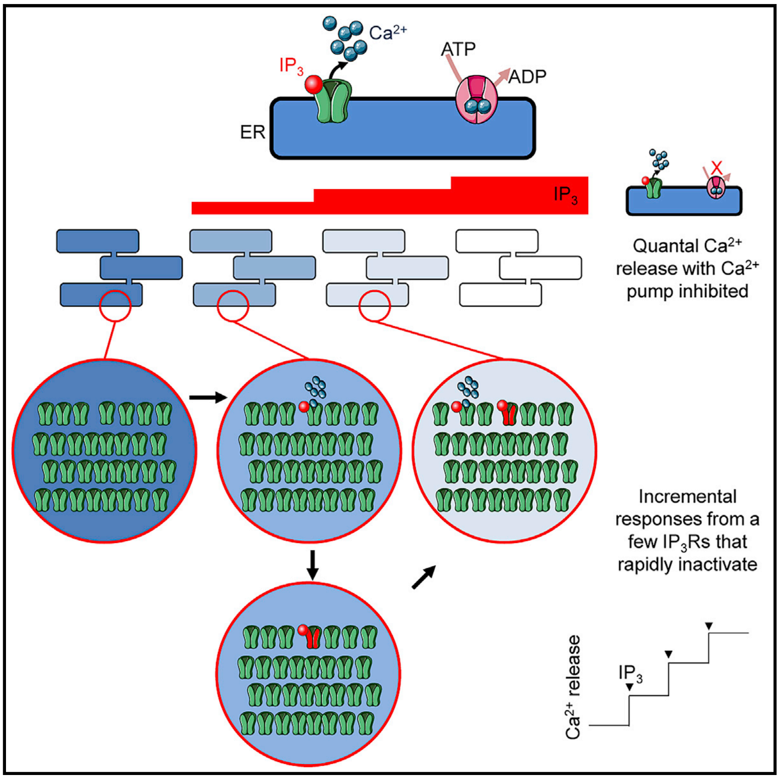

Authors

Ana M. Rossi, Andrew M. Riley, Geneviève Dupont, Taufiq Rahman, Barry V.L. Potter, Colin W. Taylor

Correspondence

amr50@cam.ac.uk (A.M.R.), cwt1000@cam.ac.uk (C.W.T.)

In brief

Rossi et al. define mechanisms for IP_3-evoked quantal Ca^{2+} release that reconcile response termination with undiminished sensitivity to further IP_3 additions. They show that responses require very few IP_3 receptors that rapidly open and then inactivate, allowing graded responses despite the ability of IP_3 receptors to propagate regenerative Ca^{2+} signals.

Highlights

- IP_3 evokes quantal Ca^{2+} release from the endoplasmic reticulum
- IP_3 receptors rapidly activate and then inactivate
- Submaximal responses to IP_3 require activation of very few IP_3 receptors
- Rapid activation and inactivation of very few IP_3Rs allow incremental responses to IP_3

Rossi et al., 2021, Cell Reports *37*, 109932
November 2, 2021 © 2021 The Author(s).
https://doi.org/10.1016/j.celrep.2021.109932

CellPress

Cell Reports

CellPress
OPEN ACCESS

Article

Quantal Ca^{2+} release mediated by very few IP_3 receptors that rapidly inactivate allows graded responses to IP_3

Ana M. Rossi,[1,*] Andrew M. Riley,[2] Geneviève Dupont,[3] Taufiq Rahman,[1] Barry V.L. Potter,[2] and Colin W. Taylor[1,4,*]
[1]Department of Pharmacology, University of Cambridge, Tennis Court Road, Cambridge CB2 1PD, UK
[2]Drug Discovery and Medicinal Chemistry, Department of Pharmacology, University of Oxford, Mansfield Road, Oxford OX1 3QT, UK
[3]Unité de Chronobiologie Théorique, Faculté des Sciences, CP231 Université Libre de Bruxelles (ULB), Boulevard du Triomphe, 1050 Brussels, Belgium
[4]Lead contact
*Correspondence: amr50@cam.ac.uk (A.M.R.), cwt1000@cam.ac.uk (C.W.T.)
https://doi.org/10.1016/j.celrep.2021.109932

SUMMARY

Inositol 1,4,5-trisphosphate receptors (IP_3Rs) are intracellular Ca^{2+} channels that link extracellular stimuli to Ca^{2+} signals. Ca^{2+} release from intracellular stores is "quantal": low IP_3 concentrations rapidly release a fraction of the stores. Ca^{2+} release then slows or terminates without compromising responses to further IP_3 additions. The mechanisms are unresolved. Here, we synthesize a high-affinity partial agonist of IP_3Rs and use it to demonstrate that quantal responses do not require heterogenous Ca^{2+} stores. IP_3Rs respond incrementally to IP_3 and close after the initial response to low IP_3 concentrations. Comparing functional responses with IP_3 binding shows that only a tiny fraction of a cell's IP_3Rs mediate incremental Ca^{2+} release; inactivation does not therefore affect most IP_3Rs. We conclude, and test by simulations, that Ca^{2+} signals evoked by IP_3 pulses arise from rapid activation and then inactivation of very few IP_3Rs. This allows IP_3Rs to behave as increment detectors mediating graded Ca^{2+} release.

INTRODUCTION

Inositol 1,4,5-trisphosphate (IP_3) links receptors in the plasma membrane to Ca^{2+} release from the endoplasmic reticulum (ER) through IP_3 receptors (IP_3Rs), which are intracellular Ca^{2+} channels (Berridge, 2016). This redistribution of Ca^{2+} from the ER generates cytosolic Ca^{2+} signals, transfers Ca^{2+} to other organelles, and stimulates store-operated Ca^{2+} entry. IP_3-evoked Ca^{2+} signals initiate at small clusters of IP_3Rs, where IP_3 binding primes IP_3Rs to open in response to Ca^{2+} released by their neighbors (Smith and Parker, 2009; Thillaiappan et al., 2017, 2021). Similar Ca^{2+}-induced Ca^{2+} release (CICR) occurs with ryanodine receptors (RyRs), the other major family of ER Ca^{2+} channels (Ríos, 2018). However, CICR is potentially explosive and might prevent cells from generating graded responses. Dispersed clusters of channels constrain regenerative propagation of Ca^{2+} signals between them (Ríos, 2018), and inhibition of IP_3Rs (and RyRs) by increased cytosolic free Ca^{2+} concentration ($[Ca^{2+}]_c$) may contribute to terminating Ca^{2+} release, but it may not be the only mechanism (Wiltgen et al., 2014).

IP_3-evoked Ca^{2+} release is "quantal": submaximal IP_3 concentrations rapidly release only a fraction of the Ca^{2+} stores before release terminates (Muallem et al., 1989; Taylor and Potter, 1990). This pattern of response occurs without compromising responses to further incremental increases in IP_3 concentration (Meyer and Stryer, 1990), indicating that it is not mediated by a conventional form of desensitization. Quantal Ca^{2+} release by IP_3Rs has been reported by many laboratories (reviewed in Bootman [1994] and Yamashita [2006]), and it is also a feature of RyRs (Cheek et al., 1994; Wang et al., 2004). Quantal responses have been most thoroughly examined in permeabilized cells, but they have also been reported in intact cells responding to stimuli that evoke IP_3 formation (Bootman et al., 1992; Muallem et al., 1989). Hence, quantal Ca^{2+} release is an essential feature of both major families of ER Ca^{2+} channels.

The mechanism of quantal Ca^{2+} release is unknown, but it is not due to IP_3 metabolism or compensatory Ca^{2+} re-uptake, nor does it require ATP (Meyer and Stryer, 1990; Taylor and Potter, 1990). Two categories of mechanism have been proposed. One suggests that submaximal concentrations of IP_3 completely empty the stores that are most sensitive to IP_3, and higher IP_3 concentrations then recruit the less sensitive stores (Ferris et al., 1992; Hirose and Iino, 1994; Muallem et al., 1989; Oldershaw et al., 1991; Parker and Ivorra, 1990) (Figure 1A). This model requires both extremely cooperative responses to IP_3 and compartmentalized Ca^{2+} stores with heterogenous sensitivities to IP_3. The second model suggests that IP_3R activity attenuates before Ca^{2+} stores are fully depleted (Figure 1B) (Irvine, 1990; Missiaen et al., 1992; Parys et al., 1993; Tregear et al., 1991). The mechanisms that might curtail IP_3R-mediated Ca^{2+} release include IP_3-mediated inactivation (Hajnóczky and Thomas, 1994), inhibition by increased $[Ca^{2+}]_c$, loss of the electrochemical Ca^{2+} gradient for Ca^{2+} release as Ca^{2+}

This is an open access article under the CC BY license (http://creativecommons.org/licenses/by/4.0/).

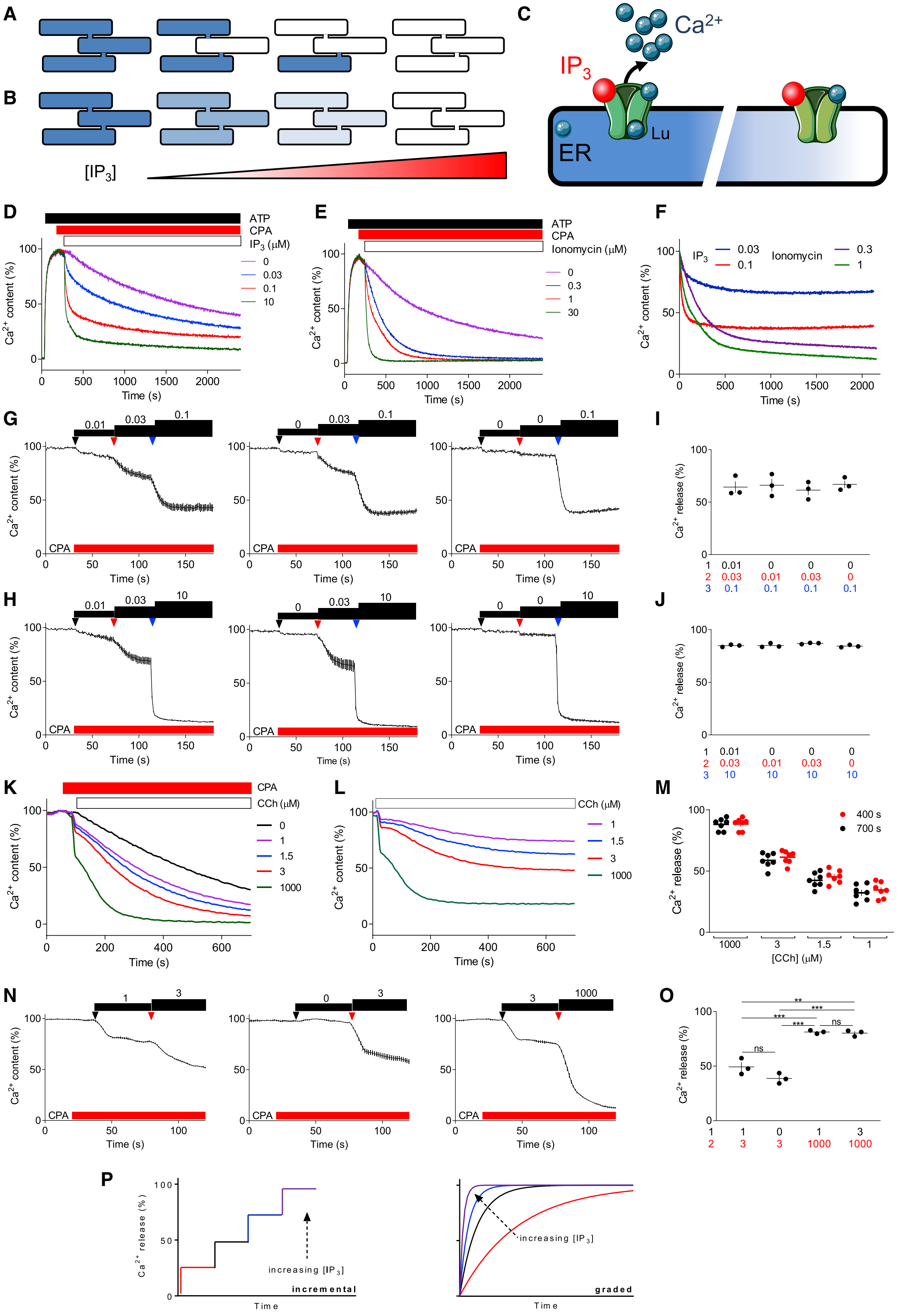

(legend on next page)

leaves the ER without compensatory charge movements (Yamashita et al., 2006), and regulation of IP_3Rs by luminal Ca^{2+} (Figure 1C). The latter mechanism proposes that opening of an IP_3R requires binding of IP_3 and of Ca^{2+} at both the cytosolic and luminal sides of the IP_3R (Irvine, 1990; Marchant and Taylor, 1997). Regulation by luminal Ca^{2+} predicts that as ER [Ca^{2+}] falls, the sensitivity of IP_3Rs to cytosolic IP_3 and Ca^{2+} declines, and Ca^{2+} release then terminates with Ca^{2+} trapped in the ER (Figure 1C). In the 30 years since quantal Ca^{2+} release was first identified (Muallem et al., 1989; Taylor and Potter, 1990), numerous studies have confirmed the phenomenon and provided evidence that supports or challenges each mechanism (Yamashita, 2006). Single-channel recordings from IP_3Rs (Ionescu et al., 2006; Taufiq-Ur-Rahman et al., 2009) and high-resolution optical methods (Callamaras and Parker, 2000) have also failed to identify the mechanisms underlying incremental responses to IP_3.

Here, we synthesize a high-affinity, partial agonist of IP_3R with exceptionally low efficacy, and use it to establish that quantal responses are not mediated by all-or-nothing emptying of stores with heterogenous sensitivities to IP_3 (Figure 1A). We show that all three subtypes of IP_3Rs mediate incremental responses to IP_3, and that the responses arise from a tiny fraction of a cell's IP_3Rs opening in response to IP_3 and then inactivating. This mechanism resolves long-standing confusion and establishes how cells respond to incremental changes in IP_3 concentration.

RESULTS AND DISCUSSION

Quantal Ca^{2+} release by all IP_3R subtypes

To measure IP_3-evoked Ca^{2+} release without the opposing activity of ER Ca^{2+} pumps (sarcoplasmic/ER Ca^{2+}-ATPases [SERCAs]), the ER of permeabilized DT40 cells expressing only IP_3R1 (DT40-IP_3R1 cells) was loaded with Mag-fluo 4, a low-affinity Ca^{2+} indicator (Rossi and Taylor, 2020). ATP was added to fuel Ca^{2+} uptake before addition of cyclopiazonic acid (CPA) to inhibit SERCAs; IP_3 was then added to stimulate Ca^{2+} release (Figure 1D). A maximally effective IP_3 concentration released ~70% of the ER Ca^{2+} content. Submaximal IP_3 concentrations rapidly released a smaller fraction of the stores, after which there was no further effect of IP_3 on the rate of Ca^{2+} release or its effect was much reduced (Figure 1D). These quantal responses to IP_3 are clearest after correction for the basal Ca^{2+} leak evident after SERCA inhibition (Figure 1F). The responses to IP_3 are very different to those evoked by the Ca^{2+} ionophore, ionomycin, which stimulated monophasic Ca^{2+} loss from the entire ER at rates that increased with ionomycin concentration (Figures 1E, 1F, S1A, and S1B). Similar quantal responses from type 1 IP_3R (IP_3R1) were observed with IP_3 in the presence of the K^+ ionophore, valinomycin, to dissipate any ER membrane potential; with Cs^+ replacing K^+ to inhibit K^+ channels; with mitochondria inhibited; with IP_3 added in larger volumes to avoid possible artifacts arising from bolus additions; and with inositol 2,4,5-trisphosphate ((2,4,5)IP_3), a non-metabolized analog of IP_3 (Hill et al., 1988) (Figures S1C–S1J).

Although responses to low IP_3 concentrations rapidly attenuated (Figures 1D, 1F, and S1B), subsequent addition of more IP_3 evoked further rapid Ca^{2+} release. Furthermore, the fraction of the Ca^{2+} stores released by maximal or submaximal IP_3 concentrations was the same whether IP_3 was delivered immediately at its final concentration or as incremental additions (Figures 1G–1J). Similar "incremental" responses (Beecroft and Taylor, 1997; Ferris et al., 1992; Meyer and Stryer, 1990) were obtained in the presence of the fast Ca^{2+} buffer, BAPTA, at a concentration (10 mM) sufficient to rapidly buffer even very local

Figure 1. Quantal Ca^{2+} release by IP_3Rs

(A–C) Proposed mechanisms for quantal Ca^{2+} release: all-or-nothing emptying of stores with different IP_3 sensitivities (A), or mechanisms that terminate Ca^{2+} release before stores have fully emptied (B). The latter could be mediated by luminal Ca^{2+} regulating IP_3Rs, such that as the luminal [Ca^{2+}] falls after IP_3R activation, Ca^{2+} dissociates from the luminal site (Lu), and IP_3Rs close trapping Ca^{2+} within the ER (C) (Irvine, 1990).

(D and E) Ca^{2+} content of the ER of permeabilized Mag-fluo 4-loaded DT40-IP_3R1 cells after addition of ATP (1.5 mM) followed by CPA (10 μM) to inhibit SERCAs, and then the indicated concentrations (μM) of IP_3 (D) or ionomycin (E). Results (typical of three to four experiments, each with two replicates) show Ca^{2+} content (%) relative to steady-state Ca^{2+} content; (D) is reproduced from Rossi and Taylor (2020).

(F) Responses to IP_3 and ionomycin plotted after subtraction of the basal Ca^{2+} leak.

(G and H) Effects of submaximal (100 nM) (G) or maximal (10 μM) (H) concentrations of IP_3 added directly or by incremental additions to permeabilized DT40-IP_3R1 cells. Results show mean ± SD of three replicates.

(I and J) Summary results (individual values, mean ± SEM, n = 3, each with three replicates) show the Ca^{2+} released from the stores of permeabilized DT40-IP_3R1 cells (determined at 150 s) for the three incremental additions (cumulative IP_3 concentrations shown in μM). The final Ca^{2+} release was not significantly different between any of the incremental additions (one-way repeated ANOVA with Bonferroni's multiple comparisons test).

(K) Ca^{2+} content of the ER of intact wild-type HEK expressing G-CEPIA1*er* after addition of CPA (5 μM) to inhibit SERCAs, and then the indicated concentrations of carbachol (CCh, μM). Results (typical of seven experiments, each with three replicates) show Ca^{2+} content (%) relative to the Ca^{2+} content determined before adding CPA.

(L) Responses to the indicated concentrations of carbachol in intact wild-type HEK expressing G-CEPIA1*er* after subtraction of the basal Ca^{2+} leak.

(M) Summary results (mean ± SEM, n = 7, each with three replicates) show the Ca^{2+} released from the stores of intact wild-type HEK expressing G-CEPIA1*er* determined at 400 and 700 s for the indicated concentrations of carbachol. The Ca^{2+} release at 400 and 700 s was not significantly different at any of the carbachol concentrations (one-way repeated ANOVA with Bonferroni's multiple comparisons test).

(N) Incremental responses from intact wild-type HEK cells to the indicated concentrations of carbachol added after CPA (5 μM) and recorded using G-CEPIA1*er* (cumulative carbachol concentrations are shown in μM).

(O) Summary results from intact wild-type HEK cells expressing G-CEPIA1*er* (individual values, mean ± SEM, n = 3, each with 8–12 replicates) show Ca^{2+} released from the stores (determined at 150 s) for the incremental additions (cumulative carbachol concentrations shown in μM). **$p < 0.01$, ***$p < 0.001$; ns, not significant (for indicated comparisons; one-way repeated ANOVA with Bonferroni's multiple comparisons test).

(P) Incremental responses to IP_3 compared with the graded rates of Ca^{2+} release expected if increasing concentrations of IP_3 uniformly increased the permeability of the ER to Ca^{2+}.

See also Figures S1–S4.

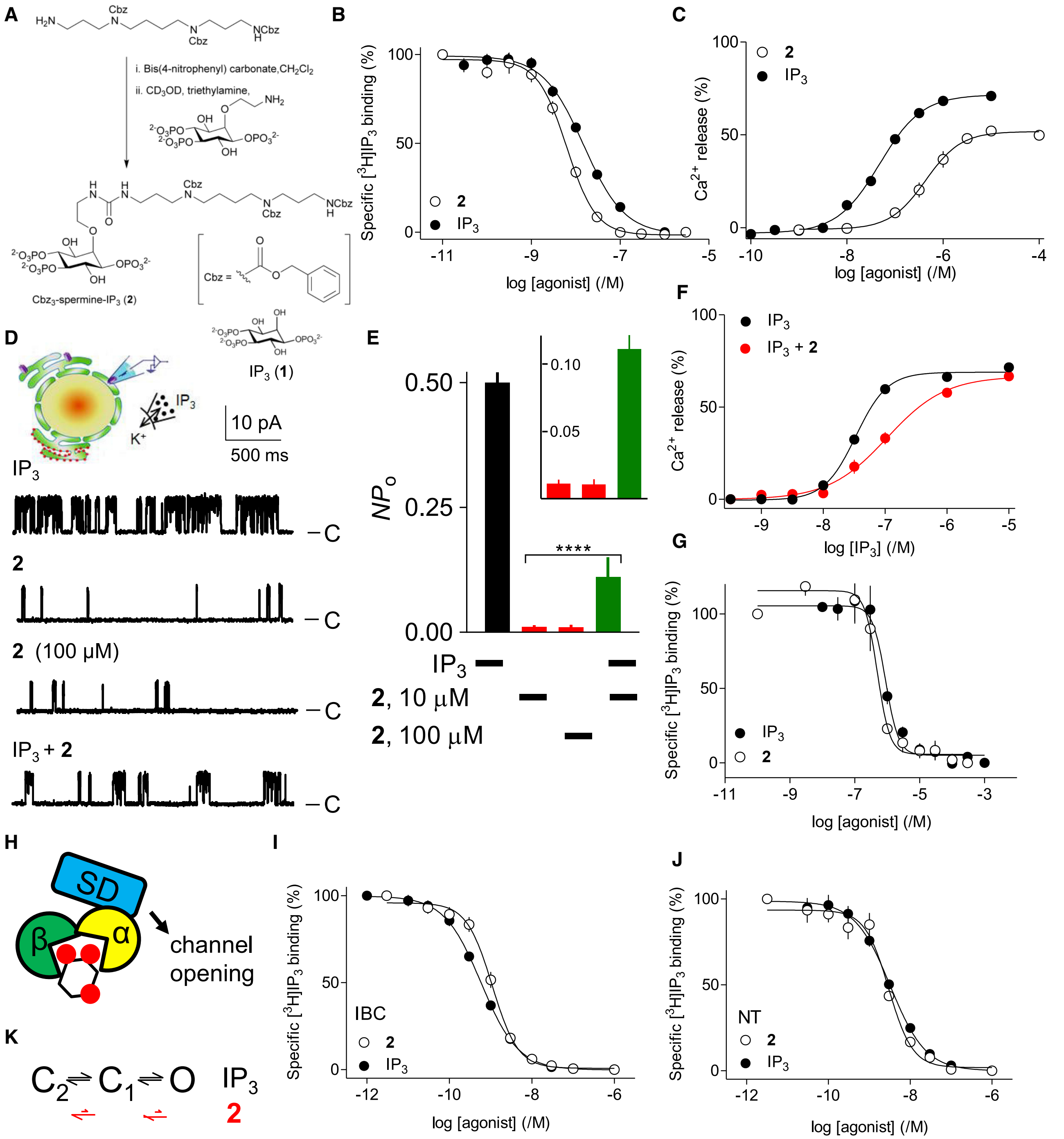

Figure 2. A 2-*O*-modified analog of IP_3 (2) is a high-affinity, partial agonist

(A) Synthetic scheme for **2** and structure of IP_3.

(B) Equilibrium-competition binding to purified full-length IP_3R1 using $[^3H]IP_3$ (1.5 nM) and the indicated concentrations of IP_3 or **2** in Tris-EDTA medium (TEM) at 4°C (mean ± SEM, n = 7, many error bars are smaller than the symbols).

(C) Effects of IP_3 or **2** on Ca^{2+} release (determined after 20 s) from permeabilized DT40-IP_3R1 cells (mean ± SEM, n = 3, each with three replicates).

(D) Patch-clamp recordings, typical of at least five recordings, from excised nuclear patches of DT40-IP_3R1 cells, at a holding potential of +40 mV with K^+ as the charge carrier, and ligands within the pipette (cytosolic surface; IP_3, 10 μM; **2**, 10 μM, except where shown otherwise). C, closed state.

(E) Summary results (mean ± SEM, n = 5–7) show channel activity (NP_o) for the indicated stimuli (enlarged in the inset). ****p < 0.0001, ANOVA with Bonferroni's test, relative to IP_3 alone.

(legend continued on next page)

increases in $[Ca^{2+}]_c$ (Vais et al., 2012). This indicates that inhibition of IP_3Rs by increases in $[Ca^{2+}]_c$ are not required for incremental responses (Figures S2A–S2F). That conclusion is consistent with two additional lines of evidence. First, IP_3Rs from *Capsaspora owczarzaki* are not regulated by cytosolic Ca^{2+}, but they do mediate quantal Ca^{2+} release (Alzayady et al., 2015). Second, in ER depleted of Ca^{2+}, retrograde movement of Mn^{2+} through open IP_3Rs can be used to report IP_3R opening. Under these conditions, responses to IP_3 have been reported to be quantal, but only after fragmentation of the ER (Hajnóczky et al., 1994; Renard-Rooney et al., 1993). Responses were also incremental in permeabilized DT40 cells expressing only IP_3R2 or IP_3R3 (Figures S2G–S2J), and in wild-type human embryonic kidney (HEK) cells, which express all three IP_3R subtypes (Mataragka and Taylor, 2018) (Figure S3). In each case, the total amount of Ca^{2+} released by a submaximal concentration of IP_3 was the same whether IP_3 was presented as a single addition or as incremental additions. Incremental responses to IP_3 were also observed using a low-affinity genetically encoded Ca^{2+} indicator targeted to the ER lumen (G-CEPIA1*er*) (Suzuki et al., 2014) (Figures S4A–S4H).

In single-cell analyses of permeabilized HEK cells stably expressing G-CEPIA1*er*, all cells responded to a maximal IP_3 concentration with a rapid decrease in ER luminal $[Ca^{2+}]$. Submaximal IP_3 concentrations rapidly released a fraction of the IP_3-sensitive stores, and the response then terminated without preventing subsequent responses to a maximal IP_3 concentration (Figures S4I–S4N). Quantal responses observed in populations of permeabilized cells are not, therefore, due to all-or-nothing responses from different cells with heterogeneous IP_3 sensitivities. In permeabilized hepatocytes too, quantal responses to IP_3 were observed in single cells (Renard-Rooney et al., 1993). We also measured changes in G-CEPIA1*er* fluorescence within small subcellular regions and, although the spatial resolution was limited, the results suggest that different regions within a HEK cell have similar sensitivity to IP_3 (Figures S4K–S4N).

It has been reported that quantal responses to IP_3 are observed in permeabilized hepatocytes only when permeabilization is accompanied by fragmentation of the ER (Renard-Rooney et al., 1993). Most studies do not address whether the ER remains continuous after permeabilization. In our analyses of permeabilized HEK cells, there is some fragmentation of the ER, whether reported by G-CEPIA1*er* or compartmentalized Mag-fluo 4, and attempts to avoid it by adjusting conditions failed to achieve ATP-dependent Ca^{2+} uptake without some ER fragmentation (Figure S4O). However, several lines of evidence establish that quantal responses are not an artifact arising from ER fragmentation. First, others have observed quantal responses from stimuli that evoke IP_3 formation in intact cells (Bootman et al., 1992; Muallem et al., 1989). By measuring quantal responses directly using a Ca^{2+} indicator within the ER lumen, we confirmed that stimulation of the endogenous muscarinic acetylcholine receptors of HEK cells with carbachol to evoke IP_3 formation caused quantal Ca^{2+} release from intracellular stores (Figures 1K–1M). Successive additions of carbachol evoked incremental responses (Figures 1N and 1O). Second, fragmentation of the ER might create the heterogenous Ca^{2+} stores required for the all-or-nothing model of quantal Ca^{2+} release (Figure 1A), but it is difficult to envisage how it might create the conditions required for a model where IP_3Rs close before the stores have fully emptied (Figure 1B). Our subsequent experiments demonstrate that all-or-nothing emptying of discrete Ca^{2+} stores cannot explain quantal responses (see Figures 2 and 3), and we provide direct evidence that IP_3Rs inactivate before the stores have emptied (see Figures 5, 6, and 7).

The results so far confirm that incremental responses to IP_3 occur within single cells, they are not a consequence of cell permeabilization, and they are not mediated by increases in $[Ca^{2+}]_c$, by IP_3 metabolism, or by ineffective movement of counter-ions, nor do mitochondria contribute. Incremental responses to IP_3 (Figure 1P) are a feature of all IP_3R subtypes, and genetically encoded IP_3R heterogeneity is not required for quantal Ca^{2+} release. The present results do not, however, distinguish between the proposed mechanisms (Figures 1A–1C).

Characterization of a high-affinity, weak partial agonist of IP_3Rs

Because partial agonists activate receptors less effectively than do full agonists (Rossi et al., 2009), we predicted that if a weak partial agonist occupied all IP_3Rs and evoked quantal Ca^{2+} release, the quantal mechanism could not be due to heterogenous IP_3R sensitivity. We previously developed 2-*O*-modified IP_3 analogs that are partial agonists (Rossi et al., 2009), but none has sufficiently low efficacy for our present needs. Reasoning that enlarging the 2-*O*-substituent might further reduce efficacy, we synthesized additional analogs and found Cbz_3-spermine-IP_3 (compound **2**) to be an exceptionally weak partial agonist (Figure 2A). Compound **2** bound to IP_3R1 with about 2-fold greater affinity than IP_3 (Figure 2B), but it was significantly less potent than IP_3 in stimulating Ca^{2+} release from permeabilized DT40-IP_3R1 cells (Figures 2C; Table S1) or wild-type HEK cells (Table S2). The difference between binding and functional analyses is captured by comparing the ligand concentrations required to release 50% of the IP_3-sensitive Ca^{2+} stores (EC^{I}_{50}) and to occupy 50% of IP_3-binding sites (K_D, equilibrium

(F) Permeabilized DT40-IP_3R1 cells stimulated (30 s) with **2** (1 μM, which evoked 31% ± 7% Ca^{2+} release) were then stimulated with the indicated concentrations of IP_3. Ca^{2+} release evoked by IP_3 (determined after a further 20 s) is shown as a percentage of the store content after the first stimulus (mean ± SEM, n ≥ 4, each with three replicates).

(G) Equilibrium-competition binding to cerebellar membranes using $[^3H]IP_3$ (7.5 nM) and the indicated concentrations of IP_3 or **2** in CLM at 20°C (mean ± SEM, n = 3–4 with duplicate determinations). pK_D values were 6.10 ± 0.06 (mean ± SEM, n = 4, K_D = 794 nM) for IP_3 and 6.26 ± 0.01 (n = 3, K_D = 549 nM) for **2**. Hill coefficients (*h*) were 3.2 ± 1.3 and 2.0 ± 0.7 for IP_3 and **2**.

(H) IP_3 binds between the α and β domains of the IBC, but the SD is required for IP_3 to evoke channel opening.

(I and J) Equilibrium-competition binding of IP_3 and **2** to the IBC or NT using $[^3H]IP_3$ (0.3 or 0.75 nM for IBC and NT, respectively). Mean ± SEM, n = 4.

(K) IP_3R moves between an unknown numbers of closed states (C) to an open state (O) after IP_3 binding. Rate(s) of movement through the closed to the open state occur more slowly with **2** bound to the IP_3R.

Tables S1–S3 summarize the properties of IP_3 and **2**.

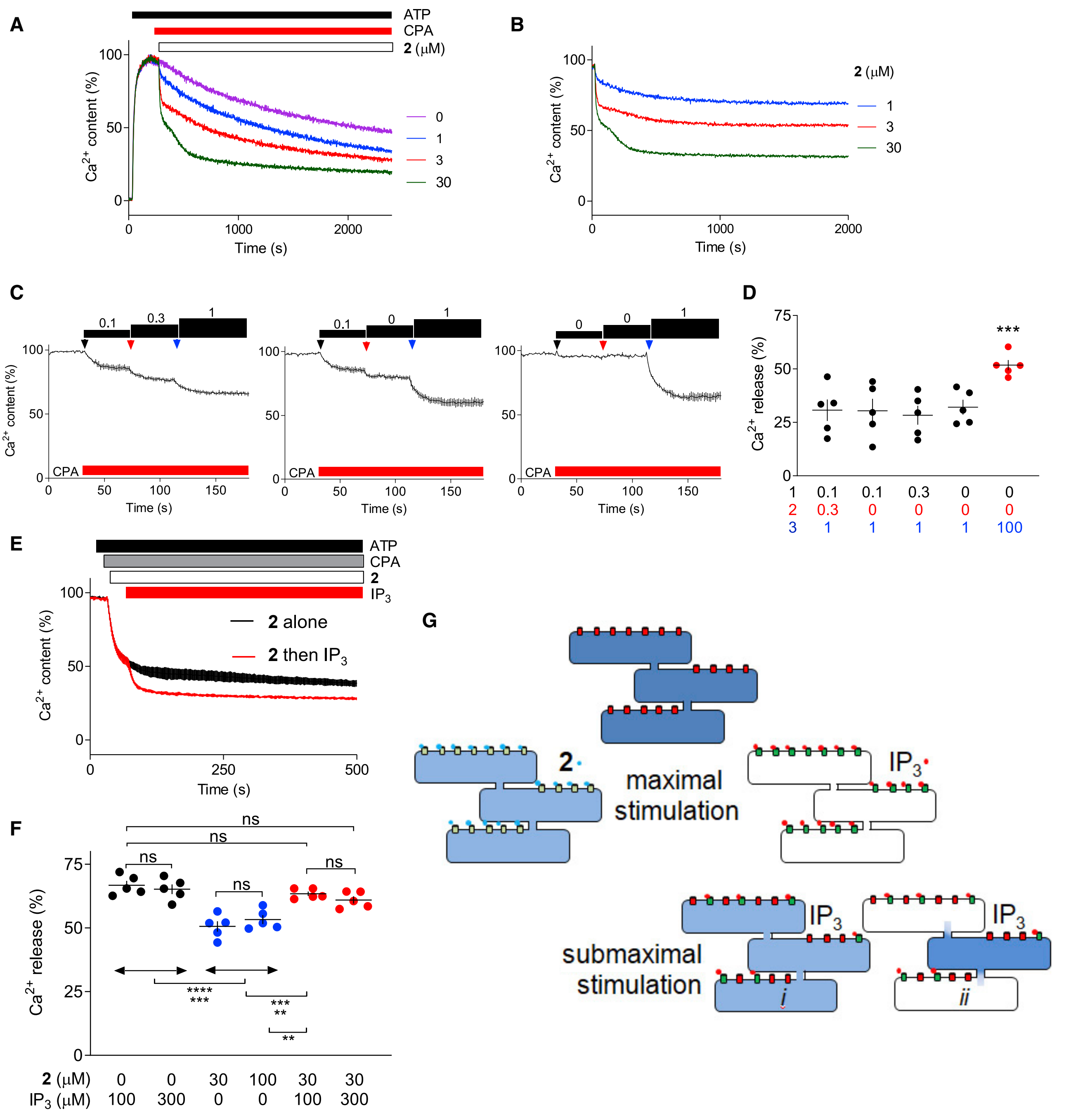

Figure 3. Quantal responses are not mediated by heterogenous stores

(A) Ca^{2+} content of the ER of populations of permeabilized DT40-IP_3R1 cells after addition of ATP, CPA (10 μM), and then the indicated concentrations of **2**. Results are typical of three experiments, each with duplicate determinations.

(B) Responses to **2** shown after subtraction of the basal Ca^{2+} leak.

(C) Effects of a submaximal concentration of **2** (1 μM) added directly or by incremental additions. Results show mean ± SD of three replicates.

(D) Summary results (individual values, mean ± SEM, n = 5, each with three replicates) show the final Ca^{2+} content of the stores of permeabilized DT40-IP_3R1 cells (determined at 150 s). A maximal concentration of **2** (30 μM) released 52% ± 3% of the Ca^{2+} stores (red symbols). ***$p < 0.001$, one-way repeated ANOVA with Bonferroni's test relative to all other values.

(E) Ca^{2+} release from wild-type HEK cells evoked by **2** (30 μM) alone or followed by a supra-maximal concentration of IP_3 (100 μM). Mean of duplicate determinations.

(legend continued on next page)

dissociation constant); the ratio (EC^{I}_{50}/K_D) is 41-fold greater for **2** than for IP_3 (Table S1). The weakest known partial agonist with an affinity comparable to IP_3 (compound 4 in Rossi et al., 2009) has an EC^{I}_{50}/K_D ratio only 5.7-fold greater than that of IP_3.

In patch-clamp recordings from the outer nuclear envelope, which is continuous with the ER, the increase in channel open probability (NP_o) of IP_3R1 was the same for 10 and 100 μM of **2**, confirming that both concentrations were maximally effective (Figures 2D and 2E). However, the maximal NP_o for **2** was 40-fold less than for IP_3; the single-channel conductance (γ_K) was the same for both agonists (Figures 2D and 2E; Table S1). Because partial agonists bind to the same site as full agonists, but less effectively activate the receptor, they behave as competitive antagonists of full agonists. This behavior is evident in patch-clamp and Ca^{2+}-release assays, where **2** reduced the response to IP_3 (Figures 2D–2F). These observations, which are expected for a partial agonist, are important for subsequent experiments because they demonstrate that IP_3 and **2** compete for occupancy of the same IP_3Rs, but **2** causes less effective activation.

The K_D of **2** determined from dose-ratio analysis (K_D = 426 nM) (Table S3) was similar to the half-maximally effective concentration of **2** in Ca^{2+}-release assays (EC_{50} = 479 nM, Table S1), as expected for a weak partial agonist. The K_D for **2** derived from functional assays (~450 nM) is much greater than that determined by radioligand binding (5.2 nM) because they use different assay conditions (Ding et al., 2010). However, in binding analyses performed under conditions that mimic functional assays, the K_D for **2** was 549 nM, which is comparable to its EC_{50} for Ca^{2+} release, and 1.5-fold lower than the K_D for IP_3 determined under these conditions (794 nM) (Figure 2G).

We reported previously that the reduced open probability (P_o) of IP_3Rs activated by partial agonists was due to an increase in mean channel closed time (τ_c), with no effect on mean open time (τ_o) (Rossi et al., 2009). Similar behavior appears to underlie the reduced efficacy of **2** because the modest (~2-fold) decrease in τ_o (relative to IP_3) is insufficient to explain the 40-fold decrease in NP_o (Table S1). With such low P_o, it is impracticable to determine τ_c directly for **2**.

IP_3 binds to the IP_3-binding core of the IP_3R (IBC, residues 224–604) (Bosanac et al., 2002), but communication with the channel requires the suppressor domain (SD, residues 1–223) (Rossi et al., 2009; Yoshikawa et al., 1999) (Figure 2H). The difference in ligand affinities ($\Delta\Delta G = \Delta G^{IBC} - \Delta G^{NT}$, where $\Delta G = -RT ln K_D$) for the IBC and N-terminal (NT, residues 1–604) reports the binding energy diverted into changing the NT conformation (Rossi et al., 2009). Analyses of IP_3 and **2** binding to the IBC and NT confirm that IP_3 diverts more energy into changing the NT conformation than does **2** (Figures 2I and 2J; Table S1).

We conclude that **2** is a high-affinity, partial agonist of IP_3R. It is the weakest known partial agonist with high affinity for IP_3R. The basis of its low efficacy is similar to that of related partial agonists (Rossi et al., 2009): it perturbs communication between the IBC and SD, causing the channel to dwell longer in a closed state and so open infrequently (Figure 2K). Our results suggest strategies to develop high-affinity antagonists of IP_3R and they provide the tool needed to assess whether all-or-nothing emptying of Ca^{2+} stores with heterogenous IP_3R sensitivities underlies quantal responses (Figure 1A).

Quantal responses are not mediated by heterogenous stores

Compound **2** stimulated quantal Ca^{2+} release, and the responses to sequential additions were incremental (Figures 3A–3D). However, the quantal response evoked by a maximally effective concentration of **2** (Figures 3A and 3D) was smaller than the maximal response to IP_3 (Figure 2C). Hence, even when all IP_3Rs are occupied by **2**, the IP_3-sensitive Ca^{2+} stores are not fully depleted. However, the Ca^{2+} stores remain sensitive to a subsequent IP_3 addition, albeit at a high concentration of IP_3 because it must now surmount the competitive antagonism of **2** (Figures 3E and 3F). Since **2** evokes quantal Ca^{2+} release when it occupies all IP_3Rs, the quantal phenomenon cannot be due to all-or-nothing emptying of stores with heterogenous sensitivities. The response to a maximal concentration of **2** in Figure 3A is biphasic, and we also observed some biphasic responses to IP_3 (see Figures S5L and S5M), but such biphasic responses were not consistently observed and were not further analyzed.

When cells are maximally stimulated with **2**, IP_3 can bind to IP_3R only after **2** dissociates. Rapid IP_3-evoked Ca^{2+} release after maximal stimulation with **2** (Figures 3E and 3F) therefore demonstrates that quantal Ca^{2+} release is accompanied by rapid dissociation and re-association of agonists: IP_3 is not "locked" onto activated IP_3Rs. We conclude that during quantal responses, there is incomplete emptying of the entire ER, and IP_3 continues to associate with and dissociate from IP_3Rs (Figures 1B and 3G).

Quantal Ca^{2+} release is not a property of the ER

Since quantal Ca^{2+} release occurs with IP_3Rs (Figure 1) (Yamashita, 2006) and RyRs (Cheek et al., 1994; Wang et al., 2004), it might reflect properties of the ER rather than its channels. Increases in $[Ca^{2+}]_c$ can, for example, restructure the ER (Subramanian and Meyer, 1997), suggesting that loss of Ca^{2+} from the ER might cause its further fragmentation and deny active Ca^{2+} channels access to the remaining Ca^{2+}. Transient receptor potential mucolipin 1 (TRPML1) channels are usually expressed in the membranes of lysosomes (Shen et al., 2012), but when overexpressed TRPML1 channels also release Ca^{2+} from the ER and some TRPML1 populates the reticular ER in which IP_3Rs are expressed (Figures 4A and S5A–S5F). In permeabilized HEK cells expressing CFP-TRPML1, but not in mock-transfected cells, ML-SA1 (a selective TRPML1 agonist) (Shen et al., 2012) stimulated a concentration-dependent Ca^{2+} release from the ER (Figures 4A and S5A). In contrast to the quantal responses evoked by IP_3 and **2**, ML-SA1 caused a concentration-dependent

(F) Summary results (individual values, mean ± SEM, n = 5) show Ca^{2+} release (determined at 500 s) evoked by the indicated concentrations of **2** followed by IP_3 in wild-type HEK cells. $^{**}p < 0.01$, $^{***}p < 0.001$, $^{****}p < 0.0001$; ns, not significant (for indicated comparisons, ANOVA with Bonferroni's test).

(G) Maximal IP_3 concentrations fully empty the IP_3-sensitive Ca^{2+} stores, but it was unclear whether quantal responses to submaximal IP_3 concentrations arise from incomplete emptying of the entire ER (*i*) or complete emptying of a fraction of the ER (*ii*). Results with a maximally effective concentration of **2**, which partially activates all IP_3Rs leaving Ca^{2+} trapped within the ER, indicate that quantal responses are not due to all-or-nothing emptying of heterogenous stores (*ii*).

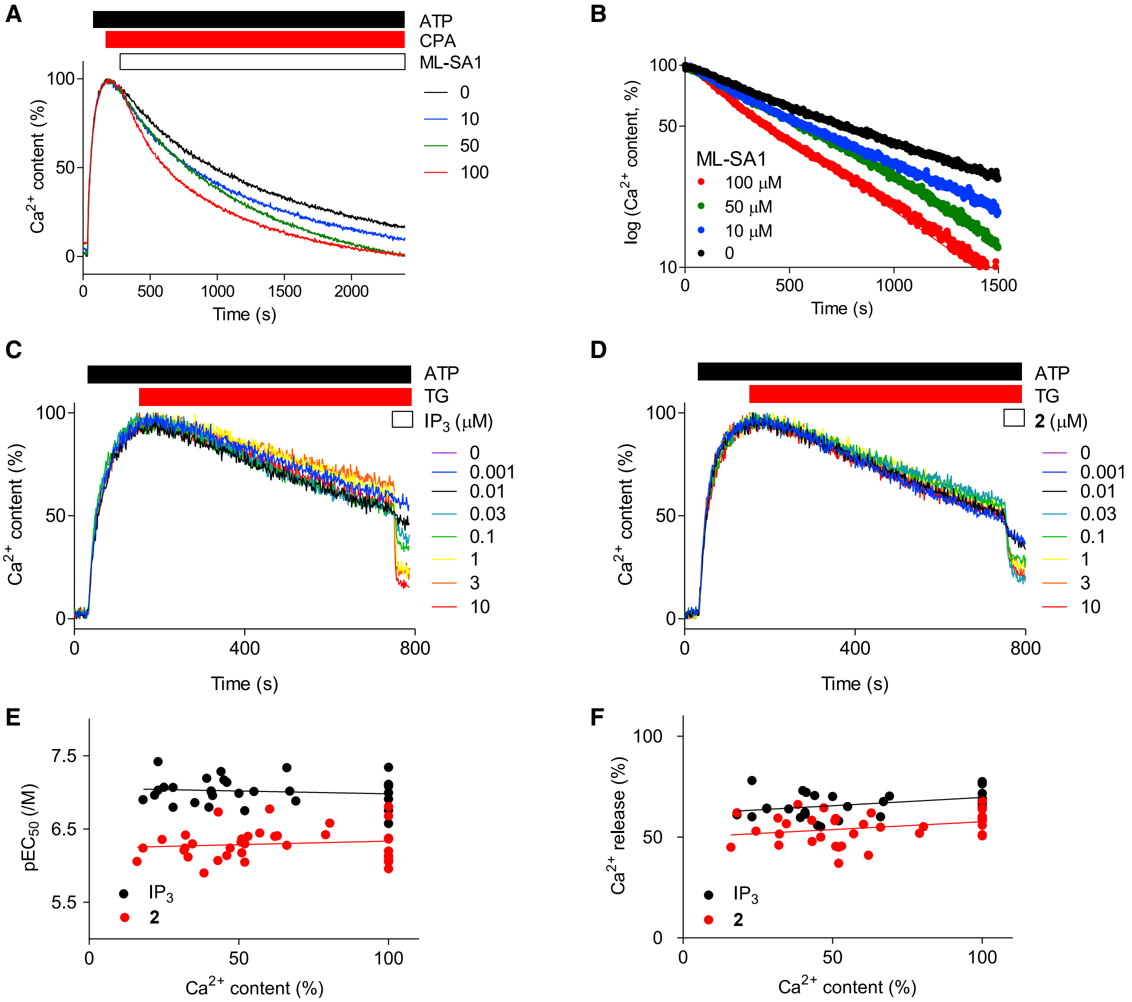

Figure 4. Neither ER reorganization nor luminal Ca^{2+} mediates quantal Ca^{2+} release

(A) Effects of the indicated concentrations of ML-SA1 on the ER Ca^{2+} content of permeabilized HEK cells expressing CFP-TRPML1.

(B) Semi-logarithmic plots show mono-exponential loss of ER Ca^{2+} after treatment with ML-SA1. Results (A and B) are typical of four experiments, each with duplicate determinations. Summary results are in Figure S5B.

(C and D) Permeabilized DT40-IP_3R1 cells were loaded to steady state with Ca^{2+} before addition of thapsigargin (TG, 1 μM) to inhibit SERCAs. At intervals thereafter (0–35 min), IP_3 (C) or **2** (D) was added. Results are typical of 29–36 experiments.

(E and F) Summary results (each point from a single concentration-effect relationship) show the relationships between ER Ca^{2+} content at the time of addition of IP_3 or **2** (%) and pEC_{50} value ($-logEC_{50}$) (E) and maximal Ca^{2+} release (F). Lines (least-squares linear regression) have slopes that are not significantly different from 0.

See also Figure S5.

increase in the rate of Ca^{2+} release from the entire ML-SA1-sensitive ER (Figures 4B and S5B). These results establish that quantal Ca^{2+} release is not a property of the ER or an inexorable consequence of cell permeabilization with the associated ER fragmentation (Figure S4O), but it is instead a feature of IP_3Rs and RyRs. That conclusion is consistent with evidence that purified IP_3Rs mediate quantal Ca^{2+} release (Ferris et al., 1992).

Neither luminal Ca^{2+} nor erlin 2 determines quantal responses

An appealing hypothesis is that luminal Ca^{2+} is required for IP_3R gating, such that as the ER loses Ca^{2+} the IP_3R channel closes trapping Ca^{2+} within the ER (Irvine, 1990) (Figure 1C). Previous analyses of the effects of luminal Ca^{2+} provide conflicting results. There is evidence that a very substantial loss of ER Ca^{2+} reduces IP_3R sensitivity (Barrero et al., 1997; Marshall and Taylor, 1994) and that overloading stores with Ca^{2+} promotes Ca^{2+} release (Missiaen et al., 1992), while other reports suggest that luminal Ca^{2+} reduces IP_3R sensitivity (Vais et al., 2020) or has no effect (Beecroft and Taylor, 1997; Shuttleworth, 1992).

We anticipated that if three ligands are required to open IP_3Rs (IP_3, cytosolic Ca^{2+}, and luminal Ca^{2+}, Figure 1C), channel opening by IP_3 might require less luminal Ca^{2+} than the weak partial agonist **2**. We used thapsigargin to irreversibly inhibit SERCA

CellPress
OPEN ACCESS

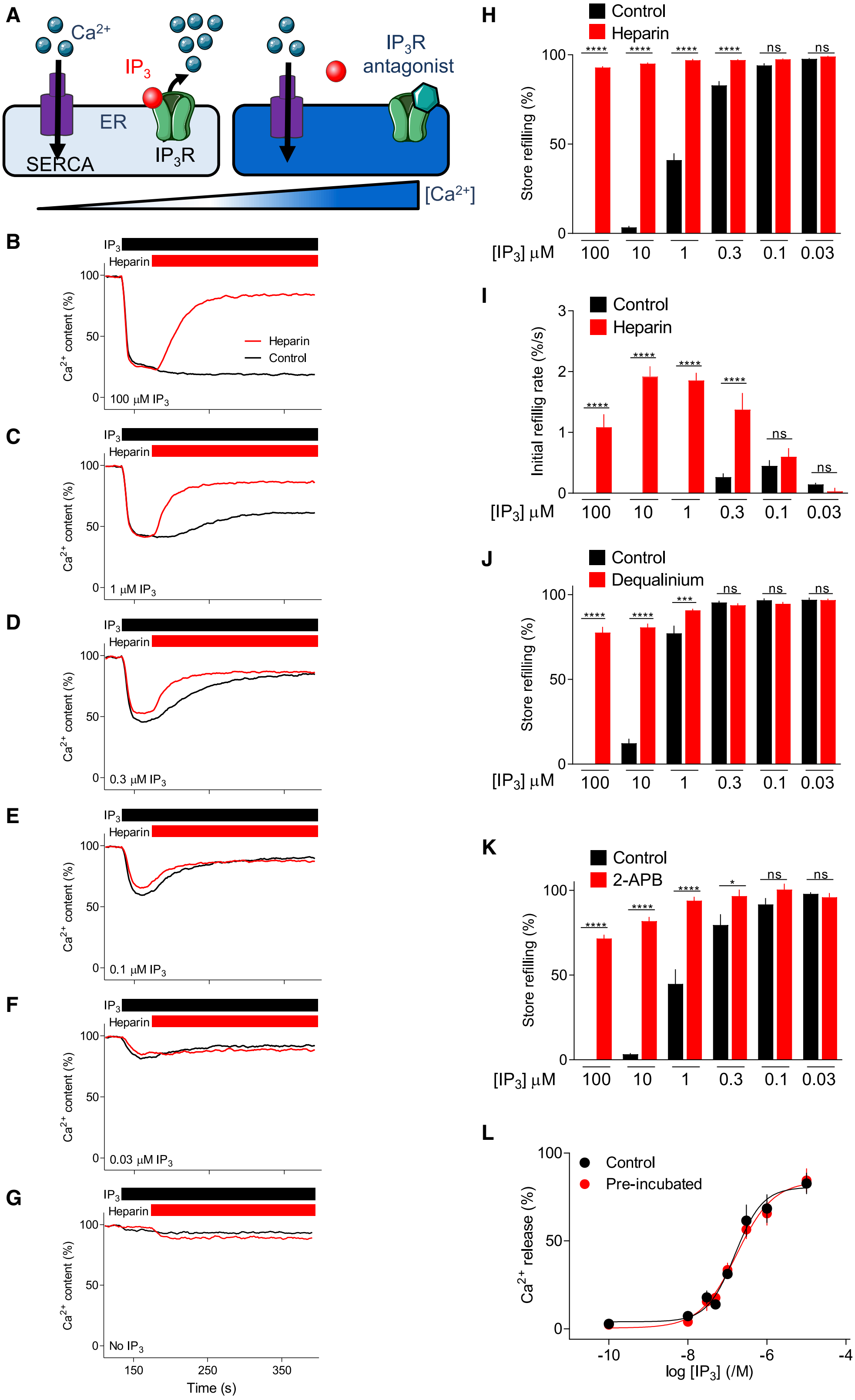

(legend on next page)

and slowly drain the ER of Ca^{2+}, and then evaluated responses to IP_3 and **2** at different ER Ca^{2+} contents (Figures 4C and 4D). The fraction of the remaining Ca^{2+} stores released by IP_3 or **2** and the EC_{50} for each ligand were unaffected as stores lost their Ca^{2+}. We conclude that there is no significant effect of ER Ca^{2+} content on the sensitivity or maximal response to IP_3 or **2** (Figures 4E and 4F), even when the Ca^{2+} content is reduced to levels well below those observed after quantal responses to IP_3 or **2**. Luminal Ca^{2+} does not, therefore, affect the number of open IP_3Rs. We conclude, and consistent with some previous reports (Beecroft and Taylor, 1997; Combettes et al., 1992; Hajnóczky and Thomas, 1994; Oldershaw et al., 1991; Shuttleworth, 1992), that regulation of IP_3Rs by luminal Ca^{2+} does not underlie quantal responses.

Activation of IP_3Rs initiates a sequence that can lead to their proteasomal degradation. An early step in this sequence is recognition of active IP_3Rs by an ER-membrane protein, erlin 2 (ER lipid raft-associated protein 2) (Wojcikiewicz, 2018). We considered whether erlin 2 might associate with active IP_3Rs, terminate their activity, and thereby contribute to quantal Ca^{2+} release. However, substantial depletion of erlin 2 using small interfering RNA (siRNA) had no effect on quantal responses to IP_3 (Figures S5G–S5M). We conclude that neither erlin 2 nor changes in ER luminal [Ca^{2+}] contribute to incremental Ca^{2+} release by IP_3.

IP_3 evokes Ca^{2+} release and then IP_3R inactivation

Previous analyses of IP_3R inactivation provide conflicting results, perhaps indicating a need for unidentified labile accessory factors (Bezprozvanny and Ehrlich, 1994; Hajnóczky and Thomas, 1994; Mak and Foskett, 1997; Marchant and Taylor, 1998; Oldershaw et al., 1992; Taufiq-Ur-Rahman et al., 2009; Stehno-Bittel et al., 1995). To examine any possible contribution of IP_3R inactivation to quantal Ca^{2+} release, we therefore examined IP_3R inactivation under conditions that exactly replicate those we used to show incremental responses to IP_3.

To assess the activity of IP_3Rs during the sustained phase of incremental responses, we examined IP_3-evoked Ca^{2+} release without inhibiting SERCAs and then added an IP_3R antagonist during the response. The rationale is that if IP_3Rs remain active, an antagonist should reverse any ongoing activity and allow stores to refill (Figure 5A). We used three unrelated antagonists since each has limitations (Figure S6). Heparin is a competitive antagonist (Richardson and Taylor, 1993) and 2-aminoethoxydiphenyl borate (2-APB) is a non-competitive inhibitor with some selectivity for IP_3R1 (Saleem et al., 2014). We show that dequalinium, which blocks K^+ channels, is also an IP_3R antagonist (Figures S6A–S6G). At the concentrations used, the antagonists completely (heparin and dequalinium) or partially (2-APB) blocked IP_3-evoked Ca^{2+} release from permeabilized HEK-IP_3R1 cells (Figures S6H–S6J).

During sustained stimulation of permeabilized HEK-IP_3R1 cells with a maximal concentration of IP_3, the stores remained empty, but they refilled after adding heparin (Figure 5B). The incomplete refilling is probably due to partial inhibition of SERCAs by heparin (Figures S6K–S6M). The results demonstrate that during maximal stimulation, enough IP_3Rs remain open to counteract opposing SERCA activity. Since an IP_3R can conduct $\sim$500,000 Ca^{2+}/s (Vais et al., 2010) while a SERCA transports fewer than 40 Ca^{2+}/s (Lytton et al., 1992), it may require very little residual IP_3R activity to overwhelm SERCAs and keep the ER depleted of Ca^{2+}. After stimulation with the lowest IP_3 concentrations (30–100 nM), the stores rapidly refilled and the rate was unaffected by heparin (Figures 5E–5I). This indicates that after the initial Ca^{2+} release evoked by low IP_3 concentrations, there were no detectable open IP_3Rs. The pattern of response to intermediate concentrations of IP_3 fell between these extremes: there was residual sustained IP_3R activity with the higher IP_3 concentrations, and IP_3R inactivation occurred with the lower concentrations, but more slowly than for the lowest IP_3 concentration (Figures 5C, 5D, 5H, and 5I). Similar results were obtained with different antagonists (Figures 5J, 5K, and S7), with heparin in wild-type HEK cells (Figures S7M–S7T), and with a metabolically stable analog of IP_3, (2,4,5)IP_3 (Figures 6A-6H). We also used a bioassay of IP_3 to confirm that refilling of Ca^{2+} stores during prolonged incubation with low concentrations of IP_3 was not due to IP_3 degradation (Figure 5L; Table S4).

Previous studies provide conflicting evidence for IP_3-evoked IP_3R inactivation. Single-channel recordings of IP_3Rs support (Mak and Foskett, 1997; Stehno-Bittel et al., 1995) or challenge inactivation (Bezprozvanny and Ehrlich, 1994; Taufiq-Ur-Rahman et al., 2009), and while some analyses of permeabilized cells show IP_3-evoked inactivation (Hajnóczky and Thomas, 1994; Marchant and Taylor, 1998), others suggest that responses are

Figure 5. IP_3Rs close after rapid Ca^{2+} release evoked by low IP_3 concentrations

(A) If IP_3Rs remain active after an incremental Ca^{2+} release; stores should re-accumulate Ca^{2+} after addition of an IP_3R antagonist.

(B–G) The ER of permeabilized HEK-IP_3R1 cells was loaded with Ca^{2+} by addition of ATP (t = 0); IP_3 (0–30 μM) and heparin (10 mg/mL) were then added as indicated. Each trace (mean of duplicate determinations) shows results with a different IP_3 concentration. The code in (B) applies to all panels.

(H and I) Summary results (mean ± SEM, n = 6, each with two determinations) show the extent to which stores have refilled (relative to cells without IP_3) 250 s after IP_3 addition (H), as well as the initial rate of refilling from the slope of the curve immediately after heparin addition (I). In (H), 0% refilling is defined as the store content in control conditions measured 250 s after addition of a maximal IP_3 concentration. In these experiments, the EC_{50} for IP_3-evoked Ca^{2+} release was 166 nM (pEC_{50} = 6.78 ± 0.08) and maximal Ca^{2+} release was 86% ± 2% (mean ± SEM, n = 6).

(J and K) Summary results (mean ± SEM, n = 5–7, each with duplicate determinations) from similar analyses using 50 μM dequalinium (J; Figures S7A–S7F) or 125 μM 2-APB (K; Figures S7G–S7L) to block IP_3Rs. *$p < 0.05$, ***$p < 0.001$, ****$p < 0.0001$, one-way repeated ANOVA with Bonferroni's test for the indicated comparisons (H–K).

(L) IP_3 was incubated with permeabilized HEK-IP_3R1 cells (pre-incubated) or without cells (control) for 260 s under conditions identical to those used to show IP_3R inactivation. The supernatant was then bio-assayed. Summary results (mean ± SEM, n = 4, each with three replicates) show no significant difference between the potency of IP_3 after incubation under control conditions or with permeabilized cells. The results establish that refilling of stores during sustained incubation with low concentrations of IP_3 is not due to its metabolism. Table S4 provides a summary and detailed methods.

See also Figures S6 and S7 and Table S4.

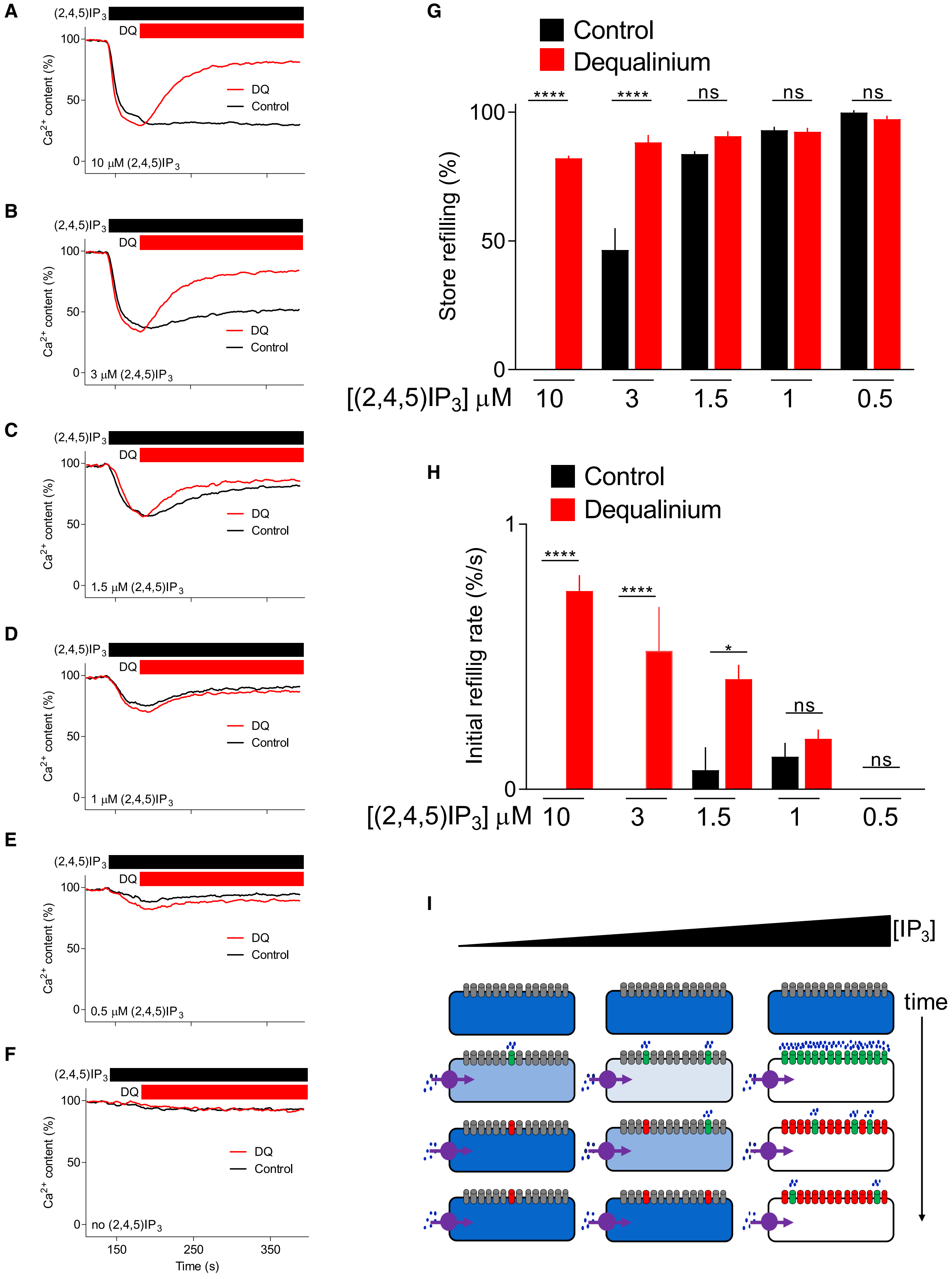

(legend on next page)

sustained (Oldershaw et al., 1992). Our results, from analyses where incremental responses and inactivation were assessed under identical conditions, show that incremental responses to low IP_3 concentrations are accompanied by rapid inactivation of IP_3Rs (Figure 6I).

Incremental responses involve activation of very few IP_3Rs

If quantal responses to low IP_3 concentrations are terminated by closure of IP_3Rs, how can stores retain undiminished responsiveness to further IP_3 additions? Comparison of the relationship between the occupancy of IP_3Rs by IP_3 with functional responses provides a possible answer to this conundrum.

Analyses of $[^3H]IP_3$ binding establish the fraction of IP_3-binding sites to which each concentration of IP_3 has bound (α) without revealing how the binding is distributed between IP_3Rs or their different states. However, Hill coefficients for IP_3 binding to IP_3R1 are close to one (0.95 ± 0.08 in Figure 2B) (Hannaert-Merah et al., 1994; Iwai et al., 2005; Rossi et al., 2009; Supattapone et al., 1988), indicating that each IP_3R subunit binds IP_3 independently (Hannaert-Merah et al., 1994) and that differences in the affinities of different IP_3R states are either small or masked by the predominance of a single state. We therefore assume that IP_3 binding is independently distributed across all available IP_3-binding sites. Since IP_3R opening requires binding of IP_3 to all four IP_3R subunits (Alzayady et al., 2016), we need to predict the relationship between IP_3 concentration and the fraction of IP_3R with all four sites occupied (α^4, Figure 7A). We begin with analyses of HEK-IP_3R1 cells.

Our measurements of IP_3-evoked Ca^{2+} release lack the temporal resolution needed to determine the initial rates of Ca^{2+} release that would directly report the number of open IP_3Rs (Marchant and Taylor, 1998). However, the assays do establish the IP_3 sensitivity of quantal responses, because (with SERCAs inhibited) the Ca^{2+} release determined after 20 s for each IP_3 concentration reports the cumulative response to IP_3 throughout that interval. When addressing mechanisms of quantal Ca^{2+} release, it is, therefore, appropriate to compare the sensitivity to IP_3 of quantal Ca^{2+} release (the fraction of Ca^{2+} stores released) to estimates of how many IP_3Rs are likely to be tetra-liganded by that concentration of IP_3. We used IP_3R1 from cerebellum (2.75×10^8 $IP_3Rs/\mu L$ of sample, determined from $[^3H]IP_3$ binding) to calibrate western blots, and so determine the number of IP_3Rs in HEK-IP_3R1 cells (49,146 ± 15,001 IP_3Rs/cell) (Figure 7B). Comparing the affinity of IP_3R1 for IP_3 under conditions that replicate functional assays (Figure 2G) with the number of IP_3Rs in HEK-IP_3R1 cells, we predict that the Ca^{2+} release evoked by a half-maximally effective concentration of IP_3 is associated with opening of only 0.13% of a cell's IP_3Rs, or about 60 IP_3Rs/cell (Figures 7C and 7D). This situation, where only a tiny fraction of available receptors elicits a response, is analogous to the "spare receptors" found in many signaling pathways initiated by plasma membrane receptors. Here, all receptors are competent to respond, but there are many more receptors than required to elicit a maximal response (Stephenson, 1956). Our argument does not require an additional level of IP_3R regulation, although that may also occur (Thillaiappan et al., 2017; Thillaiappan et al., 2021); it requires only that cells express more IP_3Rs than are needed to deplete the ER of Ca^{2+}. In analogy with spare receptors elsewhere, we expect this feature to increase the sensitivity of Ca^{2+} release to IP_3.

An open IP_3R is predicted to mediate movement of about 500,000 Ca^{2+}/s out of the ER (Vais et al., 2010). We estimate the ER Ca^{2+} content of a single permeabilized HEK cell to be about 9×10^{-16} mol (see *Simulations* in STAR Methods). This suggests that sustained opening of a single IP_3R for the 20 s taken for incremental responses to plateau would be sufficient to cause loss of almost 2% of the ER Ca^{2+} content of a cell. The key point is that very few of the many IP_3Rs in a cell are active during incremental responses to IP_3 and they are sufficient to account for the observed responses (Figure 7D).

A mechanism for incremental responses to IP_3

We propose that after IP_3 addition, very few of the many IP_3Rs in a cell rapidly bind IP_3 to each of their four subunits; many more IP_3Rs will be partially occupied (Figures 7A, 7C, and 7D). The fully occupied IP_3Rs open and rapidly release Ca^{2+}; they then inactivate and more slowly recover (Figure 7E). After the initial Ca^{2+} release, when activation of IP_3Rs is effectively synchronized by IP_3 addition, additional IP_3Rs will open as they bind four IP_3 molecules and then proceed to inactivate, but their openings will be asynchronous. Hence, addition of IP_3 drives a few IP_3Rs through a near-synchronous sequence of opening and inactivation, after which very small numbers of IP_3Rs proceed through the sequence asynchronously. The next step in IP_3 concentration then drives another pulse of near-synchronous IP_3R openings.

Two features of this scheme contribute to understanding incremental responses. First, incremental responses are mediated by only a tiny fraction of the IP_3Rs in a cell (<1%, Figures 7C and 7D). Inactivation therefore negligibly impacts the number of IP_3Rs available for subsequent activation, ensuring that despite some inactivation of IP_3Rs, responses to subsequent IP_3 additions are unperturbed. Second, because IP_3Rs move inexorably from an open to an inactivated state, there will always be more open IP_3Rs immediately after an IP_3 addition (when openings

Figure 6. IP_3Rs of HEK-IP_3R1 cells close after incremental responses to low concentrations of a stable analog of IP_3

(A–F) The experiments are similar to those shown in Figure 5, but using HEK-IP_3R1 cells, (2,4,5)IP_3 at the indicated concentrations, and dequalinium (DQ, 50 μM). Permeabilized cells were loaded with Ca^{2+} by addition of ATP before adding the indicated concentrations of (2,4,5)IP_3 (without inhibiting SERCAs) and then the IP_3R antagonist, dequalinium. Traces show means of two replicates.

(G and H) Summary results (mean ± SEM, n = 4, each with two determinations) show the extent to which stores refill (relative to cells without (2,4,5)IP_3) 250 s after (2,4,5)IP_3 addition (G), and the initial rate of refilling from the slope of the curve immediately after DQ addition (H). *$p < 0.05$, ****$p < 0.0001$, one-way ANOVA with Bonferroni's test.

(I) Stores rapidly refill after stimulation with the lowest IP_3 concentrations, as the very few active IP_3Rs inactivate. Higher concentrations of IP_3 activate many more IP_3Rs than needed to empty the stores, hence even substantial inactivation leaves enough active IP_3Rs to keep the stores empty.

See also Figures S6 and S7.

CellPress
OPEN ACCESS

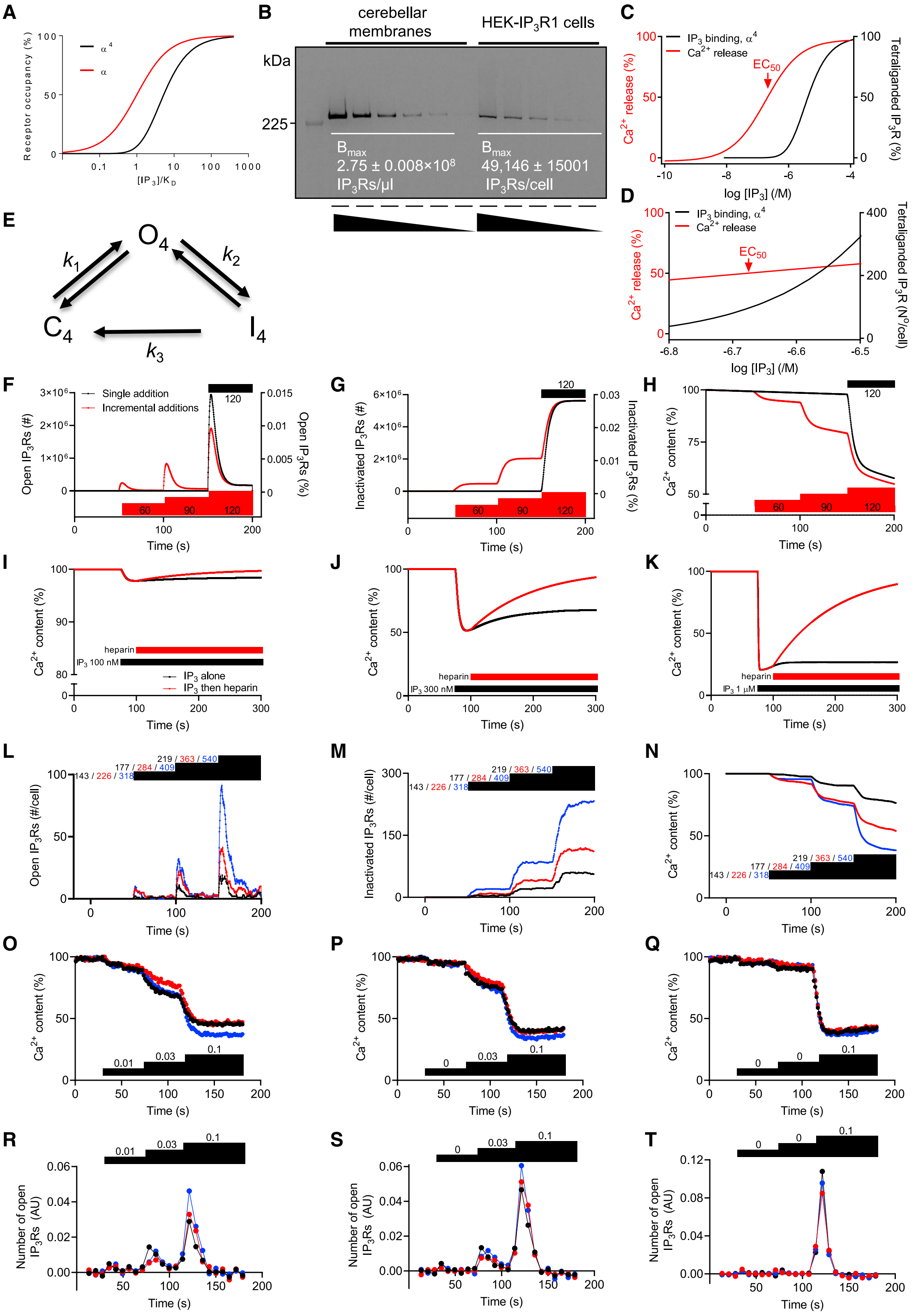

(legend on next page)

are synchronized) than later (when IP_3Rs progress to inactivated states) (Figure 7E). A surge of Ca^{2+} release after each IP_3 addition is followed by a period of much reduced activity, thereby explaining the quantal pattern of Ca^{2+} release.

There is evidence, albeit with conflicting observations, that rapid activation of IP_3Rs by IP_3 is followed by slower inactivation (half-time [$t_{1/2}$] of about 15–30 s) (Hajnóczky and Thomas, 1994; Mak et al., 2000, 2001; Stehno-Bittel et al., 1995) and even slower recovery ($t_{1/2}$ of 30 s to several minutes) (Hajnóczky and Thomas, 1994; Ionescu et al., 2006). This mechanism would allow incremental responses only if inactivation requires most (probably all four) IP_3-binding sites to be occupied; otherwise, IP_3Rs would inactivate before they opened. An alternative mechanism is "feed-through" inhibition of IP_3Rs by Ca^{2+} as it passes through an open channel (Dawson et al., 2003), but even these very local Ca^{2+} signals would probably be intercepted by 10 mM BAPTA (Vais et al., 2012), which did not prevent quantal responses (Figures S2A–S2F). We suggest that IP_3R inactivation is directly linked to activation rather than via feedback effects from increased $[Ca^{2+}]_c$.

A simple activation-inactivation scheme predicts incremental responses to IP_3

We developed an empirical scheme with stochastic simulations to assess whether it captured key features of the experimental observations. A simplified scheme is needed to reduce computation times for the stochastic simulations of the many IP_3Rs required to address the consequences of a few IP_3Rs opening together and then losing synchrony as they inactivate. The scheme envisages that an IP_3R opens rapidly ($t_{1/2}$ of ~2.5 s) after it has bound four molecules of IP_3, before slowly inactivating ($t_{1/2}$ of ~5 s) and then very slowly recovering from the inactivated to the closed state ($t_{1/2}$ of ~250 s) (Figure 7E). The detailed mechanisms of IP_3-evoked IP_3R inactivation are unresolved and may require interaction with additional unidentified molecules. Hence, our empirical scheme based on the activation-inactivation sequence shown in Figure 7E is not a closed cycle (see *Simulations* in STAR Methods).

Mathematical simulations using our simplified scheme (Figure 7E) and with the numbers of IP_3Rs determined in functional analyses of HEK-IP_3R1 cells in 96-well plates (4×10^5 cells/well, ~2×10^{10} IP_3Rs/well) (Figure 7B) capture the key experimental observations. Each addition of a submaximal IP_3 concentration evokes a surge of IP_3R openings followed by stable inactivation of a small fraction of the IP_3R population. The ER Ca^{2+} content responds incrementally to successive IP_3 additions. Finally, with SERCAs active, stores rapidly refill in the presence of low IP_3 concentrations, but remain depleted with higher concentrations (Figures 7F–7K).

Most of our analyses used populations of cells in 96-well plates, but incremental responses to IP_3 are also evident in single wild-type HEK cells (Figures S4J–S4N). We therefore measured the number of IP_3Rs in wild-type HEK cells (9,101 ± 934 IP_3Rs/cell, mean ± SD, n = 3) and applied our scheme to a single cell. The simulations confirm that here too, the scheme predicts behavior consistent with experimental observations. Incremental additions

Figure 7. Incremental responses are mediated by very few IP_3Rs that rapidly inactivate

(A) IP_3 must bind to all four IP_3-binding sites of a tetrameric IP_3R for the channel to open (Alzayady et al., 2016). Since IP_3 binds independently to each site and with the same K_D, we compute the fractional occupancy of all IP_3-binding sites (α) at any ligand concentration ([L]) from: $\alpha = ([L]/K_D/1+[L]/K_D)$. The fraction of tetrameric IP_3R in which all four sites are occupied is then α^4. The plot shows the relationship between normalized ligand concentration ($[L]/K_D$) and the fraction of IP_3Rs in which all four IP_3-bindings sites are occupied (α^4) compared to the fractional occupancy at a single site (α).

(B) Equilibrium competition binding with $[^3H]IP_3$ in TEM was used to define the K_D and from that the maximal number of binding sites (B_{max}) in cerebellar membranes, which were then used to calibrate western blots to estimate the number of IP_3Rs in a HEK-IP_3R1 cell. Loadings are for serial 2-fold dilutions from 3.2 to 0.1 μL/lane (cerebellum) and from 12.8 to 0.8 μL/lane (HEK-IP_3R1 cells). The HEK-IP_3R1 cell lysate contained 10,000 cells/μL. B_{max} values are mean ± SEM, n = 6.

(C) Predicted relationship for HEK-IP_3R1 cells between IP_3 concentration, number of IP_3Rs (% of total) with all four IP_3-binding sites occupied (calculated from the K_D for IP_3 determined under conditions used for functional assays, 794 nM), and IP_3-evoked Ca^{2+} release (% of IP_3-sensitive stores; EC_{50} = 186 nM, pEC_{50} = 6.73 ± 0.04; n = 17).

(D) Relationships between IP_3 concentrations around the EC_{50} value, Ca^{2+} release, and the number of tetra-liganded IP_3Rs/cell.

(E) We assume that IP_3Rs in which four subunits have bound IP_3 move between closed (C_4), open (O_4), and inactivated (I_4) states with forward rate constants $k_1 > k_2 >> k_3$.

(F–H) Simulations show numbers of open (F) and inactivated IP_3Rs/well (G), and the ER Ca^{2+} content (H) for HEK-IP_3R1 cells after sequential additions to give the indicated final IP_3 concentrations (nM) or a single addition of the final IP_3 concentration. Each addition of IP_3 evokes a rapid, transient surge in the number of open IP_3Rs, which then rapidly inactivate, leading to transient Ca^{2+} release. Note the tiny fraction of open and inactivated IP_3Rs (right axes in F and G).

(I–K) Simulations for HEK-IP_3R1 cells with SERCAs active show ER Ca^{2+} content after addition of the indicated IP_3 concentrations and then heparin (similar to analyses in Figures 5 and 6). Addition of heparin was simulated by making k_{rel} = 0.

(L–N) In the simulations of cell populations (F–H), stepwise increases in IP_3 concentration caused the predicted number of tetra-liganded IP_3Rs (IP_3R^4) to increase by 4.41-fold (for 60 to 90 nM) and then by 2.76-fold (90 to 120 nM). Here, we simulate responses from a single wild-type HEK cell expressing 9,101 IP_3Rs, with the first IP_3 addition triggering formation of 5 IP_3R^4s/cell (143 nM), 10 IP_3R^4s/cell (177 nM), or 20 IP_3R^4s/cell (219 nM). We then deliver two further IP_3 additions calculated to cause the numbers of IP_3R^4s to increase by 4.41-fold and then by 2.76-fold to mimic the stimuli used in simulations of larger numbers of cells (F–G). Simulations show the effects of adding the indicated concentrations of IP_3 on the number of open (L) and inactivated IP_3Rs (M), and the Ca^{2+} content of the ER (N). Colored code indicates concentrations of IP_3 (nM) added at each step.

(O–T) We predict the number of open IP_3Rs during incremental responses of permeabilized DT40-IP_3R1 cells by subtracting the ER Ca^{2+} leak after inhibition of SERCA (Figure 1D, purple trace) from the results obtained with incremental additions of IP_3 (Figures 1G–1H), and then estimate the time-dependent loss of ER Ca^{2+} due to opening of IP_3Rs (see *Analysis* in STAR Methods). For each sequence of IP_3 additions, results are shown for each of three independent analyses (blue, red, and black). The results show that after each IP_3 addition, IP_3Rs abruptly open and then close (inactivate), and the time course of the number of open IP_3Rs matches predictions of the simple activation-inactivation scheme (F–H), confirming that our scheme captures essential features of IP_3Rs responding incrementally to IP_3.

of IP_3 evoke graded loss of ER Ca^{2+}, which is associated with transient opening of very small numbers of IP_3Rs and sustained inactivation of a few IP_3Rs (Figures 7L–7N).

We also compared the activation-inactivation scheme with experimental data from DT40-IP_3R1 cells responding incrementally to IP_3 (Figures 1G–1J). Using the experimental data, we identified the component of ER Ca^{2+} release attributable to open IP_3Rs by subtracting the basal IP_3-independent Ca^{2+} leak (see *Analysis* in STAR Methods). By dividing this IP_3R-dependent Ca^{2+} flux by the time-matched ER Ca^{2+} content, we estimated the time course of the number of open IP_3Rs (in arbitrary numbers) during sequential additions of IP_3 (Figures 7O–7T). The analysis establishes that IP_3Rs must close within about 10 s of each IP_3 addition. Furthermore, the close correspondence of the time course of IP_3R openings in the experimental analysis with the predictions of our simple, albeit incomplete, activation-inactivation scheme (Figure 7E) confirms that the scheme captures all essential features of the experimental observations.

Conclusions

Quantal Ca^{2+} release is an unusual feature of IP_3Rs, first described in 1989 (Muallem et al., 1989), but never adequately explained (Yamashita, 2006). We confirmed that in both intact and permeabilized cells and under conditions where SERCAs cannot counteract IP_3R activity, submaximal concentrations of IP_3 rapidly release only a fraction of the ER Ca^{2+} stores without compromising responses to further incremental IP_3 additions. These features are properties of all three IP_3R subtypes; they are evident in single cells; they do not arise from ineffective charge compensation during electrogenic Ca^{2+} efflux, IP_3 metabolism, increases in $[Ca^{2+}]_c$, or mitochondrial activity; and they are a property of IP_3Rs rather than the ER.

We developed a synthetic high-affinity agonist of IP_3Rs with very low efficacy, **2**, to distinguish between the mechanisms proposed to explain quantal Ca^{2+} release (Figures 1A and 1B); **2** has the lowest efficacy reported for any high-affinity agonist of IP_3R. We used **2** to show that quantal responses do not arise from all-or-nothing emptying of heterogenous Ca^{2+} stores. We do not exclude the possibility that IP_3Rs within a cell may differ in their IP_3 sensitivity, but it is clear that any such heterogeneity is not required for quantal responses (Figure 3). Instead, after the initial rapid Ca^{2+} release evoked by low concentrations of IP_3, IP_3Rs close with Ca^{2+} still trapped in the ER. Neither erlin 2, an increase in $[Ca^{2+}]_c$, nor a decrease in ER luminal $[Ca^{2+}]$ contributes to this IP_3R inactivation. Instead, inactivation is probably an inexorable consequence of IP_3R activation (Hajnóczky and Thomas, 1994), although the detailed mechanisms are unresolved.

Comparison of receptor occupancy and functional responses reveals that only a tiny fraction of a cell's IP_3Rs contribute to incremental responses. These analyses, supported by simulations (Figures 7E–7T), reconcile incremental responses with IP_3R inactivation. A surge of coordinated opening of IP_3Rs occurs with each step in IP_3 concentration. This is followed by IP_3R inactivation. Thereafter, there will never be the same balance in favor of the open state until the next step in IP_3 concentration triggers another coordinated sequence of IP_3R opening and then inactivation (Figures 7F and 7L). Pulsatile delivery of IP_3 to intact cells may be provided by changes in extracellular stimulus intensity or by cycles of IP_3 production and degradation driven by feedback regulation of phospholipase C in the sustained presence of an extracellular stimulus (Bartlett et al., 2015; Dupont et al., 2011; Meyer and Stryer, 1988; Nash et al., 2001).

Our results suggest a mechanism for incremental responses to IP_3, a phenomenon that has remained unresolved for more than 30 years. Unrestrained IP_3Rs might evoke explosive responses through CICR. The mechanism we propose allows cells to evoke rapid Ca^{2+} signals through IP_3Rs that are graded with stimulus intensity (Figure 1P).

Limitations of the study

Two essential features underpin our explanation for incremental responses to IP_3: rapid inactivation of IP_3Rs after their opening, and physiological responses mediated by a tiny fraction of available IP_3Rs. We do not yet understand the molecular mechanisms underlying IP_3R inactivation, and notably whether accessory proteins are required. Understanding these mechanisms would allow us to refine our simplified scheme, which presently includes an irreversible step that forbids transitions from the closed (C_4) to inactivated (I_4) state of IP_3Rs. Hence, our simplified scheme is not a closed cycle, suggesting that it is coupled to an additional mechanism, perhaps reflecting the need for additional proteins to mediate inactivation. High-resolution optical microscopy reveals that low concentrations of IP_3 evoke small, highly localized Ca^{2+} signals ("Ca^{2+} puffs") in intact cells neighbors (Smith and Parker, 2009; Thillaiappan et al., 2021). It is not yet clear how incremental responses to IP_3 relate to Ca^{2+} puffs.

STAR★METHODS

Detailed methods are provided in the online version of this paper and include the following:

- KEY RESOURCES TABLE
- RESOURCE AVAILABILITY
  - Lead contact
  - Materials availability
  - Data and code availability
- EXPERIMENTAL MODEL AND SUBJECT DETAILS
  - Cells
- METHOD DETAILS
  - Synthesis of 2
  - Cell culture and transfections
  - Ca^{2+} release by IP_3Rs
  - Fluorescence microscopy
  - Single-cell analyses of Ca^{2+} release
  - $[^3H]IP_3$ binding
  - Nuclear patch-clamp recording from IP_3Rs
  - Western blotting
- QUANTIFICATION AND STATISTICAL ANALYSES
  - Simulations
  - Analysis

SUPPLEMENTAL INFORMATION

Supplemental information can be found online at https://doi.org/10.1016/j.celrep.2021.109932.

ACKNOWLEDGMENTS

This work was supported by the Biotechnology and Biological Sciences Research Council (BB/P005330/1 to C.W.T.), the Medical Research Council (MR/T028378/1 to C.W.T. and A.M. Rossi), Fondation Philippe Wiener-Maurice Anspach (to G.D. and C.W.T.), Senior Investigator Awards from Wellcome to C.W.T (101844) and B.V.L.P (101010), and a Royal Society Fellowship to T.R. (UF110479). A.M. Rossi is a fellow of Queens' College, Cambridge. G.D. is a Research Director at the Belgian FRS-FNRS. We thank Abdelhamid Yousef (Cambridge) for contributing ideas relating to erlin 2.

AUTHOR CONTRIBUTIONS

A.M. Rossi performed all biological experiments, except the single-channel recordings. T.R. performed single-channel recordings. A.M. Riley and B.V.L.P. developed and synthesized compound 2. G.D. implemented the mathematical models. C.W.T. and A.M. Rossi designed the study, analyzed and interpreted results, and, with input from the other authors, wrote the manuscript. All authors reviewed the final manuscript.

DECLARATION OF INTERESTS

The authors declare no competing interests.

Received: February 4, 2021
Revised: July 16, 2021
Accepted: October 12, 2021
Published: November 2, 2021

REFERENCES

Alzayady, K.J., Sebé-Pedrós, A., Chandrasekhar, R., Wang, L., Ruiz-Trillo, I., and Yule, D.I. (2015). Tracing the evolutionary history of inositol, 1,4,5-trisphosphate receptor: Insights from analyses of *Capsaspora owczarzaki* Ca^{2+} release channel orthologs. Mol. Biol. Evol. *32*, 2236–2253.

Alzayady, K.J., Wang, L., Chandrasekhar, R., Wagner, L.E., 2nd, Van Petegem, F., and Yule, D.I. (2016). Defining the stoichiometry of inositol 1,4,5-trisphosphate binding required to initiate Ca^{2+} release. Sci. Signal. *9*, ra35.

Ames, B.N., and Dubin, D.T. (1960). The role of polyamines in the neutralization of bacteriophage deoxyribonucleic acid. J. Biol. Chem. *235*, 769–775.

Arunlakshana, O., and Schild, H.O. (1959). Some quantitative uses of drug antagonists. Br. J. Pharmacol. Chemother. *14*, 48–58.

Barrero, M.J., Montero, M., and Alvarez, J. (1997). Dynamics of $[Ca^{2+}]$ in the endoplasmic reticulum and cytoplasm of intact HeLa cells. A comparative study. J. Biol. Chem. *272*, 27694–27699.

Bartlett, P.J., Metzger, W., Gaspers, L.D., and Thomas, A.P. (2015). Differential regulation of multiple steps in inositol 1,4,5-trisphosphate signaling by protein kinase C shapes hormone-stimulated Ca^{2+} oscillations. J. Biol. Chem. *290*, 18519–18533.

Beecroft, M.D., and Taylor, C.W. (1997). Incremental Ca^{2+} mobilization by inositol trisphosphate receptors is unlikely to be mediated by their desensitization or regulation by luminal or cytosolic Ca^{2+}. Biochem. J. *326*, 215–220.

Berridge, M.J. (2016). The inositol trisphosphate/calcium signaling pathway in health and disease. Physiol. Rev. *96*, 1261–1296.

Bezprozvanny, I., and Ehrlich, B.E. (1994). Inositol (1,4,5)-trisphosphate ($InsP_3$)-gated Ca channels from cerebellum: Conduction properties for divalent cations and regulation by intraluminal calcium. J. Gen. Physiol. *104*, 821–856.

Blagbrough, I.S., and Geall, A.J. (1998). Practical synthesis of unsymmetrical polyamine amides. Tetrahedron Lett. *39*, 439–442.

Bootman, M. (1994). Intracellular calcium. Questions about quantal Ca^{2+} release. Curr. Biol. *4*, 169–172.

Bootman, M.D., Berridge, M.J., and Taylor, C.W. (1992). All-or-nothing Ca^{2+} mobilization from the intracellular stores of single histamine-stimulated HeLa cells. J. Physiol. *450*, 163–178.

Bosanac, I., Alattia, J.-R., Mal, T.K., Chan, J., Talarico, S., Tong, F.K., Tong, K.I., Yoshikawa, F., Furuichi, T., Iwai, M., et al. (2002). Structure of the inositol 1,4,5-trisphosphate receptor binding core in complex with its ligand. Nature *420*, 696–700.

Callamaras, N., and Parker, I. (2000). Phasic characteristic of elementary Ca^{2+} release sites underlies quantal responses to IP_3. EMBO J. *19*, 3608–3617.

Cheek, T.R., Berridge, M.J., Moreton, R.B., Stauderman, K.A., Murawsky, M.M., and Bootman, M.D. (1994). Quantal Ca^{2+} mobilization by ryanodine receptors is due to all-or-none release from functionally discrete intracellular stores. Biochem. J. *301*, 879–883.

Combettes, L., Claret, M., and Champeil, P. (1992). Do submaximal $InsP_3$ concentrations only induce the partial discharge of permeabilized hepatocyte calcium pools because of the concomitant reduction of intraluminal Ca^{2+} concentration? FEBS Lett. *301*, 287–290.

Dawson, A.P., Lea, E.J., and Irvine, R.F. (2003). Kinetic model of the inositol trisphosphate receptor that shows both steady-state and quantal patterns of Ca^{2+} release from intracellular stores. Biochem. J. *370*, 621–629.

Ding, Z., Rossi, A.M., Riley, A.M., Rahman, T., Potter, B.V.L., and Taylor, C.W. (2010). Binding of inositol 1,4,5-trisphosphate (IP_3) and adenophostin A to the N-terminal region of the IP_3 receptor: Thermodynamic analysis using fluorescence polarization with a novel IP_3 receptor ligand. Mol. Pharmacol. *77*, 995–1004.

Dohle, W., Su, X., Mills, S.J., Rossi, A.M., Taylor, C.W., and Potter, B.V.L. (2019). A synthetic cyclitol-nucleoside conjugate polyphosphate is a highly potent second messenger mimic. Chem. Sci. (Camb.) *10*, 5382–5390.

Dupont, G., Combettes, L., Bird, G.S., and Putney, J.W. (2011). Calcium oscillations. Cold Spring Harb. Perspect. Biol. *3*, a004226.

Ferrell, J.E., Jr. (2016). Perfect and near-perfect adaptation in cell signaling. Cell Syst. *2*, 62–67.

Ferris, C.D., Cameron, A.M., Huganir, R.L., and Snyder, S.H. (1992). Quantal calcium release by purified reconstituted inositol 1,4,5-trisphosphate receptors. Nature *356*, 350–352.

Hajnóczky, G., and Thomas, A.P. (1994). The inositol trisphosphate calcium channel is inactivated by inositol trisphosphate. Nature *370*, 474–477.

Hajnóczky, G., Lin, C., and Thomas, A.P. (1994). Luminal communication between intracellular calcium stores modulated by GTP and the cytoskeleton. J. Biol. Chem. *269*, 10280–10287.

Hannaert-Merah, Z., Coquil, J.-F., Combettes, L., Claret, M., Mauger, J.-P., and Champeil, P. (1994). Rapid kinetics of myo-inositol trisphosphate binding and dissociation in cerebellar microsomes. J. Biol. Chem. *269*, 29642–29649.

Hill, T.D., Dean, N.M., and Boynton, A.L. (1988). Inositol 1,3,4,5-tetrakisphosphate induces Ca^{2+} sequestration in rat liver cells. Science *242*, 1176–1178.

Hirose, K., and Iino, M. (1994). Heterogeneity of channel density in inositol-1,4,5-trisphosphate-sensitive Ca^{2+} stores. Nature *372*, 791–794.

Ionescu, L., Cheung, K.H., Vais, H., Mak, D.O., White, C., and Foskett, J.K. (2006). Graded recruitment and inactivation of single InsP3 receptor Ca2+-release channels: implications for quantal [corrected] Ca^{2+} release. J. Physiol. *573*, 645–662.

Irvine, R.F. (1990). "Quantal" Ca^{2+} release and the control of Ca^{2+} entry by inositol phosphates—A possible mechanism. FEBS Lett. *263*, 5–9.

Iwai, M., Tateishi, Y., Hattori, M., Mizutani, A., Nakamura, T., Futatsugi, A., Inoue, T., Furuichi, T., Michikawa, T., and Mikoshiba, K. (2005). Molecular cloning of mouse type 2 and type 3 inositol 1,4,5-trisphosphate receptors and identification of a novel type 2 receptor splice variant. J. Biol. Chem. *280*, 10305–10317.

Lampe, D., Liu, C., and Potter, B.V.L. (1994). Synthesis of selective non-Ca^{2+}-mobilizing inhibitors of D-myo-inositol 1,4,5-trisphosphate 5-phosphatase. J. Med. Chem. *37*, 907–912.

Lytton, J., Westlin, M., Burk, S.E., Shull, G.E., and MacLennan, D.H. (1992). Functional comparisons between isoforms of the sarcoplasmic or endoplasmic reticulum family of calcium pumps. J. Biol. Chem. *267*, 14483–14489.

Mak, D.-O., and Foskett, J.K. (1997). Single-channel kinetics, inactivation, and spatial distribution of inositol trisphosphate (IP_3) receptors in *Xenopus* oocyte nucleus. J. Gen. Physiol. *109*, 571–587.

Mak, D.-O., McBride, S., Raghuram, V., Yue, Y., Joseph, S.K., and Foskett, J.K. (2000). Single-channel properties in endoplasmic reticulum membrane of recombinant type 3 inositol trisphosphate receptor. J. Gen. Physiol. *115*, 241–256.

Mak, D.-O., McBride, S., and Foskett, J.K. (2001). Regulation by Ca^{2+} and inositol 1,4,5-trisphosphate (InsP3) of single recombinant type 3 InsP3 receptor channels. Ca^{2+} activation uniquely distinguishes types 1 and 3 insp3 receptors. J. Gen. Physiol. *117*, 435–446.

Marchant, J.S., and Taylor, C.W. (1997). Cooperative activation of IP_3 receptors by sequential binding of IP_3 and Ca^{2+} safeguards against spontaneous activity. Curr. Biol. *7*, 510–518.

Marchant, J.S., and Taylor, C.W. (1998). Rapid activation and partial inactivation of inositol trisphosphate receptors by inositol trisphosphate. Biochemistry *37*, 11524–11533.

Marshall, I.C.B., and Taylor, C.W. (1994). Two calcium-binding sites mediate the interconversion of liver inositol 1,4,5-trisphosphate receptors between three conformational states. Biochem. J. *301*, 591–598.

Mataragka, S., and Taylor, C.W. (2018). All three IP_3 receptor subtypes generate Ca^{2+} puffs, the universal building blocks of IP_3-evoked Ca^{2+} signals. J. Cell Sci. *131*, jcs220848.

Meyer, T., and Stryer, L. (1988). Molecular model for receptor-stimulated calcium spiking. Proc. Natl. Acad. Sci. USA *85*, 5051–5055.

Meyer, T., and Stryer, L. (1990). Transient calcium release induced by successive increments of inositol 1,4,5-trisphosphate. Proc. Natl. Acad. Sci. USA *87*, 3841–3845.

Missiaen, L., De Smedt, H., Droogmans, G., and Casteels, R. (1992). Ca^{2+} release induced by inositol 1,4,5-trisphosphate is a steady-state phenomenon controlled by luminal Ca^{2+} in permeabilized cells. Nature *357*, 599–602.

Muallem, S., Pandol, S.J., and Beeker, T.G. (1989). Hormone-evoked calcium release from intracellular stores is a quantal process. J. Biol. Chem. *264*, 205–212.

Nash, M.S., Young, K.W., Challiss, R.A.J., and Nahorski, S.R. (2001). Intracellular signalling. Receptor-specific messenger oscillations. Nature *413*, 381–382.

Oldershaw, K.A., Nunn, D.L., and Taylor, C.W. (1991). Quantal Ca^{2+} mobilization stimulated by inositol 1,4,5-trisphosphate in permeabilized hepatocytes. Biochem. J. *278*, 705–708.

Oldershaw, K.A., Richardson, A., and Taylor, C.W. (1992). Prolonged exposure to inositol 1,4,5-trisphosphate does not cause intrinsic desensitization of the intracellular Ca^{2+}-mobilizing receptor. J. Biol. Chem. *267*, 16312–16316.

Pantazaka, E., and Taylor, C.W. (2011). Differential distribution, clustering, and lateral diffusion of subtypes of the inositol 1,4,5-trisphosphate receptor. J. Biol. Chem. *286*, 23378–23387.

Parker, I., and Ivorra, I. (1990). Localized all-or-none calcium liberation by inositol trisphosphate. Science *250*, 977–979.

Parys, J.B., Missiaen, L., De Smedt, H., and Casteels, R. (1993). Loading dependence of inositol 1,4,5-trisphosphate-induced Ca^{2+} release in the clonal cell line A7r5. Implications for the mechanism of quantal Ca^{2+} release. J. Biol. Chem. *268*, 25206–25212.

Prins, D., and Michalak, M. (2011). Organellar calcium buffers. Cold Spring Harb. Perspect. Biol. *3*, 004069.

Qin, F. (2004). Restoration of single-channel currents using the segmental k-means method based on hidden Markov modeling. Biophys. J. *86*, 1488–1501.

Renard-Rooney, D.C., Hajnóczky, G., Seitz, M.B., Schneider, T.G., and Thomas, A.P. (1993). Imaging of inositol 1,4,5-trisphosphate-induced Ca^{2+} fluxes in single permeabilized hepatocytes. Demonstration of both quantal and nonquantal patterns of Ca^{2+} release. J. Biol. Chem. *268*, 23601–23610.

Richardson, A., and Taylor, C.W. (1993). Effects of Ca^{2+} chelators on purified inositol 1,4,5-trisphosphate ($InsP_3$) receptors and $InsP_3$-stimulated Ca^{2+} mobilization. J. Biol. Chem. *268*, 11528–11533.

Riley, A.M., Dozol, H., Spiess, B., and Potter, B.V.L. (2004). 2-*O*-(2-aminoethyl)-myo-inositol 1,4,5-trisphosphate as a novel ligand for conjugation: physicochemical properties and synthesis of a new Ins(1,4,5)P(3) affinity matrix. Biochem. Biophys. Res. Commun. *318*, 444–452.

Ríos, E. (2018). Calcium-induced release of calcium in muscle: 50 years of work and the emerging consensus. J. Gen. Physiol. *150*, 521–537.

Rossi, A.M., and Taylor, C.W. (2020). Reliable measurement of free Ca^{2+} concentrations in the ER lumen using Mag-Fluo-4. Cell Calcium *87*, 102188.

Rossi, A.M., Riley, A.M., Tovey, S.C., Rahman, T., Dellis, O., Taylor, E.J.A., Veresov, V.G., Potter, B.V.L., and Taylor, C.W. (2009). Synthetic partial agonists reveal key steps in IP_3 receptor activation. Nat. Chem. Biol. *5*, 631–639.

Saleem, H., Tovey, S.C., Molinski, T.F., and Taylor, C.W. (2014). Interactions of antagonists with subtypes of inositol 1,4,5-trisphosphate (IP_3) receptor. Br. J. Pharmacol. *171*, 3298–3312.

Schindelin, J., Arganda-Carreras, I., Frise, E., Kaynig, V., Longair, M., Pietzsch, T., Preibisch, S., Rueden, C., Saalfeld, S., Schmid, B., et al. (2012). Fiji: An open-source platform for biological-image analysis. Nat. Methods *9*, 676–682.

Shaw, G., Morse, S., Ararat, M., and Graham, F.L. (2002). Preferential transformation of human neuronal cells by human adenoviruses and the origin of HEK 293 cells. FASEB J. *16*, 869–871.

Shen, D., Wang, X., Li, X., Zhang, X., Yao, Z., Dibble, S., Dong, X.P., Yu, T., Lieberman, A.P., Showalter, H.D., and Xu, H. (2012). Lipid storage disorders block lysosomal trafficking by inhibiting a TRP channel and lysosomal calcium release. Nat. Commun. *3*, 731.

Shuttleworth, T.J. (1992). Ca^{2+} release from inositol trisphosphate-sensitive stores is not modulated by intraluminal $[Ca^{2+}]$. J. Biol. Chem. *267*, 3573–3576.

Smith, I.F., and Parker, I. (2009). Imaging the quantal substructure of single IP_3R channel activity during Ca^{2+} puffs in intact mammalian cells. Proc. Natl. Acad. Sci. USA *106*, 6404–6409.

Stehno-Bittel, L., Lückhoff, A., and Clapham, D.E. (1995). Calcium release from the nucleus by $InsP_3$ receptor channels. Neuron *14*, 163–167.

Stephenson, R.P. (1956). A modification of receptor theory. Br. J. Pharmacol. Chemother. *11*, 379–393.

Subramanian, K., and Meyer, T. (1997). Calcium-induced restructuring of nuclear envelope and endoplasmic reticulum calcium stores. Cell *89*, 963–971.

Sugawara, H., Kurosaki, M., Takata, M., and Kurosaki, T. (1997). Genetic evidence for involvement of type 1, type 2 and type 3 inositol 1,4,5-trisphosphate receptors in signal transduction through the B-cell antigen receptor. EMBO J. *16*, 3078–3088.

Supattapone, S., Worley, P.F., Baraban, J.M., and Snyder, S.H. (1988). Solubilization, purification, and characterization of an inositol trisphosphate receptor. J. Biol. Chem. *263*, 1530–1534.

Suzuki, J., Kanemaru, K., Ishii, K., Ohkura, M., Okubo, Y., and Iino, M. (2014). Imaging intraorganellar Ca^{2+} at subcellular resolution using CEPIA. Nat. Commun. *5*, 4153.

Suzuki, J., Kanemaru, K., and Iino, M. (2016). Genetically encoded fluorescent indicators for organellar calcium imaging. Biophys. J. *111*, 1119–1131.

Taufiq-Ur-Rahman, Skupin, A., Falcke, M., and Taylor, C.W. (2009). Clustering of InsP3 receptors by InsP3 retunes their regulation by InsP3 and Ca^{2+}. Nature *458*, 655–659.

Taylor, C.W., and Potter, B.V.L. (1990). The size of inositol 1,4,5-trisphosphate-sensitive Ca^{2+} stores depends on inositol 1,4,5-trisphosphate concentration. Biochem. J. *266*, 189–194.

Thillaiappan, N.B., Chavda, A.P., Tovey, S.C., Prole, D.L., and Taylor, C.W. (2017). Ca^{2+} signals initiate at immobile IP_3 receptors adjacent to ER-plasma membrane junctions. Nat. Commun. *8*, 1505.

Thillaiappan, N.B., Smith, H.A., Atakpa-Adaji, P., and Taylor, C.W. (2021). KRAP tethers IP_3 receptors to actin and licenses them to evoke cytosolic Ca^{2+} signals. Nat. Commun. *12*, 4514.

Tovey, S.C., Sun, Y., and Taylor, C.W. (2006). Rapid functional assays of intracellular Ca^{2+} channels. Nat. Protoc. *1*, 259–263.

Tregear, R.T., Dawson, A.P., and Irvine, R.F. (1991). Quantal release of Ca2+ from intracellular stores by InsP3: tests of the concept of control of Ca2+ release by intraluminal Ca2+. Proc. Biol. Sci. *243*, 263–268.

Tveito, A., and Lines, G.T. (2010). Computing Characterizations of Drugs for Ion Channels and Receptors Using Markov Models (SpringerOpen).

Vais, H., Foskett, J.K., and Mak, D.O. (2010). Unitary Ca^{2+} current through recombinant type 3 $InsP_3$ receptor channels under physiological ionic conditions. J. Gen. Physiol. *136*, 687–700.

Vais, H., Foskett, J.K., Ullah, G., Pearson, J.E., and Mak, D.O. (2012). Permeant calcium ion feed-through regulation of single inositol 1,4,5-trisphosphate receptor channel gating. J. Gen. Physiol. *140*, 697–716.

Vais, H., Wang, M., Mallilankaraman, K., Payne, R., McKennan, C., Lock, J.T., Spruce, L.A., Fiest, C., Chan, M.Y., Parker, I., et al. (2020). ER-luminal [Ca^{2+}] regulation of $InsP_3$ receptor gating mediated by an ER-luminal peripheral Ca^{2+}-binding protein. eLife *9*, e53531.

Valm, A.M., Cohen, S., Legant, W.R., Melunis, J., Hershberg, U., Wait, E., Cohen, A.R., Davidson, M.W., Betzig, E., and Lippincott-Schwartz, J. (2017). Applying systems-level spectral imaging and analysis to reveal the organelle interactome. Nature *546*, 162–167.

Venkatachalam, K., Hofmann, T., and Montell, C. (2006). Lysosomal localization of TRPML3 depends on TRPML2 and the mucolipidosis-associated protein TRPML1. J. Biol. Chem. *281*, 17517–17527.

Wang, S.Q., Stern, M.D., Ríos, E., and Cheng, H. (2004). The quantal nature of Ca^{2+} sparks and in situ operation of the ryanodine receptor array in cardiac cells. Proc. Natl. Acad. Sci. USA *101*, 3979–3984.

Wiltgen, S.M., Dickinson, G.D., Swaminathan, D., and Parker, I. (2014). Termination of calcium puffs and coupled closings of inositol trisphosphate receptor channels. Cell Calcium *56*, 157–168.

Winding, P., and Berchtold, M.W. (2001). The chicken B cell line DT40: A novel tool for gene disruption experiments. J. Immunol. Methods *249*, 1–16.

Wojcikiewicz, R.J.H. (1995). Type I, II, and III inositol 1,4,5-trisphosphate receptors are unequally susceptible to down-regulation and are expressed in markedly different proportions in different cell types. J. Biol. Chem. *270*, 11678–11683.

Wojcikiewicz, R.J.H. (2018). The making and breaking of inositol 1,4,5-trisphosphate receptor tetramers. Messenger (Los Angel.) *6*, 45–49.

Yamashita, M. (2006). "Quantal" Ca($^{2+}$) release reassessed—A clue to oscillation and synchronization. FEBS Lett. *580*, 4979–4983.

Yamashita, M., Sugioka, M., and Ogawa, Y. (2006). Voltage- and Ca^{2+}-activated potassium channels in Ca^{2+} store control Ca^{2+} release. FEBS J. *273*, 3585–3597.

Yoshikawa, F., Iwasaki, H., Michikawa, T., Furuichi, T., and Mikoshiba, K. (1999). Cooperative formation of the ligand-binding site of the inositol 1,4, 5-trisphosphate receptor by two separable domains. J. Biol. Chem. *274*, 328–334.

STAR★METHODS

KEY RESOURCES TABLE

REAGENT or RESOURCE	SOURCE	IDENTIFIER
Antibodies		
Anti-IP_3R Ab (AbC, recognizes all vertebrate IP_3R subtypes, rabbit, monoclonal) (1:1000)	Cell Signaling Technology (Danvers, MA, USA)	Cat#8568S<br>Clone # D53A5<br>RRID: AB_10890699
Anti-β-actin Ab (mouse monoclonal) (1:30000)	Cell Signaling Technology	Cat#3700S<br>Clone # 8H10D10<br>RRID: AB_2242334
Anti-erlin 2 Ab (rabbit, monoclonal) (1:1000)	Abcam (Cambridge, UK)	Cat#ab129207<br>Clone # EPR8088<br>RRID:AB_11143745
Anti-mouse secondary Ab (mouse IgG κ-binding protein conjugated to horseradish peroxidise, HRP) (1:5000)	Santa Cruz (Dallas, TX, USA)	Cat#sc-516102<br>RRID: AB_2687626
Anti-rabbit secondary Ab (goat monoclonal, conjugated to HRP) (1:5000)	SeraCare (Milford, MA, USA)	Cat#5220-0336<br>RRID: AB_2857917
Chemicals, peptides, and recombinant proteins		
2-aminoethoxy diphenyl borate (2-APB)	BioVision (Milpitas, CA, USA)	Cat#1798-100
ATP	Sigma-Aldrich (St. Louis, MO, USA)	Cat#A6419
Bafilomycin A_1	Fluorochem (Hadfield, UK).	Cat#M01404
BAPTA	Phion (Dorset, UK)	Cat#61061782
Bis(4-nitrophenyl) carbonate	Acros Organics via ThermoFisher (Waltham, MA, USA)	Cat#171600250
Cbz_3-spermine-IP_3 (**2**)	Synthesis and properties described in this paper	Figure 2A
Chelex-100 resin Na^+ form	Sigma-Aldrich	Cat#C7901
Cyclopiazonic acid (CPA)	Tocris (Bristol, UK)	Cat#1235
Dequalinium chloride hydrate	Sigma-Aldrich	Cat#D3768
D-*myo*-inositol 1,4,5-trisphosphate (IP_3)	Enzo (Exeter, UK)	Cat#ALX-307-007-M005
D-*myo*-[^{3}H]inositol 1,4,5-trisphosphate ([^{3}H] IP_3) (19.3-21 Ci/mmol)	PerkinElmer (Beaconsfield, UK)	Cat#NET911005UC
D-*myo*-inositol 2,4,5-trisphosphate ((2,4,5)IP_3)	Cayman Chemical (Ann Arbor, MI, USA)	Cat#10007779
Dulbecco's modified Eagle's medium/ nutrient mixture F-12 with GlutaMAX (DMEM/F-12 GlutaMAX)	ThermoFisher	***Cat#10565018***
Foetal bovine serum (FBS)	Sigma	Cat#F7524
G418	Formedium (Norfolk, UK)	Cat#G4181
Heparin, sodium salt	Calbiochem via Merck (Watford, UK)	Cat#375095
Ionomycin, Ca^{2+} salt	Apollo Scientific (Stockport, UK)	Cat#BII0123
LiChroprep RP-18 (25-40 μm)	Merck	Cat#109303
Mag-fluo 4 AM	ThermoFisher	Cat#M14206
ML-SA1	Merck	Cat#648493
Poly-L-lysine (1% solution)	Sigma	Cat#P8920
Q-Sepharose Fast Flow resin	GE Healthcare (Chicago, IL, USA)	Cat#GE17-0510-01
Saponin	Sigma-Aldrich	Cat#S4521

(Continued on next page)

REAGENT or RESOURCE	SOURCE	IDENTIFIER
Continued		
Thapsigargin	Bio-Techne (Middlesex, UK)	Cat#1138/1
Triethylamine	Sigma-Aldrich	Cat#90340
**Critical commercial assays**		
8-well glass-bottom μ-slides	Ibidi (Munich, Germany)	Cat#80827
35-mm glass bottom dishes (#1 glass)	Cellvis (Mountain View, CA 94039)	Cat#D35-14-1-N
ECL-Prime/Select western blotting reagents	Amersham via GE Healthcare	Cat#RPN2236/RPN2235
Half-area 96-well black-walled plates	Greiner, Bio-One (Stonehouse, UK)	Cat#675090
Neon transfection system kit	ThermoFisher	Cat#MPK10096
iBlot gel-transfer stacks PVDF	ThermoFisher	Cat#IB401001
Protease inhibitor cocktail (Roche)	Merck	Cat#11836153001
NuPAGE, SDS-PAGE gels (3-8% Tris-acetate or 4-12% Bis-Tris)	ThermoFisher	Cat#EA0375BOX or NP0321BOX
TransIT-LT1 transfection reagent	Geneflow (Lichfield, UK)	Cat#MIR 2305
**Experimental models: Cell lines**		
Gallus gallus DT40-IP$_3$R1 (DT40-IP$_3$R2; DT40-IP$_3$R3) cells	Tovey et al., 2006	N/A
Homo sapiens HEK-3KO cells	Kerafast (Boston, MA, USA) (Alzayady et al., 2016)	Cat#EUR030
Homo sapiens HEK-G-CEPIA*er* cells	This paper	N/A
Homo sapiens wild-type HEK293 cells	Dr D Yule, University of Rochester, NY, USA	N/A
**Oligonucleotides**		
siRNA targeting sequence of Erlin 2 (siRNA 1) ATCTACTTTGACAGAATTGAA	QIAGEN (Manchester, UK)	Cat#SI04952689
siRNA targeting sequence of Erlin 2 (siRNA 2) AAGATAGAAGAGGGACATATT	QIAGEN	Cat#SI04952696
Non-silencing (NS) siRNA	QIAGEN	Cat#1027280
**Recombinant DNA**		
CFP-TRPML1 plasmid	Addgene (Venkatachalam et al., 2006)	Cat#18827
G-CEPIA1*er* plasmid	Addgene (Suzuki et al., 2016)	Cat#58215
IP$_3$R1 in pcDNA3.1(-) /Myc-His B plasmid	Dohle et al., 2019	N/A
EGFP-IP$_3$R1 in pcDNA3.2/DEST	Pantazaka and Taylor, 2011	N/A
Residues 1-604 of rat IP$_3$R1 (NT) in pTrcHisA plasmid	Rossi et al., 2009	N/A
Residues 224-604 of rat IP$_3$R1 (IBC) in pTrcHisA plasmid	Rossi et al., 2009	N/A
**Software and algorithms**		
Fiji		Schindelin et al., 2012
MetaMorph. Version 7.10.1.161	Molecular Devices (San Jose, CA, USA)	N/A
PRISM. Version 8.4.2	GraphPad Software (La Jolla, CA, USA)	N/A
QuB	Qin, 2004	N/A
SoftMax Pro. Version 5.4	Molecular Devices	N/A
Codes for simulations	This paper	Available from: https://github.com/genedupont/Quantal

RESOURCE AVAILABILITY

Lead contact

Requests for resources and reagents should be directed to the Lead Contact, Colin W. Taylor (cwt1000@cam.ac.uk).

CellPress
OPEN ACCESS

Materials availability

All plasmids used are commercially available, except EGFP-IP_3R1 which is available from the Lead Contact. The cell lines generated in this study are available from the Lead Contact. Compound **2** is available from Barry V. L. Potter (barry.potter@pharm.ox.ac.uk).

Data and code availability

No large datasets were generated by this study. The computer code used for the simulations is available at: https://github.com/genedupont/Quantal. Any additional information required to reanalyze the data reported in this paper is available from the lead contact upon request.

EXPERIMENTAL MODEL AND SUBJECT DETAILS

Cells

DT40 cells are an avian B cell line, wherein high rates of DNA recombination have been widely exploited for analyses of cell signaling and B cell function (Winding and Berchtold, 2001). DT40 cells were the first cells in which all three IP_3R subtypes were genetically disrupted to provide a null-background for IP_3R expression (Sugawara et al., 1997). We used DT40 cells without native IP_3Rs to generate cell lines stably expressing single subtypes of mammalian IP_3R (DT40-IP_3R1-3) (Tovey et al., 2006).

Human embryonic kidney 293 (HEK293 cells) are hypotriploid cells derived from embryonic kidney and immortalized by transformation with adenovirus (Shaw et al., 2002). We used wild-type HEK cells (which express all three IP_3R subtypes) (Mataragka and Taylor, 2018), and a stable cell line in which CRISPR/Cas9 was used to prevent expression of all native IP_3Rs (HEK-3KO) (Alzayady et al., 2016). HEK-3KO cells were transfected with mammalian IP_3R1 to generate a monoclonal HEK-IP_3R1 cell line, and wild-type HEK cells were used to generate HEK-G-CEPIA1*er* cells stably expressing the ER-targeted genetically-encoded Ca^{2+} indicator, G-CEPIA1*er*.

The identity of the parental HEK293 cell line used to generate HEK-3KO cells was confirmed by genotyping (Alzayady et al., 2016), and we confirmed the absence of all IP_3Rs by western blotting (Mataragka and Taylor, 2018). None of the other cell lines was authenticated. All cells were periodically (every 2 months) confirmed to be free of mycoplasma.

METHOD DETAILS

Synthesis of 2

We originally synthesized **2** (2-*O*-(1,5,10-tris(benzyloxycarbonyl)-15-oxo-1,5,10,14,16-pentaazaoctadecan-18-yl)-1D-*myo*-inositol 1,4,5-trisphosphate, Figure 2A) as an intermediate in the synthesis of a novel polyamine-IP_3 conjugate, but **2** proved to be most useful for our present purposes. Bis(4-nitrophenyl) carbonate was recrystallized from CH_2Cl_2/hexane. D-2-*O*-(2-aminoethyl)-IP_3 was synthesized as previously reported (Riley et al., 2004) and used as the triethylammonium salt. N^1,N^5,N^{10}-tri(benzyloxycarbonyl)spermine was synthesized according to (Blagbrough and Geall, 1998). Triethylammonium bicarbonate (TEAB) buffer was made by bubbling CO_2 through a chilled aqueous solution of triethylamine. Anion-exchange chromatography was carried out on Q-Sepharose Fast Flow resin (GE Healthcare) using a Pharmacia Biotech Gradifrac system and P-1 pump, eluting at 5 mL/min with a linear gradient (0 to 100%) of 2.0 M aqueous TEAB buffer, collecting 10-mL fractions. Phosphate-containing fractions were identified using a modification of the Briggs phosphate test (Lampe et al., 1994). Reverse-phase ion-pair chromatography was carried out on LiChroprep RP-18 (25-40 μm, Merck) using a BioLogic LP system (BioRad), eluting at 5 mL/min with a linear gradient (0 to 70%) of CH_3CN in 0.05 M TEAB buffer, collecting 7-mL fractions. Compound **2** was accurately quantified using the Ames phosphate assay (Ames and Dubin, 1960). All water used was MilliQ quality.

To a solution of bis(4-nitrophenyl) carbonate (200 mg, 0.66 mmol) in dry CH_2Cl_2 (5 mL) under N_2 was added a solution of N^1,N^5,N^{10}-tri(benzyloxycarbonyl)spermine (400 mg, 0.66 mmol) in dry CH_2Cl_2, dropwise over 10 min. The solution was stirred for 1 hr, after which TLC (CH_2Cl_2/MeOH 10:1 v/v) showed that all the amine (streak, R_f 0.06) had been consumed, with appearance of a product at R_f 0.72 (yellow on heating and stains with phosphomolybdic acid), together with 4-nitrophenol (R_f 0.5) and a trace of unreacted bis(4-nitrophenyl) carbonate (R_f 0.86). CH_2Cl_2 (20 mL) was added, and the solution was washed with saturated aqueous $NaHCO_3$ (3 × 20 mL). The pale yellow organic layer was dried ($MgSO_4$) and concentrated to give a yellow oil, which was purified by flash chromatography (CH_2Cl_2, then CH_2Cl_2:MeOH, 50:1 v/v) to give 4-nitrophenyl *N*-alkylcarbamate (436 mg) as a colorless oil; TLC R_f 0.24 (CH_2Cl_2/EtOAc, 5:1). A solution of this alkylcarbamate (46 mg, 60 μmol) in CD_3OD (0.75 mL) was added to solid 2-*O*-(2-aminoethyl)-IP_3 (triethylammonium salt, 21 mg, 28 μmol) under N_2 in a 5-mL round-bottom flask, followed by dry triethylamine (40 μL). A deep yellow color (4-nitrophenol) appeared within seconds. The clear yellow solution was stirred at room temperature for 2 hr, then transferred to an NMR tube. The ^{31}P NMR spectrum of this solution showed three major peaks (1.01, 3.17 and 4.18 ppm) corresponding to **2**, together with other, smaller peaks. The solution was left for 16 hr in the NMR tube, after which a second ^{31}P NMR spectrum showed only the three product peaks. The reaction was therefore judged to be complete. The contents of the NMR tube were transferred to a 100-mL round-bottom flask and the NMR tube was rinsed with MeOH (1 mL). To the combined washings was added triethylammonium bicarbonate (TEAB) buffer (0.05 M, pH 7.6, 75 mL), and the resulting cloudy yellow solution was left for 16 hr at room temperature to hydrolyse any unreacted *N*-alkylcarbamate. The solution was then loaded onto a column of Q-Sepharose Fast Flow resin (120 mm × 16 mm, bicarbonate form). The column was washed well with

water (200 mL) and then eluted with a gradient of TEAB (2.0 M, 0 to 100%). At approx. 40% 2.0 M TEAB, the column eluent took on an intense yellow color (4-nitrophenol) and this buffer concentration was held constant until the eluent became colorless. When the concentration of TEAB was then allowed to increase, the target compound eluted above 90% 2.0 M TEAB. Fractions containing the target compound were identified using the Briggs phosphate assay, combined and concentrated under reduced pressure to give a colorless glassy residue (40 mg). ^{1}H NMR spectroscopy of the product at this stage showed that it contained significant amounts of alkylammonium contaminants, resulting from the high concentration of TEAB buffer needed to elute it from the column. It was therefore purified further using reverse-phase ion-pair chromatography: the 40 mg of material was taken up in 0.05 M TEAB (5 mL) and loaded onto a column of LiChroprep RP-18 resin (100 mm × 16 mm). The column was washed well with 0.05 M TEAB, and then eluted with a gradient of CH_3CN in 0.05 M TEAB (0 to 70% CH_3CN). The target eluted at 33%–40% CH_3CN, as detected by UV absorption at 254 nm. Fractions containing the target compound were combined and concentrated under reduced pressure. MeOH was repeatedly added and evaporated until a colorless glass remained (~35 mg). Finally, this material was dissolved in water (5 mL) and passed through a column of Chelex-100 resin (Na^+ form, 70 mm deep in a Pasteur pipette) to remove triethylammonium ions. The column was washed with water (5 mL) and the combined eluents were lyophilised to give **2** as a white, fluffy solid (28 mg, 19.7 μmol, 70% as determined by total phosphate assay); ^{1}H NMR (Na^+ salt in D_2O, 400 MHz) δ 1.16–1.48 (4H, broad m, 2 × spermine CH_2), 1.49–1.80 (4H, broad m, 2 × spermine CH_2), 2.90–3.32 (12H, broad m, 6 × spermine CH_2), 3.39 (2H, broad s, NCH_2CH_2O), 3.80 (1H, broad d, *J* 9.9 Hz, inositol H-3), 3.87–4.13 (5H, m, inositol H-1, H-5. H-6 and NCH_2CH_2O), 4.15 (1H, broad s, inositol H-2), 4.33 (1H, q, *J* 9.1 Hz, inositol H-4), 4.92–5.14 (6H, m, 3 × OCH_2Ph), 7.14–7.45 (15H, m, Ph); ^{13}C NMR (Na^+ salt in D_2O, 126 MHz) δ 24.42, 29.96, 27.35 and 28.02 (spermine CH_2), 37.40 and 38.01 (spermine CH_2), 40.20 (NCH_2CH_2O), 44.04, 45.01 and 46.40 (spermine CH_2), 66.51 and 67.20 (3 × OCH_2Ph), 71.38 (inositol C-3), 71.91 (inositol C-6), 72.72 (NCH_2CH_2O), 74.59 (inositol C-1), 76.46 (inositol C-4), 78.31 (inositol C-5), 79.69 (inositol C-2), 127.53–128.58 (CH of Ph), 136.27 and 136.60 (*ipso*-C of Ph), 157.21, 157.37 and 157.82 (3 × urethane C = O), 160.56 (urea C = O); ^{31}P NMR (triethylammonium salt in CD_3OD, 162 MHz, ^{1}H-decoupled) δ 0.81 (1 P), 1.55 (1 P) and 2.50 (1 P); HRMS (*m/z*) $[M–H]^-$ calcd for $C_{43}H_{62}N_5O_{22}P_3$, 1092.3027; found 1092.3017.

Cell culture and transfections

Methods used to generate and culture DT40 cells without native IP_3Rs and then transfected to stably express a single subtype of mammalian IP_3R (DT40-IP_3R1-3) were described previously (Tovey et al., 2006). HEK293 cells were cultured in DMEM/F-12 GlutaMAX medium with 10% FBS at 37°C in 95% air and 5% CO_2. Cells were passaged or used for experiments when they reached confluence. Plasmids were transfected into HEK cells using TransIT-LT1 reagent. The transfection efficiency of HEK cells transiently expressing CFP-TRPML1 or EGFP-IP_3R1 was ~60%, assessed by CFP or EGFP fluorescence. For double transfections (CFP-TRPML1 and EGFP-IP_3R1), the transfection efficiency was ~15%. To generate HEK cells expressing only IP_3R1 (HEK-IP_3R1 cells), HEK-3KO cells were transfected with the gene encoding rat IP_3R1 (lacking the S1 splice site) (Rossi et al., 2009) cloned into pcDNA3.1(-)/Myc-His B plasmid (Dohle et al., 2019). To produce HEK cells stably expressing G-CEPIA1*er* (HEK-G-CEPIA1*er* cells), wild-type HEK cells were transfected with G-CEPIA1*er* plasmid (Suzuki et al., 2014). To generate stable cell lines (HEK-IP_3R1 and HEK-G-CEPIA1*er* cells), cells were passaged 48 hr after transfection in medium with G418 (1 mg/mL). Selection was maintained for 2 weeks with medium changes every 3 days. Monoclonal HEK-IP_3R1 cell lines were selected by plating cells (~1 cell/well) into 96-well plates in medium containing G418 (1 mg/mL). After 4 days, wells with only one cell were identified, cells were then grown to confluence, and cell lines were expanded and their expression of IP_3R1 was confirmed by western blot using an antibody specific for IP_3R1 (Rossi et al., 2009). To obtain polyclonal HEK-G-CEPIA1*er* cells, cells with the highest G-CEPIA1*er* expression (~1%) were isolated using fluorescence-activated cell sorting (FACS). siRNA transfections of wild-type HEK cells used a Neon transfection system with a final siRNA concentration of 150 nM. Cells were incubated for 48 hr before use. The siRNAs used are listed in the Key Resources table.

Ca^{2+} release by IP_3Rs

Incubation of cells with the acetoxymethylester (AM) of Mag-fluo 4 traps low-affinity forms of the indicator (K_d^{Ca} = 1.15 mM measured *in situ*) within the ER. Fluorescence, which is approximately linearly related to ER free $[Ca^{2+}]$ (since $[Ca^{2+}]_{ER} < K_d^{Ca}$), can then be used, without further calibration, to reliably report free $[Ca^{2+}]$ within the ER lumen (Rossi and Taylor, 2020). The ER of DT40 or HEK293 cells was loaded with indicator by incubating cells with Mag-fluo 4 AM (20 μM, 60 min, 22°C) in HEPES-buffered saline (HBS: 135 mM NaCl, 5.9 mM KCl, 11.6 mM HEPES, 1.5 mM $CaCl_2$, 11.5 mM glucose, 1.2 mM $MgCl_2$, pH 7.3) as described (Rossi et al., 2009). After washing and permeabilization with saponin (10 μg/mL, 37°C, 2-3 min) in Ca^{2+}-free cytosol-like medium (Ca^{2+}-free CLM), cells were centrifuged (650 × *g*, 2 min) and resuspended in Mg^{2+}-free CLM supplemented with $CaCl_2$ to give a final free $[Ca^{2+}]$ of 220 nM after addition of 1.5 mM MgATP. Ca^{2+}-free CLM comprised: 20 mM NaCl, 140 mM KCl, 1 mM EGTA, 20 mM PIPES, 2 mM $MgCl_2$, pH 7.0. Cells (4×10^5 cells/well) were attached to poly-L-lysine-coated 96-well black-walled plates (Greiner Bio-One, Stonehouse, UK), and fluorescence (excitation and emission at 485 nm and 520 nm, respectively) was recorded at intervals of 1.44 s using a FlexStation III plate-reader (Molecular Devices, Sunnyvale, CA, USA). MgATP (1.5 mM) was added to initiate Ca^{2+} uptake, and when the ER had loaded to steady state with Ca^{2+} (~150 s), ligands were added. Responses to the ligands show ER Ca^{2+} contents as either time courses or, in summary results, as the Ca^{2+} content recorded 20 s after addition of the stimulus. The distribution of samples across 96-well plates was varied between experiments to avoid any systematic 'position-related' artifacts. HEK-G-CEPIA1*er* cells were permeabilized and assayed as described for Mag-fluo 4-loaded cells.

Fluorescence microscopy

Fluorescence microscopy imaging was performed using an inverted Olympus IX83 microscope equipped with either a 60 × oil-immersion objective (numerical aperture, NA 1.45) or a 100 × oil-immersion total internal reflection fluorescence (TIRF) objective (NA 1.49), a multi-line laser bank (excitation light 425 nm and 488 nm) and an iLas2 targeted laser illumination system (Cairn, Faversham, Kent, UK). Excitation light was transmitted through either a quad dichroic beam splitter (TRF89902-QUAD) or a dichroic mirror (for 425 nm; ZT442rdc-UF2) (Chroma). Emitted light was passed through appropriate filters (Cairn Optospin; peak/bandwidth: 480/40 and 525/50) and detected using an iXon Ultra 897 electron-multiplied charge-coupled device (EMCCD) camera (512 × 512 pixels, Andor). Spinning-disc confocal microscopy used a spinning disc with a 70-μm pinhole (X-Light, Crest Optics). TIRFM used the iLas2 illumination system and the penetration depth was 90-140 nm. For analyses of the distribution of CFP-TRPML1 and EGFP-IP_3R1 (Figure S5F), we confirmed that there was no bleed-through between channels. Image capture and processing used MetaMorph (Molecular Devices) and Fiji (Schindelin et al., 2012). All images were corrected for background (MetaMorph) by subtracting fluorescence detected from a region without cells.

Single-cell analyses of Ca^{2+} release

HEK-G-CEPIA1*er* cells were permeabilized with saponin, and resuspended in Mg^{2+}-free CLM supplemented with $CaCl_2$ to give a final free [Ca^{2+}] of 220 nM after addition of 1.5 mM MgATP. Cells were attached (4×10^5 cells/well) to a poly-L-lysine-coated 8-well glass bottomed μ-slide. MgATP (1.5 mM) was added to initiate Ca^{2+} uptake, and when the ER had loaded to steady state with Ca^{2+} (~700 s), ligands were added.

Cells were imaged (2 s/frame) using spinning-disk confocal microscopy with a 60 × oil-immersion objective. Images were collected using MetaMorph, corrected for background fluorescence and analyzed using the Time Series Analyzer plugin (Fiji). Fluorescence intensity values from regions of interest (ROI) that delineated individual cells or ROI within individual cells are reported in arbitrary fluorescence units.

[^3H]IP_3 binding

IP_3R1, which accounts for ~99% of IP_3Rs in cerebellum (Wojcikiewicz, 1995), was purified from cerebella of adult Wistar rats using heparin-affinity chromatography (Rossi et al., 2009). The purified protein (10 μg/mL) was stored at −80°C in 500 mM NaCl, 50 mM Tris, 10% glycerol, 1 mM 2-mercaptoethanol, 1 mM benzamidine, 1 mM EGTA, 50 mM Tris, 1% CHAPS, pH 8.0. N-terminal fragments of IP_3R1 (IBC, residues 224-604; NT, residues 1-604) were expressed as N-terminally tagged His_6-fusion proteins in *E. coli* and cleaved from the tag with thrombin (Rossi et al., 2009). Most equilibrium-competition binding assays (4°C, 5 min) were performed in Tris-EDTA medium (TEM, 500 μL) comprising 50 mM Tris, 1 mM EDTA, pH 8.3 with [^3H]IP_3 (21 Ci/mmol, 0.3-1.5 nM), bacterial lysate (7 μg protein) or purified IP_3R1 (2 μg protein), and competing ligands (Rossi et al., 2009). Bound and free ligand were separated by centrifugation (4°C, 5 min, 14,000 × *g*). Non-specific binding, determined by addition of 10 μM IP_3, was less than 10% of total binding.

For analyses of [^3H]IP_3 binding under conditions that mimicked those used for functional assays (Figure 2G), equilibrium-competition binding assays (22°C, 5-10 min) were performed in Mg^{2+}-free CLM supplemented with $CaCl_2$ to give a final free [Ca^{2+}] of 220 nM, [^3H]IP_3 (19.3 Ci/mmol, 7.5 nM), membranes from cerebella of adult Wistar rats (~100 μg protein) and competing ligands. Bound and free ligand were separated by centrifugation (22°C, 5 min, 14,000 × *g*). Non-specific binding, determined by addition of 100 μM IP_3, was less than 10% of total binding.

Nuclear patch-clamp recording from IP_3Rs

The methods used for patch-clamp recording from patches excised from the outer nuclear envelope of DT40-IP_3R1 cells, with K^+ as charge-carrier and using QuB (Qin, 2004) for analysis of currents, were as described previously (Taufiq-Ur-Rahman et al., 2009; Rossi et al., 2009). It typically took 30-45 s to establish a recording, and P_o was then stable throughout recordings that typically lasted up to 8-10 mins.

Western blotting

Cells were harvested in radio-immunoprecipitation assay (RIPA) buffer (150 mM NaCl, 1.0% NP-40 or Triton X-100, 0.5% sodium deoxycholate, 0.1% SDS, 50 mM Tris, pH 8.0) with protease inhibitor cocktail. The cell suspension was passed through a 25-gauge needle, incubated for 30 min at 4°C with rotation, sonicated (3 × 10 s pulses on ice), and then cleared by centrifugation (14,000 × *g*, 60 min, 4°C). Proteins were separated using 3%–8% or 4%–12% SDS-PAGE gels (NuPAGE), and transferred to PVDF membranes using an iBlot system. Antibodies are listed in the Key Resources table. Proteins were visualized using ECL Prime/Select western blotting detection reagents and the GeneGnome imaging system (SynGene, Cambridge, UK) and chemiluminescence was quantified using Fiji (Schindelin et al., 2012). For all quantitative analyses, we used bands for which we confirmed, by serial dilution of samples, a linear relationship between protein loading and band intensity.

QUANTIFICATION AND STATISTICAL ANALYSES

Simulations

Simulations first consider the number of open IP_3Rs after sequential additions of IP_3. The scheme assumes that IP_3Rs to which four IP_3 molecules have bound move between three stable states, closed (C_4), open (O_4) and inactivated (I_4), each with similar affinity for IP_3

(Figure 7E). Transitions between receptors states are described by a triangular kinetic scheme (Tveito and Lines, 2010), with $k_1 = 0.4\ s^{-1}$, $k_2 = 0.2\ s^{-1}$ and $k_3 = 0.004\ s^{-1}$, but the scheme includes an irreversible step in a way that is reminiscent of a published model for adaptation in cell signaling (Ferrell, 2016). The transitions between these states (rate constants, k_i, 0.004 - 0.4 s^{-1}, and 100-times slower for k_{-i}) are much slower than the rates of IP_3 association (~$3.5 \times 10^7\ M^{-1}.s^{-1}$; k_{on} ~$3.5\ s^{-1}$ with 100 nM IP_3) (Hannaert-Merah et al., 1994) or dissociation (~28 s^{-1}, estimated from the K_D in Figure 2G). Our scheme therefore assumes that for each IP_3 concentration, there is a fixed number of IP_3Rs with all four IP_3-binding sites occupied, determined by the $K_D^{IP_3}$ (794 nM) and the number of IP_3Rs/cell (49,146 in HEK-IP_3R1 cells) (Figure 7B). IP_3 addition is simulated by an immediate increase in C_4. Simulations are performed stochastically, with each IP_3R simulated independently. The probability of transition i is equal to k_i. Δt. At each time step (12.5 ms), the algorithm generates a number X drawn uniformly at random on the [0,1] interval. If $X < k_i$. Δt, the IP_3R changes state; otherwise it remains in the same state. The behavior of the IP_3R population is obtained by summing the number of IP_3Rs in each state at every time step. The output provides the temporal evolution of the number of open IP_3Rs. The same scheme and parameters were used to simulate the behavior of IP_3Rs within a single wild-type HEK cell, for which we determined the expression level to be 9101 ± 934 IP_3Rs/cell.

To simulate the change in ER free [Ca^{2+}] (C_{ER}) over time, we consider the following equation:

$$\frac{dC_{ER}}{dt} = -(k_{leak} + k_{rel}.O_4).(C_{ER} - C) \quad (1)$$

where O_4 is the number of open IP_3Rs determined from the stochastic simulations, k_{leak} is the rate constant for the IP_3-independent Ca^{2+} leak from the ER ($1.8 \times 10^{-4}\ s^{-1}$, see *Analysis* section), k_{rel} is the rate constant for Ca^{2+} flux through an open IP_3R (0.0008 s^{-1}). We estimated k_{rel} by assuming that the free [Ca^{2+}] within the ER was 500 μM, the buffer capacity was 5 (hence, total ER [Ca^{2+}] = 2.5 mM; see *Analysis* section), and an open IP_3R conducts $5 \times 10^5\ Ca^{2+}\ s^{-1}$ (Vais et al., 2010). *C*, the cytosolic free [Ca^{2+}], is fixed at 220 nM by the EGTA included in CLM. At the beginning of each simulation, C_{ER} is set to 500 μM (see Rossi and Taylor, 2020) and then computed at each time step for the corresponding O_4. The fraction of releasable ER Ca^{2+} was set to 80%.

To simulate experiments where the ER can refill through SERCA, we used:

$$\frac{dC_{ER}}{dt} = -(k_{leak} + k_{rel}.O_4).(C_{ER} - C) + (500 - C_{ER})/\tau_{SERCA} \quad (2)$$

with τ_{SERCA} = 100 s.

Codes for the simulations are available from https://github.com/genedupont/Quantal.

Analysis

Rates of Ca^{2+} release evoked by IP_3, ionomycin, carbachol or **2**, corrected for the basal Ca^{2+} leak (J_{leak}), were calculated by fitting a mono-exponential function to the ER Ca^{2+} content determined from the time of adding CPA alone. The derivative of this relationship at each time provides J_{leak}. From the relationship between J_{leak} and ER Ca^{2+} content (fitted to a second-degree polynomial or exponential function), J_{leak} was estimated at each time and subtracted from the observed Ca^{2+} release to identify the Ca^{2+} release evoked by IP_3, ionomycin, carbachol or **2** (Figures 1F, 1L, 3B, S3D, and S4B).

For each IP_3 or ionomycin concentration, the time evolution of ER free [Ca^{2+}] ([Ca^{2+}]$_{ER}$) was then numerically reconstructed by integration over time of the IP_3- or ionomycin-evoked Ca^{2+} flux. J_{leak} values from [Ca^{2+}]$_{ER}$ of 500 μM were also used to estimate the rate constant of Ca^{2+} leak from the ER ($k_{leak} = 1.8 \times 10^{-4}\ s^{-1}$) used in the simulations. To obtain the evolution of open receptors (Figures 7R–7T), the leak-independent Ca^{2+} flux was subtracted from the rate of Ca^{2+} release before division of each instantaneous rate by the corresponding Ca^{2+} gradient. Following Equation (1), this gives the time-evolution of the number of open receptors in arbitrary units.

To estimate the total ER Ca^{2+} content of a single HEK cell, we assume a luminal free [Ca^{2+}] of ~500 μM (Rossi and Taylor, 2020), a buffer ratio of ~5 (Prins and Michalak, 2011) (suggesting a total luminal [Ca^{2+}] of ~2.5 mM); a cell volume of 1.1×10^{12} L (http://bionumbers.hms.harvard.edu/search.aspx) of which ~10% is occupied by the nucleus and ~35% of the remaining cytoplasm is occupied by ER (~3.64×10^{-13} L) (Valm et al., 2017). Hence, we estimate the total ER Ca^{2+} content within a single HEK cell to be ~9×10^{-16} mol.

For equilibrium binding and concentration-effect relationships, results from each experiment were fitted to Hill equations (PRISM) from which pIC_{50} (-log of half-maximal inhibitory concentration) or pEC_{50} (-log of half-maximally effective concentration) values were determined. K_D^{ligand} was calculated from: $K_D^{ligand} = IC_{50}^{ligand}$ - [^{3}H-IP_3] /K_D^{IP3}. In functional assays, dose ratios (DR) were used to calculate antagonist affinities (Arunlakshana and Schild, 1959): $K_D^{antagonist}$ = [antagonist]/(DR-1). Statistical analyses of IP_3 sensitivity used pEC_{50} and pK_D values.

Statistical analyses

Experiments were performed without prior power calculations, blinding or systematic randomization. The distribution of treatments across wells in multi-well plates was varied between experiments to avoid any systematic bias arising from place-related effects. Statistical significance was assessed using Student's t test or one-way repeated ANOVA followed by Bonferroni's multiple comparisons test, with $^*p < 0.05$ considered significant (PRISM). The numbers of independent experiments and replicates for each biological experiment are indicated in figure legends. Details of all statistical tests and exact *p*values are provided in Supplemental materials and methods.

Cell Reports, Volume *37*

Supplemental information

Quantal Ca^{2+} release mediated by very few IP_3 receptors that rapidly inactivate allows graded responses to IP_3

Ana M. Rossi, Andrew M. Riley, Geneviève Dupont, Taufiq Rahman, Barry V.L. Potter, and Colin W. Taylor

SUPPLEMENTAL MATERIALS

Table S1. Properties of IP_3 and 2
Related to Figure 2.

	IP_3	**2**
**Binding**		
IP_3R1, pK_D (K_D)	7.90 ± 0.06 (12.4 nM), $n = 7$	$8.28 \pm 0.05^{***}$ (5.2 nM), $n = 8$
NT, pK_D (K_D)	8.60 ± 0.05 (2.48 nM), $n = 4$	8.63 ± 0.03 (2.32 nM), $n = 4$
IBC, pK_D (K_D)	9.62 ± 0.09 (0.24 nM), $n = 4$	$9.36 \pm 0.06^{**}$ (0.44 nM), $n = 4$
$^{a}\Delta\Delta G$	-5.2 kJ/mol	-3.8 kJ/mol
**Ca^{2+} release**		
Release	71.3 ± 0.9%, $n = 3$	$51.9 \pm 0.7\%^{***}$, $n = 3$
pEC_{50} (EC_{50})	7.38 ± 0.12 (41.6 nM), $n = 3$	$6.32 \pm 0.22^{*}$ (479 nM), $n = 3$
$^{b}EC^{I}_{50}$	41.6 nM	832 nM
pK_D-pEC^{I}_{50} (EC^{I}_{50}/K_D)	0.53 ± 0.11 (3.4), $n = 10$	$2.15 \pm 0.15^{*}$ (141), $n = 10$
**Electrophysiology**		
NP_o	0.48 ± 0.02, $n = 6$	$0.012 \pm 0.003^{****}$, $n = 6$
γ_K (pS)	219 ± 3, $n = 6$	217 ± 4, $n = 6$
τ_o (ms)	9.4 ± 0.17, $n = 6$	$4.4 \pm 0.20^{****}$, $n = 6$
τ_c (ms)	10.6 ± 1.04, $n = 6$	[c]ND

Functional responses were recorded from DT40-IP_3R1 cells. IP_3 binding assays were performed in TEM using purified cerebellar IP_3R (IP_3R1) or bacterially-expressed fragments of IP_3R1 (NT and IBC). Electrophysiology results compare the effects of 10 µM IP_3 and **2**. Results show mean ± SEM from n independent experiments. $^{*}P < 0.05$, $^{**}P < 0.01$, $^{***}P < 0.001$, $^{****}P < 0.0001$, Student's t-test, relative to IP_3. $^{a}\Delta\Delta G$ ($\Delta G^{IBC} - \Delta G^{NT}$) reports the difference in ΔG for IP_3 binding to the IBC and NT. $^{b}EC^{I}_{50}$ denotes the concentration of each ligand required to cause Ca^{2+} release equivalent to that evoked by a half-maximally effective concentration of IP_3 (the EC_{50} for IP_3). [c]ND, not determined because the very low NP_o with **2** does not allow single- and multi-channel patches to be unambiguously distinguished. It is, therefore, impossible to determine whether intervals between openings are gaps between openings of the same channel (τ_c) or between openings of different channels. τ_o, τ_c mean channel open and closed times. γ_K, single-channel K^+ conductance derived from current-voltage relationships.

Table S2. Properties of IP_3 and 2 in wild-type HEK cells
Related to Figure 2.

	IP_3	**2**
pEC_{50}	6.71 ± 0.10	6.09 ± 0.22*
Ca^{2+} release (%)	69 ± 1	51 ± 7*
h	1.0 ± 0.1	1.1 ± 0.3

Effects of IP_3 or **2** on Ca^{2+} release from permeabilized wild-type HEK cells (mean ± SEM, *n* = 3-4, each with duplicate determinations). ER Ca^{2+} content was measured 20 s after addition of IP_3 or **2**. $^{*}P < 0.05$, Student's *t*-test, relative to IP_3.

Table S3. Affinity of 2 for IP_3R determined from functional analyses
Related to Figure 2.

		IP_3-induced Ca^{2+} release		
**2** (µM)	Ca^{2+} release evoked by **2** (%)	pEC_{50} EC_{50} (nM)	Ca^{2+} release (%)	*n*
0	0	7.40 ± 0.03<br>39.4	68.0 ± 3.5	8
1	31 ± 7	6.88 ± 0.09***<br>131.8	74.3 ± 4.6	5

From the experiments shown in **Figure 2F**, we determined the Ca^{2+} release evoked by **2 (**1 µM), and then the effects of IP_3, added 30 s after **2**, on the residual Ca^{2+} stores. Results show mean ± SEM from *n* independent experiments, each with 3 replicates (mean only for EC_{50}). $^{***}P < 0.001$, Student's *t*-test. From the ratio of the two EC_{50} values for IP_3-evoked Ca^{2+} release (dose ratio, DR), the K_D for **2** was calculated: K_D = [**2**]/(DR-1) = 426 nM.

Table S4. Bioassays of IP_3 after prolonged incubation with permeabilized cells Related to Figure 5L.

	Pre-incubated	Control
pEC_{50}	6.76 ± 0.09	6.76 ± 0.09
Ca^{2+} release (%)	85 ± 8	86 ± 5
h	0.95 ± 0.1	1.6 ± 0.5

IP_3 is not metabolized after prolonged incubation with permeabilized cells. IP_3 was incubated in 96-well plates with permeabilized HEK-IP_3R1 cells (pre-incubated) or without cells (control) for 260 s under conditions identical to those used in **Figure 5G**. The supernatants from these plates (650 ×*g*, 1 min) was then added to fresh permeabilized cells loaded to steady state with Ca^{2+} to determine the Ca^{2+}-releasing activity of the extracts (**Figure 5L**). The table (mean ± SEM, $n = 4$) shows no significant differences between the pEC_{50}, maximal release (%) or Hill coefficient (*h*) values between cells stimulated with IP_3 that had been pre-incubated with cells or under control conditions

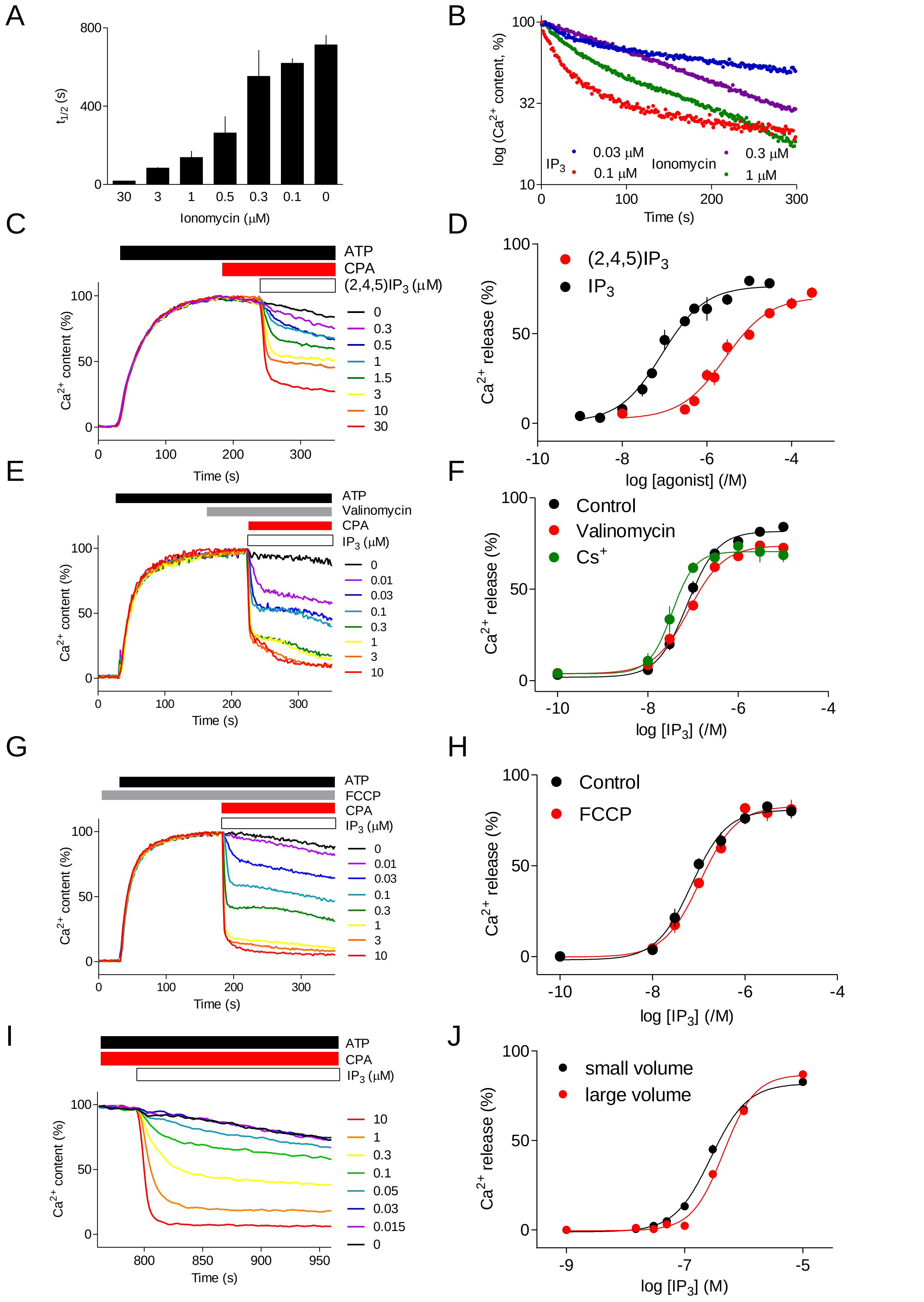

Figure S1. Quantal responses in permeabilized cells are not due to IP_3 metabolism, ineffective movement of counter-ions, contributions from mitochondria or bolus dddition of IP_3

Legend on next page

Figure S1. Quantal responses in permeabilized cells are not due to IP_3 metabolism, ineffective movement of counter-ions, contributions from mitochondria or bolus dddition of IP_3
Related to Figure 1.

Figure on preceding page

(**A**) Half-times ($t_{1/2}$) for Ca^{2+} release evoked by the indicated concentrations of ionomycin (mean ± SEM, *n* = 4) from permeabilized DT40-IP_3R1 cells (from experiments similar to those shown in **Figure 1E**).
(**B**) Semi-logarithmic plots show mono-exponential loss of ER Ca^{2+} from permeabilized DT40-IP_3R1 cells after treatment with ionomycin, and quantal release evoked by IP_3 (from data shown in **Figures 1D** and **1E**).
(**C**) Quantal Ca^{2+} release from permeabilized HEK-IP_3R1 cells evoked by (2,4,5)IP_3, a non-metabolized analogue of IP_3 (Hill et al., 1988). Results, typical of 4 experiments, show means for 4 replicates.
(**D**) Summary results show concentration-dependent effects of IP_3 and (2,4,5)IP_3 (mean ± SEM, *n* = 4, each with 3 replicates).
(**E**) Quantal responses to IP_3 in permeabilized DT40-IP_3R1 cells in the presence of valinomycin (10 µM, 1 min) to allow exchange of K^+ across ER membranes. Mean of duplicate determinations.
(**F**) Summary results (mean ± SEM, *n* = 3-4, each with duplicate determinations) show concentration-dependent quantal Ca^{2+} release by IP_3 in normal CLM, CLM with Cs^+ replacing all K^+ to inhibit K^+ channels, or CLM with valinomycin (10 µM).
(**G**) Quantal responses of permeabilized DT40-IP_3R1 cells to IP_3 in the presence of FCCP (10 µM, 10 min) to inhibit mitochondria. Results, typical of 3 or 4 experiments, show means for 2 replicates.
(**H**) Summary results show concentration-dependent effects of IP_3 alone or with FCCP (mean ± SEM, *n* = 3-4, each with duplicate determinations).
(**I**) Permeabilized HEK-G-CEPIA1*er* cells in 50 µL of CLM were loaded to steady state with Ca^{2+}; CPA (10 µM) and IP_3 (at final concentrations shown) were then added. IP_3 was added in 50 µL of CLM (at 2-times its final concentration; rather than the usual 5 µL at 10-times the final concentration). The aim was to avoid artefacts that might arise from a bolus addition of IP_3 at ten-times its final concentration exposing cells near the injection site to transiently high IP_3 concentrations. Traces from one experiment with 2 replicates are typical of 5 experiments.
(**J**) Summary results (mean ± SEM, *n* = 5, each with 2 replicates; most error bars are smaller than the symbols) show Ca^{2+} release evoked by IP_3 for assays in which the stimulus was added as a 50-µL (large volume) or 5-µL addition (small volume). ER Ca^{2+} content was measured 20 s after addition of (2,4,5)IP_3 (**D**) or IP_3 (**G, H, J**).

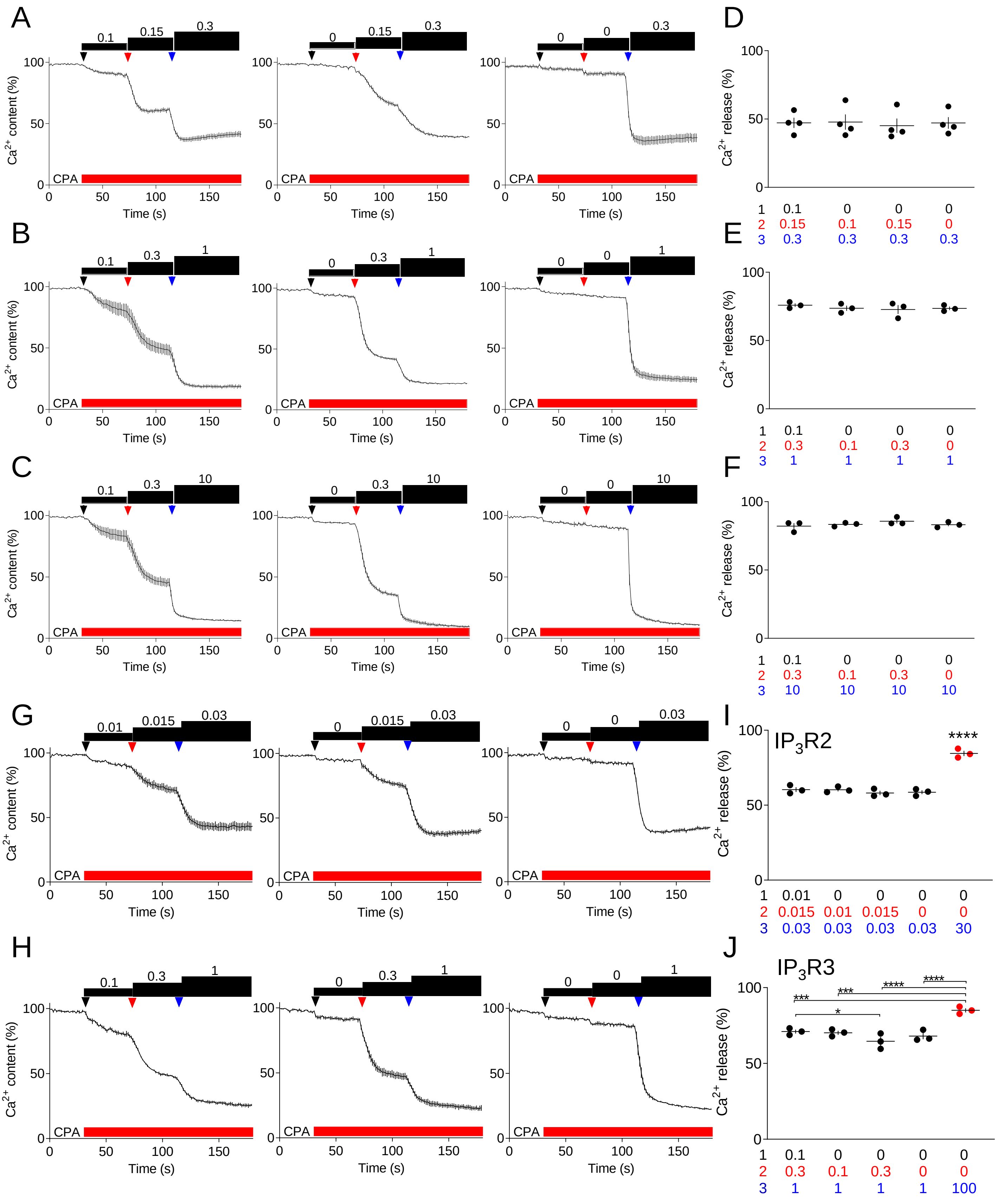

Figure S2. Incremental responses do not require an increase in $[Ca^{2+}]_c$ and occur with all IP_3R subtypes

Legend on next page

Figure S2. Incremental responses do not require an increase in $[Ca^{2+}]_c$ and occur with all IP_3R subtypes
Related to Figure 1.

Figure on preceding page

(**A-C**) In experiments similar to those shown in **Figure 1D**, permeabilized DT40-IP_3R1 cells were used to examine the effects of incremental additions of IP_3 in CLM containing 10 mM BAPTA (cumulative IP_3 concentrations shown in µM). Mean ± SD of 3 replicates.
(**D-F**) Summary results show Ca^{2+} release recorded 20 s after the final IP_3 addition for each sequence of incremental additions (individual values, mean ± SEM, *n* = 3-4, each with 3 replicates). Since BAPTA is a low-affinity competitive IP_3R antagonist (Richardson and Taylor, 1993), the sensitivity to IP_3 is reduced in the presence of 10 mM BAPTA. There were no significant differences between any of the incremental additions (**D-F**), one-way repeated ANOVA with Bonferroni's multiple comparisons test.
(**G, H**) Experiments similar to those shown in **Figures 1G-1J** show incremental responses to additions of IP_3 (cumulative IP_3 concentrations shown in µM) for permeabilized DT40 cells expressing IP_3R2 (G) or IP_3R3 (H) (mean ± SD, 3-6 replicates).
(**I, J**) Summary results (individual values, mean ± SEM, *n* = 3, each with 3-6 replicates). The responses to maximal concentrations of IP_3 (red symbols) confirm that all incremental additions evoked submaximal responses. $^{***}P < 0.001$, $^{****}P < 0.0001$, one-way repeated ANOVA with Bonferroni's test; there were no significant differences between any of the incremental additions, except where shown ($^{*}P < 0.05$). Ca^{2+} release was recorded 20 s after the final IP_3 addition for each sequence of incremental additions.

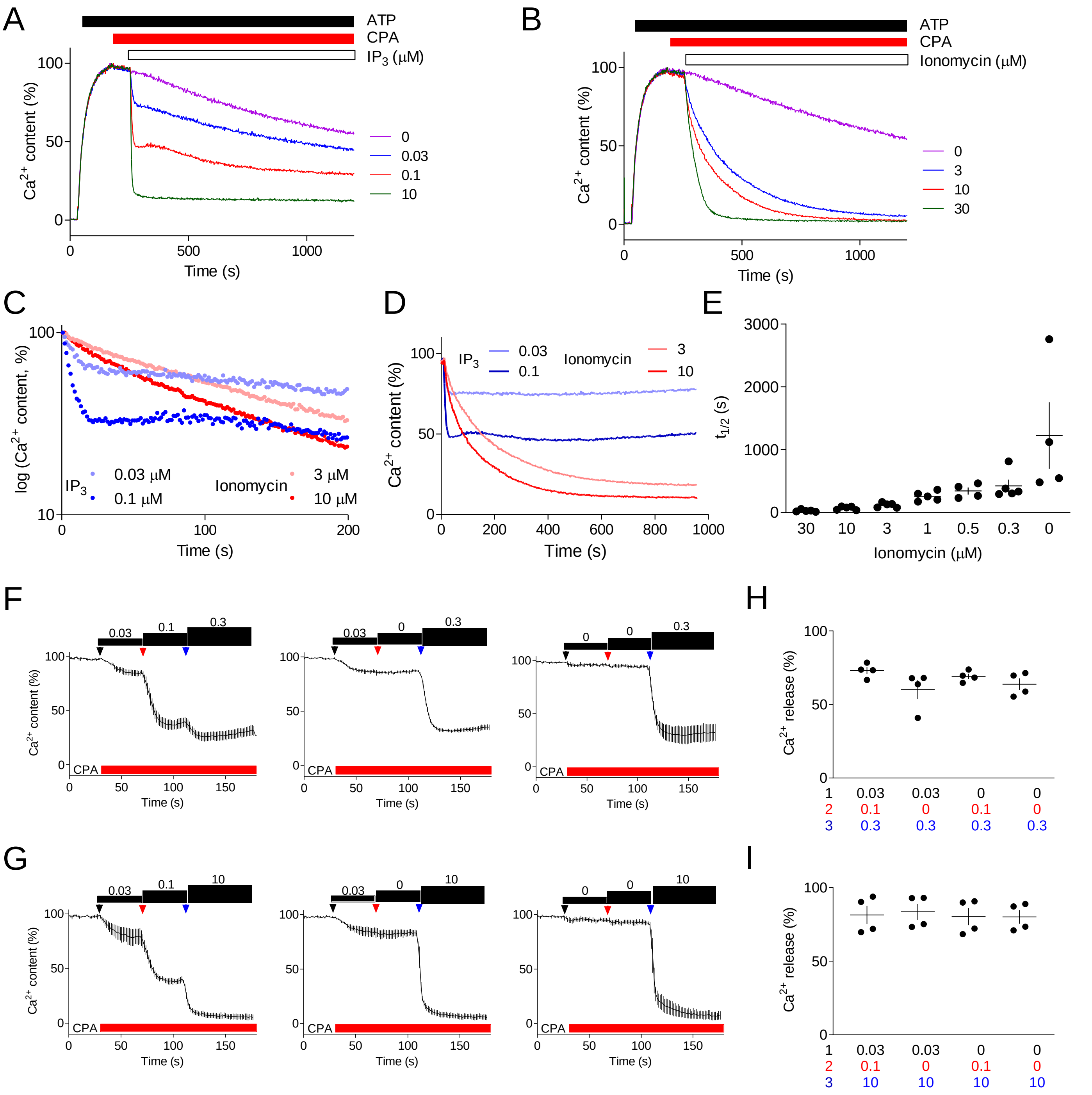

Figure S3. Incremental responses to IP_3 in permeabilized wild-type HEK cells Related to Figure 1.

(A, B) Effects of the indicated concentrations of IP_3 (**A**) or ionomycin (**B**) added to permeabilized wild-type HEK cells in which the intracellular stores had been loaded to steady state with Ca^{2+}, and with Mag-fluo 4 used to record ER luminal [Ca^{2+}]. CPA (10 µM) was added to inhibit SERCA. Results, typical of 5 independent experiments, show the mean for 2 replicates.
(**C**) Semi-logarithmic plots show the monophasic Ca^{2+} release evoked by ionomycin, and the quantal response to IP_3.
(**D**) Responses to IP_3 or ionomycin after subtraction of the basal Ca^{2+} leak.
(**E**) Summary results show half-times ($t_{1/2}$) for the loss of ER Ca^{2+} evoked by the indicated concentrations of ionomycin (individual values, mean ± SEM, *n* = 4-5, each with 2 replicates).
(**F, G**) Effects of submaximal (0.3 µM, **F**) or maximal (10 µM, **G**) concentrations of IP_3 added directly or by incremental additions (cumulative IP_3 concentrations shown in µM). Mean ± SD of 3 replicates.
(**H, I**) Summary results (individual values, mean ± SEM, *n* = 4, each with 3 replicates) show the final Ca^{2+} content of the stores (determined at 150 s) for the incremental additions (1-3, cumulative concentrations, µM). No significant differences between the incremental additions (one-way repeated ANOVA).

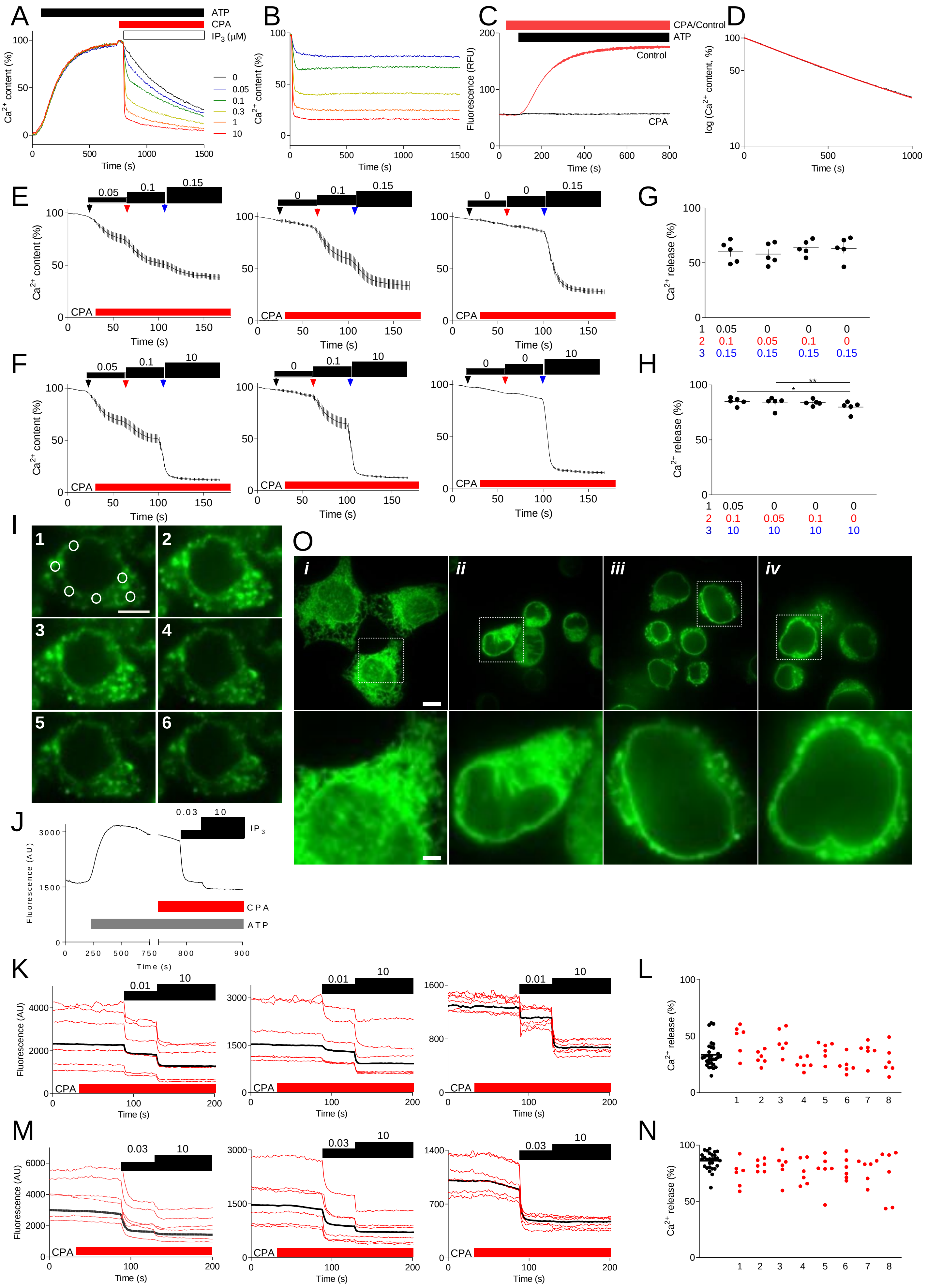

Figure S4. Quantal responses to IP_3 recorded using G-CEPIA1*er*
Legend on next page

Figure S4. Quantal responses to IP_3 recorded using G-CEPIA1*er*
Related to Figure 1.

Figure on preceding page

(**A**) Populations of HEK cells stably expressing the genetically encoded Ca^{2+} indicator, G-CEPIA1*er* (Suzuki et al., 2014) (K_D = 1.55 mM determined *in situ*) (Rossi and Taylor, 2020), were permeabilized in CLM before addition of ATP (1.5 mM), CPA (10 µM) and then IP_3 (concentrations in µM). Results show means of 3 replicates, typical of 5 experiments.
(**B**) Responses to IP_3 (from panel **A**) shown after subtraction of the basal Ca^{2+} leak.
(**C**) Addition of ATP (1.5 mM) to a population of permeabilized cells stimulates Ca^{2+} uptake into the ER, reported by the increase in G-CEPIA1*er* fluorescence. The response is abolished by CPA (10 µM) added 60 s before ATP. Results show traces (mean ± SD, each with 4 replicates) typical of 4 independent experiments. RFU, relative fluorescence units.
(**D**) Stores were loaded with Ca^{2+} before addition of CPA. The subsequent fluorescence changes recorded from the cell population are plotted semi-logarithmically. The half-time ($t_{1/2}$) is 445 ± 41 s and the intercept at time 0 (CPA addition) is 100.9 ± 0.4 % (mean ± SEM, *n* = 7, each with 12-16 replicates). The latter confirms the linear relationship between ER free [Ca^{2+}] and fluorescence across the entire range of ER Ca^{2+} contents examined, suggesting that in replete stores the free [Ca^{2+}] is less than the K_D of the indicator (1.55 mM). Similar estimates for the rate of Ca^{2+} leak determined using Mag-fluo 4 ($t_{1/2}$ = 356 ± 52 s) (Rossi and Taylor, 2020) and G-CEPIA1*er* ($t_{1/2}$ = 445 ± 41 s) confirm the reliability of measurements with Mag-fluo 4 and G-CEPIA1*er*.
(**E, F**) Incremental responses from populations of permeabilized cells to the indicated concentrations of IP_3 added after CPA (10 µM), recorded using G-CEPIA1*er* (cumulative IP_3 concentrations shown in µM).
(**G, H**) Summary results (individual values, mean ± SEM, *n* = 5, each with 3 replicates) show final Ca^{2+} content of the stores (determined at 150 s) for the incremental additions (cumulative IP_3 concentrations shown in µM). No significant differences, except where indicated ($^{*}P < 0.05$, $^{**}P < 0.01$, one-way repeated ANOVA with Bonferroni's test).
The results (**E-H**) demonstrate that incremental responses to IP_3 are not an artefact arising from use of luminal Ca^{2+} indicators failing to reliably report luminal [Ca^{2+}] (see also Rossi and Taylor, 2020).
(**I**) HEK-G-CEPIA1*er* cells were permeabilized before addition of ATP, then CPA (10 µM) to inhibit SERCA, and IP_3. Confocal sections show a single permeabilized cell before (1) and 700 s after addition of ATP (2), immediately before (3) and 20 s after adding 30 nM IP_3 (4), and before (5) and 20 s after (6) addition of 10 µM IP_3. Scale bar = 5 µm. Circles show typical subcellular regions of interest (ROI, 1.5-µm diameter) selected for the analyses shown in panels **K-N**.
(**J**) Time-course of the fluorescence changes recorded from an entire cell.
(**K**) Three examples of whole-cell responses (black lines) and ROI within each cell (red lines) to addition of CPA and then sequential stimulation with 10 nM and then 10 µM IP_3. AU, arbitrary units.
(**L**) Summary results (individual values, mean ± SD, *n* = 31 cells) show responses to 10 nM IP_3 measured 20 s after its addition (% of IP_3-sensitive Ca^{2+} stores) in whole cells (black) and 6 randomly selected ROIs (red) from within 8 cells (1-8).
(**M**) Examples of responses from entire cells (black lines) and ROI within each cell (red lines) to addition of CPA and then sequential stimulation with 30 nM and then 10 µM IP_3. In most cells (28/33), 30 nM IP_3 released only a fraction of the IP_3-sensitive stores (first two examples), but in a few cells (5/33) it evoked a maximal response (last example).
(**N**) Summary results (individual values, mean ± SD, *n* = 28 cells) show responses to 30 nM IP_3 measured 20 s after its addition in whole cells (black) and 6 randomly selected ROIs (red) within 8 cells (1-8) in which 30 nM IP_3 evoked a submaximal response (% of IP_3-sensitive Ca^{2+} stores).
These results (**K-N**) establish that quantal responses to IP_3 occur within single cells and they show that there is no evident heterogeneity in IP_3 sensitivity between ROIs within single cells.
(**O**) Confocal images of intact HEK-G-CEPIA*er* cells grown on poly-L-lysine-coated dishes ***(i)***, and HEK-G-CEPIA*er* cells after trypsinization and attachment to poly-L-lysine-coated dishes ***(ii);*** HEK-G-CEPIA*er* cells ***(iii)*** and HEK cells loaded with Mag-fluo 4 (***iv***) after permeabilization and attachment to poly-L-lysine-coated dishes. Scale bar = 10 µm (2.5 µm in enlargements). Results are typical of 3 independent experiments (~150 cells in each). The results show that there is partial fragmentation of the ER after permeabilization, which is similar for both Ca^{2+} indicators.

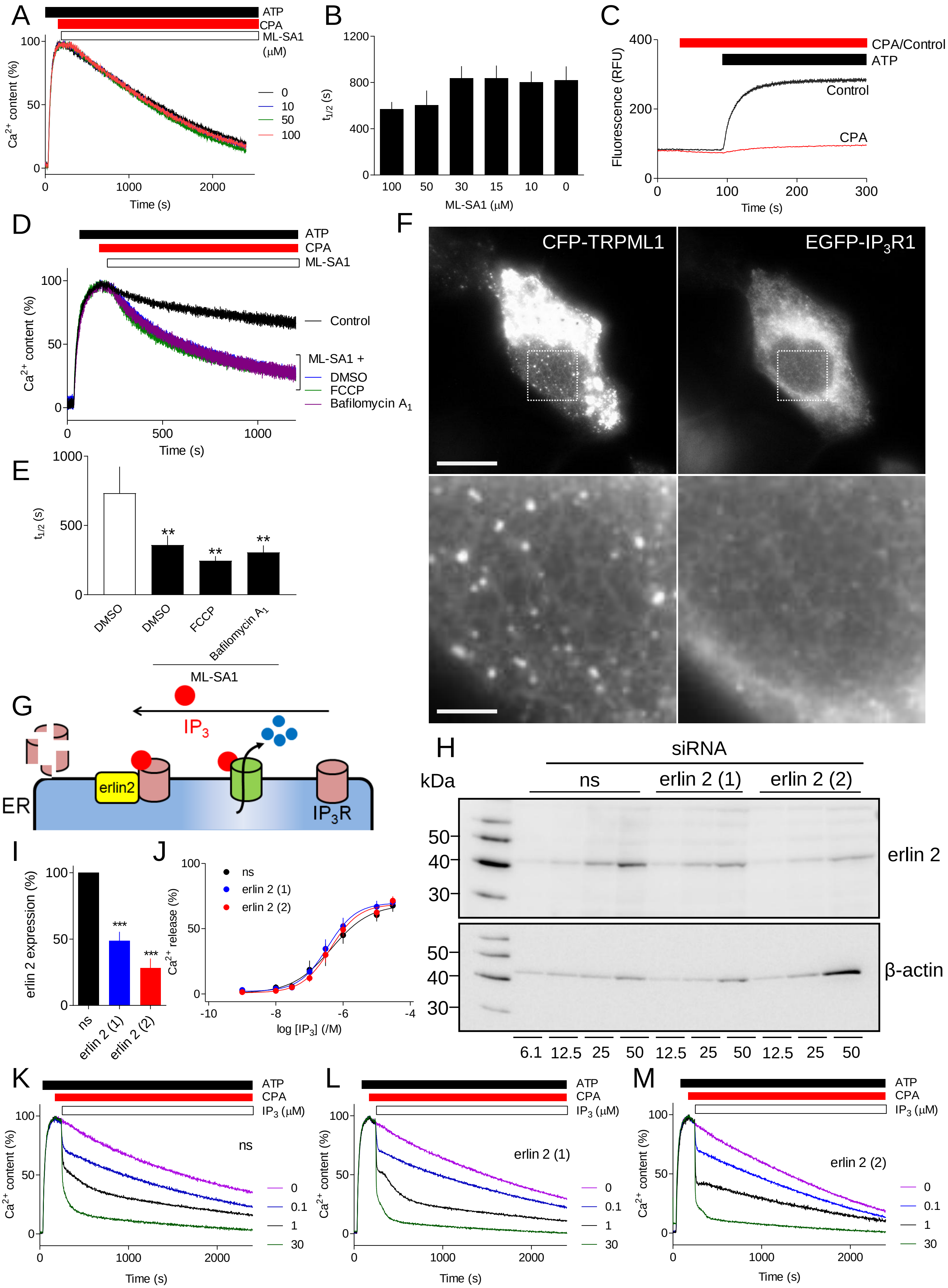

Figure S5. Evidence that over-expressed TRPML1 releases Ca^{2+} from ER, and that erlin 2 does not mediate quantal Ca^{2+} release

Legend on next page

Figure S5. Evidence that over-expressed TRPML1 releases Ca^{2+} from ER, and that erlin 2 does not mediate quantal Ca^{2+} release
Related to Figure 4.

Figure on preceding page

(**A**) Experiments conducted in parallel with those shown in **Figure 4A** demonstrate that mock-transfected HEK cells do not respond to ML-SA1. Ca^{2+} uptake into the ER of permeabilized HEK cells was initiated by addition of ATP (1.5 mM), before adding CPA (10 µM) and then the indicated concentration of ML-SA1 (0-100 µM). Results show mean ± SD of 3 replicates, typical of 4 independent experiments.
(**B**) Summary results from experiments similar to those shown in **Figure 4A** show half-times ($t_{1/2}$) for loss of ER Ca^{2+} evoked by the indicated concentrations of ML-SA1 (mean ± SEM, n = 4, each with 2 replicates).
(**C**) Addition of ATP (1.5 mM) to permeabilized HEK cells stimulates Ca^{2+} uptake into the ER, reported by the increase in Mag-fluo 4 fluorescence. The response is abolished by CPA (10 µM) added 60 s before ATP. Mean ± SEM, n = 4, each with 2 replicates. RFU, relative fluorescence units. These results demonstrate that Mag-fluo 4 selectively reports ER luminal [Ca^{2+}].
(**D**) Permeabilized HEK cells expressing CFP-TRPML1 were treated (60 min) with vehicle (DMSO), bafilomycin A_1 (1 µM to inhibit the lysosomal H^+ pump) or FCCP (10 µM to dissipate H^+ gradients) before addition of ATP (1.5 mM) to initiate Ca^{2+} uptake. After addition of CPA (10 µM), ML-SA1 (30 µM) was added where indicated. Mean ± SD of 3 replicates.
(**E**) Summary results (mean ± SEM, n = 4-5, each with 3-6 replicates) show half-times ($t_{1/2}$) for the mono-exponential rates of Ca^{2+} release after each treatment. $^{**}P < 0.01$, one-way ANOVA with Bonferroni's test, relative to DMSO with no ML-SA1. These results demonstrate that TRPML1, which is natively expressed in lysosomes, releases Ca^{2+} from the ER when it is over-expressed. We note that our assay further ensures that we measure only ER Ca^{2+} release because Mag-fluo 4 selectively reports Ca^{2+} uptake into the ER (Rossi and Taylor, 2020).
(**F**) TIRFM images show expression of CFP-TRPML1 and EGFP-IP_3R1 in HEK cells expressing both proteins, typical of 3 independent experiments (~10 cells in each). Scale bar = 20 µm (4 µm for enlargement). As expected, CFP-TRPML1 is expressed most in lysosomes, but there is an underlying reticular distribution that is similar to that of EGFP-IP_3R1. It was impracticable, when most CFP-TRPML1 is lysosomal, to quantify any colocalization with IP_3Rs. We note that our use of Ca^{2+} indicators that exclusively report ER luminal [Ca^{2+}] ensure that in functional assays, we detect only the behaviour of TRPML1 expressed in the ER.
(**G**) IP_3 binding promotes both opening of the IP_3R channel and association of IP_3R with erlin 2, which targets the IP_3R for ubiquitination and degradation (Wojcikiewicz, 2018). We considered whether rapid association of active IP_3Rs with erlin 2 might inactivate them before their degradation.
(**H**) Western blot using an antibody against erlin 2 or β-actin (loading control) of lysates prepared from HEK cells treated with a non-silencing siRNA (ns) or either of two different siRNAs against erlin 2. Protein loadings (µg) are shown beneath each lane, and molecular weight markers are shown on the left. Since the molecular weight of erlin 2 is similar to that of β-actin (and most other loading controls), two identical gels were run, one blotted with erlin 2 and the other with β-actin.
(**I**) Summary results (mean ± SEM, n = 4) show erlin 2 expression relative to cells treated with ns siRNA. Knock-down efficiency of siRNA erlin 2 (1) and (2) was 51 ± 7% and 72 ± 7%, respectively. $^{***}P < 0.001$, one-way ANOVA with Bonferroni's test.
(**J**) Methods similar to those in **Figure 1D** were used to determine the effects of IP_3 on Ca^{2+} release from the intracellular stores of permeabilized HEK cells after treatment with the indicated siRNAs. ER Ca^{2+} content was measured 20 s after addition of IP_3. Mean ± SEM, n = 4, each with 3 replicates.
(**K-M**) Ca^{2+} content of the ER of permeabilized HEK cells is shown after addition of ATP (1.5 mM), followed by CPA (10 µM) and then the indicated concentrations of IP_3 (µM) in cells treated with ns siRNA (**K**) or siRNAs directed to erlin 2 (**L, M**). Results (mean of 3 replicates) show Ca^{2+} contents relative to steady-state Ca^{2+} content (%). Similar results were obtained in 4 independent experiments.

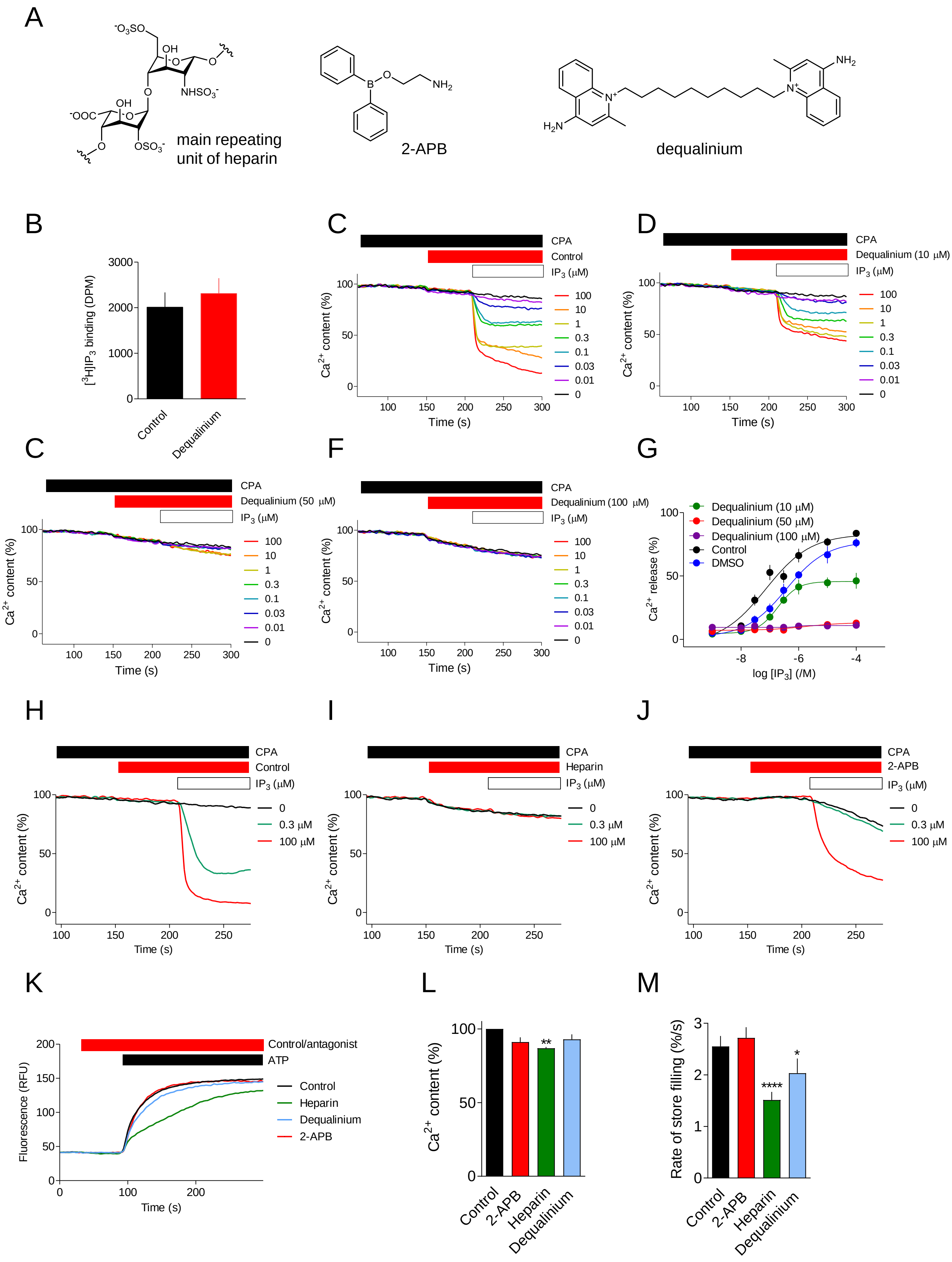

Figure S6. Effects of heparin, 2-APB and dequalinium on IP_3Rs and Ca^{2+} uptake by intracellular stores

Legend on next page

Figure S6. Effects of heparin, 2-APB and dequalinium on IP_3Rs and Ca^{2+} uptake by intracellular stores
Related to Figures 5 and 6.

Figure on preceding page

(**A**) Structures of the antagonists used.
(**B**) Effects of dequalinium (50 µM) on specific $[^3H]IP_3$ (7.5 nM) binding to cerebellar membranes in CLM at 20ºC. Mean ± SEM, $n = 3$. Heparin is a competitive antagonist of IP_3, while 2-APB (like dequalinium) does not affect IP_3 binding (Saleem et al., 2014). No significant difference, Student's *t*-test.
(**C-F**) Permeabilized HEK-IP_3R1 cells were loaded with Ca^{2+}, and SERCA was then inhibited with CPA (10 µM) before addition of DMSO (1% v/v; **C**) or dequalinium (10-100 µM, **D-F**), and then the indicated concentrations of IP_3. Mean of 2 replicates, typical of 5 experiments.
(**G**) Summary results (mean ± SEM, $n = 5$, each with duplicate determinations) show Ca^{2+} released by IP_3 in the presence of the indicated concentrations of dequalinium. ER Ca^{2+} content was measured 20 s after IP_3 addition.
(**H-J**) Similar analyses of the effects of heparin (10 mg/mL, **I**) or 2-APB (125 µM, **J**). Means of 2 replicates, typical of 3-5 experiments.
(**K**) Effects of dequalinium (50 µM), heparin (10 mg/mL) or 2-APB (125 µM) on Ca^{2+} uptake by the intracellular stores of permeabilized HEK-IP_3R1 cells. Mean ± SD of 4-6 replicates, typical of 4 experiments.
(**L, M**) Summary results show the effects of antagonists on the steady-state Ca^{2+} content of the stores (**L**) and the rate of Ca^{2+} uptake (**M**). ER Ca^{2+} content was measured 180 s after addition of ATP (**L**). Mean ± SEM, $n = 4$, each with 4-6 determinations. $^{****}P < 0.0001$, $^{***}P < 0.001$, $^{*}P < 0.05$ one-way ANOVA with Bonferroni's test relative to control.

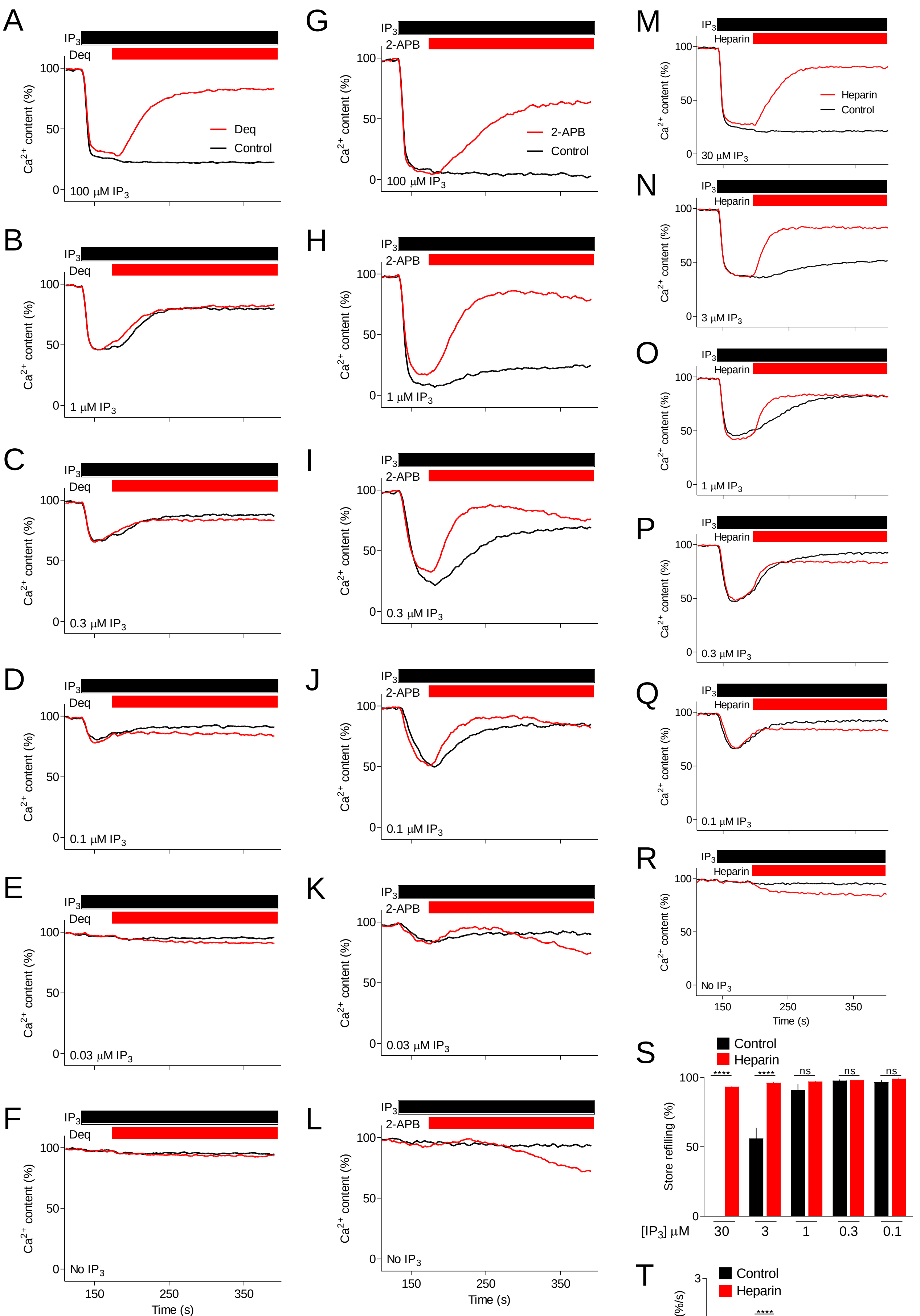

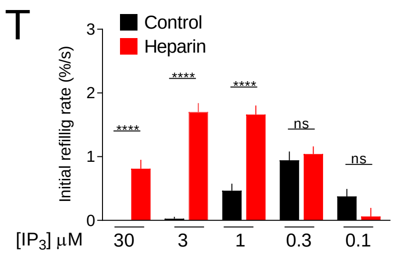

Figure S7. Blocking IP_3Rs with antagonists reveals that IP_3Rs close after incremental responses to low IP_3 concentrations

Legend on next page

Figure S7. Blocking IP_3Rs with antagonists reveals that IP_3Rs close after incremental responses to low IP_3 concentrations
Related to Figures 5 and 6.

Figure on preceding page

(**A-L**) Experiments similar to those shown in **Figure 5** were used to assess whether IP_3Rs remain open during sustained stimulation with the indicated concentrations of IP_3 using dequalinium (50 µM, **A-F**) or 2-APB (125 µM, **G-L**) as the IP_3R antagonist. Traces show means of 2 replicates. Summary results are shown in **Figures 5J** and **5K**.
(**M-R**) Experiments similar to those in **Figure 5** were repeated using wild-type HEK cells. Permeabilized cells were loaded with Ca^{2+} by addition of ATP before addition of the indicated concentrations of IP_3 (without inhibiting SERCA) and then the IP_3R antagonist, heparin (10 mg/mL). Traces show means of 2 replicates.
(**S, T**) Summary (mean ± SEM, $n = 3$, each with 2 determinations) show the extent to which stores refill (relative to cells without IP_3) 250 s after IP_3 addition (**S,** 0% refilling is defined as the content measured 250 s after addition of 30 µM IP_3 alone), and the initial rate of refilling from the slope of the curve immediately after heparin addition (**T**). $^{****}P < 0.0001$, one-way ANOVA with Bonferroni's test.

Methods S 1. Summary of Statistical Analyses. Related to STAR Methods.
The table summarises the results of all statistical analyses reported. $^{*}P < 0.05$, $^{**}P < 0.01$, $^{***}P < 0.001$, $^{****}P < 0.0001$.

**Figure**	**Test**	***P* values and summary**			
**Figure 1I**	Repeated measures one-way ANOVA.	$P = 0.2594$ (ns), ANOVA provides no justification for Bonferroni’s test.			
**Figure 1J**	Repeated measures one-way ANOVA	$P = 0.2021$ (ns), ANOVA provides no justification for Bonferroni’s test.			
**Figure 1M**	Ordinary one-way ANOVA with Bonferroni’s multiple comparisons test	$P < 0.0001$			
		Bonferroni’s test for comparisons of Ca^{2+} release recorded at 400 s versus 700 s for each of the four concentrations of CCh.			
			Adjusted *P*	$P < 0.05$	Summary
		1000µM CCh, 400 s vs 700 s	>0.9999	No	ns
		3µM CCh, 400 s vs 700 s	>0.9999	No	ns
		1.5µM CCh, 400 s vs 700 s	>0.9999	No	ns
		1µM CCh, 400 s vs 700 s	>0.9999	No	ns
**Figure 1O**	Repeated measures one-way ANOVA with Bonferroni’s multiple comparisons test	$P < 0.0001$			
		Bonferroni's test for all comparisons			
			Adjusted *P*	$P < 0.05$	Summary
		1µM /3µM vs 0µM /3µM	0.1925	No	ns
		1µM /3µM vs 1µM /1000µM	0.0009	Yes	***
		1µM /3µM vs 3µM /1000µM	0.0011	Yes	**
		0µM /3µM vs 1µM /1000µM	0.0002	Yes	***
		0µM /3µM vs 3µM /1000µM	0.0002	Yes	***
		1µM /1000µM vs 3µM /1000µM	>0.9999	No	ns
**Figure 2E**	Ordinary one-way ANOVA with Bonferroni's multiple comparisons test	$P = 0.0305$			
		Bonferroni's test for all comparisons			
			Adjusted *P*	$P < 0.05$	Summary
		10µM IP_3 vs 10µM **2**	<0.0001	Yes	****
		10µM IP_3 vs 100µM **2**	<0.0001	Yes	****
		10µM IP_3 vs 10µM IP_3 with10µM **2**	<0.0001	Yes	****
		10µM **2** vs 100µM **2**	>0.9999	No	ns
		10µM **2** vs 10µM IP_3 with10µM **2**	0.0170	Yes	*
		100µM **2** vs 10µM IP_3 with10µM **2**	0.0111	Yes	*

**Figure 3D**	Repeated measures one-way ANOVA with Bonferroni's multiple comparisons test	$P < 0.0001$<br>Bonferroni's test for all comparisons			
			Adjusted *P*	$P < 0.05$	Summary
		0.1/0.3/1 vs 0/0.1/1	0.2683	No	ns
		0.1/0.3/1 vs 0/0.3/1	2.353	No	ns
		0.1/0.3/1 vs 0/0/1	-1.392	No	ns
		0.1/0.3/1 vs 0/0/100	-21.07	Yes	***
		0/0.1/1 vs 0/0.3/1	2.084	No	ns
		0/0.1/1 vs 0/0/1	-1.660	No	ns
		0/0.1/1 vs 0/0/100	-21.34	Yes	***
		0/0.3/1 vs 0/0/1	-3.745	No	ns
		0/0.3/1 vs 0/0/100	-23.42	Yes	***
		0/0/1 vs 0/0/100	-19.68	Yes	***

Figure	Test	Comparison	Adjusted P	$P < 0.05$	Summary
**Figure 3F**	Repeated measures one-way ANOVA with Bonferroni's multiple comparisons test	$P < 0.0001$<br>Bonferroni's test for all comparisons			
		0µM **2**/ 100µM IP_3 vs 0µM **2**/ 300µM IP_3	>0.9999	No	ns
		0µM **2**/ 100µM IP_3 vs 30µM **2**/ 0µM IP_3	<0.0001	Yes	****
		0µM **2**/ 100µM IP_3 vs 100µM **2**	0.0002	Yes	***
		0µM **2**/ 100µM IP_3 vs 30µM **2**/ 100µM IP_3	>0.9999	No	ns
		0µM **2**/ 100µM IP_3 vs 30µM **2**/ 300µM IP_3	0.3147	No	ns
		0µM **2**/ 300µM IP_3 vs 30µM **2**/ 0µM IP_3	<0.0001	Yes	****
		0µM **2**/ 300µM IP_3 vs 100µM **2**	0.0007	Yes	***
		0µM **2**/ 300µM IP_3 vs 30µM **2**/ 100µM IP_3	>0.9999	No	ns
		0µM **2**/ 300µM IP_3 vs 30µM **2**/ 300µM IP_3	>0.9999	No	ns
		30µM **2**/ 0µM IP_3 vs 100µM **2**	>0.9999	No	ns
		30µM **2**/ 0µM IP_3 vs 30µM **2**/ 100µM IP_3	0.0003	Yes	***
		30µM **2** / 0µM IP_3 vs 30µM **2**/ 300µM IP_3	0.0034	Yes	**
		100µM **2** vs 30µM **2**/ 100µM IP_3	0.0044	Yes	**
		100µM **2** vs 30µM **2**/ 300µM IP_3	0.0513	No	ns
		30µM **2**/ 100µM IP_3 vs 30µM **2**/ 300µM IP_3	>0.9999	No	ns
**Figure 5H**	Repeated measures one-way ANOVA with Bonferroni's multiple comparisons test	$P < 0.0001$<br>Bonferroni's test for only the indicated comparisons			
		100 control vs 100 with heparin	<0.0001	Yes	****
		10 control vs 10 with heparin	<0.0001	Yes	****
		1 control vs 1with heparin	<0.0001	Yes	****
		0.3 control vs 0.3 with heparin	<0.0001	Yes	****
		0.1 control vs 0.1 with heparin	0.4821	No	ns
		0.03 control vs 0.03 with heparin	>0.9999	No	ns
**Figure 5I**	Repeated measures one-way ANOVA with Bonferroni's multiple comparisons test	$P < 0.0001$<br>Bonferroni's test for only the indicated comparisons			
		100 control vs 100 with heparin	<0.0001	Yes	****
		10 control vs 10 with heparin	<0.0001	Yes	****
		1 control vs 1with heparin	<0.0001	Yes	****
		0.3 control vs 0.3 with heparin	<0.0001	Yes	****
		0.1 control vs 0.1 with heparin	>0.9999	No	ns
		0.03 control vs 0.03 with heparin	>0.9999	No	ns

**Figure 5J**	Repeated measures one-way ANOVA with Bonferroni's multiple comparisons test	*P* < 0.0001<br>Bonferroni's test for only the indicated comparisons<br>Adjusted *P* / *P* < 0.05 / Summary<br>100 Control vs 100 with dequalinium / <0.0001 / Yes / ****<br>10 Control vs 10 with dequalinium / <0.0001 / Yes / ****<br>1 Control vs 1 with dequalinium / 0.0003 / Yes / ***<br>0.3 Control vs 0.3 with dequalinium / 0.9947 / No / ns<br>0.1 Control vs 0.1 with dequalinium / 0.9827 / No / ns<br>0.03 Control vs 0.03 with dequalinium / >0.9999 / No / ns
**Figure 5K**	Repeated measures one-way ANOVA with Bonferroni's multiple comparisons test	*P* < 0.0001<br>Bonferroni's test for only the indicated comparisons<br>Adjusted *P* / *P* < 0.05 / Summary<br>100 Control vs 100 with 2-APB / <0.0001 / Yes / ****<br>10 Control vs 10 with 2-APB / <0.0001 / Yes / ****<br>1 Control vs 1 with 2-APB / <0.0001 / Yes / ****<br>0.3 Control vs 0.3 with 2-APB / 0.0151 / Yes / *<br>0.1 Control vs 0.1 with 2-APB / 0.5176 / No / ns<br>0.03 Control vs 0.03 with 2-APB / 0.9995 / No / ns
**Figure 6G**	One-way ANOVA with Bonferroni's multiple comparisons test	*P* < 0.0001<br>Bonferroni's test for comparisons between control and dequalinium (DQ) for each concentration (µM) of (2,4,5)IP_3<br>Adjusted *P* / P < 0.05 / Summary<br>10 Control vs 10 with DQ / <0.0001 / Yes / ****<br>3Control vs 3 with DQ / <0.0001 / Yes / ****<br>1.5 Control vs 1.5 with DQ / 0.4673 / No / ns<br>1 Control vs 1 with DQ / >0.9999 / No / ns<br>0.5 Control vs 0.5 with DQ / 0.9815 / No / ns
**Figure 6H**	One-way ANOVA with Bonferroni's multiple comparisons test	*P* < 0.0001<br>Bonferroni's test for comparisons between control and dequalinium (DQ) for each concentration (µM) of (2,4,5)IP_3<br>Adjusted *P* / P < 0.05 / Summary<br>10 Control vs 10 with DQ / <0.0001 / Yes / ****<br>3Control vs 3 with DQ / <0.0001 / Yes / ****<br>1.5 Control vs 1.5 with DQ / 0.0146 / Yes / *<br>1 Control vs 1 with DQ / 0.9766 / No / ns<br>0.5 Control vs 0.5 with DQ / >0.9999 / No / ns

**Figure S2D**	Repeated measures one-way ANOVA.	*P* = 0.7935 (ns), ANOVA provides no justification for Bonferroni's test.
**Figure S2E**	Repeated measures one-way ANOVA.	*P* = 0.5315 (ns), ANOVA provides no justification for Bonferroni's test.
**Figure S2F**	Repeated measures one-way ANOVA.	*P* = 0.2898 (ns), ANOVA provides no justification for Bonferroni's test.
**Figure S2I**	Repeated measures one-way ANOVA with Bonferroni's multiple comparisons test	*P* < 0.0001<br>Bonferroni's test for all comparisons
**Figure S2J**	Repeated measures one-way ANOVA with Bonferroni's multiple comparisons test	*P* < 0.0001<br>Bonferroni's test for all comparisons

Figure S2I:

	Adjusted *P*	*P* < 0.05	Summary
0.01/0.015/0.03 vs 0/0.01/0.03	>0.9999	No	ns
0.01/0.015/0.03 vs 0/0.015/0.03	>0.9999	No	ns
0.01/0.015/0.03 vs 0/0/0.03	>0.9999	No	ns
0.01/0.015/0.03 vs 0/0/30	<0.0001	Yes	****
0/0.01/0.03 vs 0/0.015/0.03	>0.9999	No	ns
0/0.01/0.03 vs 0/0/0.03	>0.9999	No	ns
0/0.01/0.03 vs 0/0/30	<0.0001	Yes	****
0/0.015/0.03 vs 0/0/0.03	>0.9999	No	ns
0/0.015/0.03 vs 0/0/30	<0.0001	Yes	****
0/0/0.03 vs 0/0/30	<0.0001	Yes	****

Figure S2J:

	Adjusted *P*	*P* < 0.05	Summary
0.1/0.3/1 vs 0/0.1/1	>0.9999	No	ns
0.1/0.3/1 vs 0/0.3/1	0.0374	Yes	*
0.1/0.3/1 vs 0/0/1	0.9790	No	ns
0.1/0.3/1 vs 0/0/100	0.0002	Yes	***
0/0.1/1 vs 0/0.3/1	0.0764	No	ns
0/0.1/1 vs 0/0/1	>0.9999	No	ns
0/0.1/1 vs 0/0/100	0.0001	Yes	***
0/0.3/1 vs 0/0/1	0.6215	No	ns
0/0.3/1 vs 0/0/100	<0.0001	Yes	****
0/0/1 vs 0/0/100	<0.0001	Yes	****

**Figure S3H**	Repeated measures one-way ANOVA with Bonferroni's multiple comparisons test	*P* = 0.0416<br>Bonferroni’s test for all comparisons<br>Adjusted *P* / *P* < 0.05 / Summary<br>0.03/0.1/0.3 vs 0.03/0/0.3 — 0.0578 — No — ns<br>0.03/0.1/0.3 vs 0/0.1/0.3 — >0.9999 — No — ns<br>0.03/0.1/0.3 vs 0/0/0.3 — 0.2652 — No — ns<br>0.03/0/0.3 vs 0/0.1/0.3 — 0.2946 — No — ns<br>0.03/0/0.3 vs 0/0/0.3 — >0.9999 — No — ns<br>0/0.1/0.3 vs 0/0/0.3 — >0.9999 — No — ns
**Figure S3I**	Repeated measures one-way ANOVA with Bonferroni's multiple comparisons test	*P* = 0.0468<br>Bonferroni’s test for all comparisons<br>Adjusted *P* / *P* < 0.05 / Summary<br>0.03/0.1/10 vs 0.03/0/10 — 0.5500 — No — ns<br>0.03/0.1/10 vs 0/0.1/10 — >0.9999 — No — ns<br>0.03/0.1/10 vs 0/0/10 — >0.9999 — No — ns<br>0.03/0/10 vs 0/0.1/10 — 0.1050 — No — ns<br>0.03/0/10 vs 0/0/10 — 0.0809 — No — ns<br>0/0.1/10 vs 0/0/10 — >0.9999 — No — ns
**Figure S4G**	Repeated measures one-way ANOVA	*P* = 0.4676 (ns), ANOVA provides no justification for Bonferroni’s test.
**Figure S4H**	One-way repeated ANOVA with Bonferroni's multiple comparisons test	*P* = 0.0305<br>Bonferroni's test for all comparisons<br>Adjusted *P* / *P* < 0.05 / Summary<br>0.05/0.1/10 vs 0/0.05/10 — >0.9999 — No — ns<br>0.05/0.1/10 vs 0/0.1/10 — 0.7900 — No — ns<br>0.05/0.1/10 vs 0/0/10 — 0.0450 — Yes — *<br>0/0.05/10 vs 0/0.1/10 — >0.9999 — No — ns<br>0/0.05/10 vs 0/0/10 — 0.0051 — Yes — **<br>0/0.1/10 vs 0/0/10 — 0.2752 — No — ns
**Figure S5E**	One-way ANOVA with Bonferroni's multiple comparisons test	*P* = 0.0027<br>Bonferroni's test for comparisons with DMSO control<br>Adjusted *P* / *P* < 0.05 / Summary<br>Control vs ML-SA1 with FCCP — 0.0016 — Yes — **<br>Contro vs ML-SA1 with Bafilomycin A_1 — 0.0041 — Yes — **<br>Control vs ML-SA1 with DMSO — 0.0099 — Yes — **

**Figure S5I**	Repeated measures one-way ANOVA with Bonferroni's multiple comparisons test	*P* < 0.0001<br>Bonferroni's test for all comparisons<br>Adjusted *P* / *P* < 0.05 / Summary<br>ns vs erlin 2 (1): 0.0007 / Yes / ***<br>ns vs erlin 2 (2): <0.0001 / Yes / ***<br>erlin 2 (1) vs erlin 2 (2): 0.0594 / No / ns
**Figure S6B**	Paired, two-tailed Student's *t*-test	*P* = 0.1411 (ns)
**Figure S6L**	Repeated measures one-way ANOVA with Bonferroni's multiple comparisons test	*P* = 0.0132<br>Bonferroni's test for comparisons relative to control<br>Adjusted *P* / *P* < 0.05 / Summary<br>Control vs 2-APB: 0.0561 / No / ns<br>Control vs heparin: 0.0056 / Yes / **<br>Control vs dequalinium: 0.1510 / No / ns
**Figure S6M**	Repeated measures one-way ANOVA with Bonferroni's multiple comparisons test	*P* < 0.0001<br>Bonferroni's test for comparisons relative to control<br>Adjusted *P* / *P* < 0.05 / Summary<br>Control vs 2-APB: 0.9086 / No / ns<br>Control vs heparin: <0.0001 / Yes / ****<br>Control vs dequalinium: <0.0163 / Yes / *
**Figure S7S**	Repeated measures one-way ANOVA with Bonferroni's multiple comparisons test	*P* < 0.0001<br>Bonferroni's test for only the indicated comparisons<br>Adjusted *P* / *P* < 0.05 / Summary<br>30 Control vs 30 with heparin: <0.0001 / Yes / ****<br>3 Control vs 3 with heparin: <0.0001 / Yes / ****<br>1 Control vs 1 with heparin: 0.4804 / No / ns<br>0.3 Control vs 0.3 with heparin: >0.9999 / No / ns<br>0.1 Control vs 0.1 with heparin: 0.9753 / No / ns
**Figure S7T**	Repeated measures one-way ANOVA with Bonferroni's multiple comparisons test	*P* < 0.0001<br>Bonferroni's test for only the indicated comparisons<br>Adjusted *P* / P < 0.05 / Summary<br>30 Control vs 30 with heparin: <0.0001 / Yes / ****<br>3 Control vs 3 with heparin: <0.0001 / Yes / ****<br>1 Control vs 1 with heparin: <0.0001 / Yes / ****<br>0.3 Control vs 0.3 with heparin: 0.9733 / No / ns<br>0.1 Control vs 0.1 with heparin: 0.1990 / No / ns

**Table S1**	Unpaired, 2-tailed Student's *t*-test.	All comparisons are for IP_3 vs **2**<br>Binding (pK_D)<br>IP_3R1: $P = 0.0002$ (***)<br>NT: $P = 0.2873$ (ns)<br>IBC: $P = 0.0023$ (**)<br>Ca^{2+} release<br>Maximal release: $P < 0.001$ (***)<br>pEC_{50}: $P = 0.0120$ (*)<br>Electrophysiology<br>NP_o: $P < 0.0001$ (****)<br>γ_K: $P = 0.7485$ (ns)<br>τ_o: $P < 0.0001$ (****)
**Table S2**	Unpaired, two-tailed Student's *t*-test	pEC_{50}: $P = 0.0354$ (*)<br>Ca^{2+} release: $P = 0.0279$ (*)<br>h: $P = 0.4855$ (ns)
**Table S3**	Unpaired, 2-tailed Student's *t*-test.	Comparisons of Ca^{2+} release evoked by IP_3 alone or after incubation with **2**<br>pEC_{50}: $P < 0.001$ (***)<br>Ca^{2+} release: $P = 0.1681$ (ns)
**Table S4**	Paired two-tailed Student's *t*-test	pEC_{50}: $P = 0.9565$ (ns)<br>Ca^{2+} release: $P = 0.8442$ (ns)<br>h: $P = 0.3110$ (ns)
